# A New Dirichlet‐Multinomial Mixture Regression Model for the Analysis of Microbiome Data

**DOI:** 10.1002/sim.70220

**Published:** 2025-08-07

**Authors:** Roberto Ascari, Sonia Migliorati, Andrea Ongaro

**Affiliations:** ^1^ Department of Economics, Management and Statistics (DEMS) University of Milano‐Bicocca Milano Italy

**Keywords:** compound distribution, inter‐ and intraclass correlations, multivariate counts, spike and slab prior

## Abstract

Motivated by the challenges in analyzing gut microbiome and metagenomic data, this paper introduces a novel mixture distribution for multivariate counts and a regression model built upon it. The flexibility and interpretability of the proposed distribution accommodate both negative and positive dependence among taxa and are accompanied by numerous theoretical properties, including explicit expressions for inter‐ and intraclass correlations, thereby providing a powerful tool for understanding complex microbiome interactions. Furthermore, the regression model based on this distribution facilitates the clear identification and interpretation of relationships between taxa and covariates by modeling the marginal mean of the multivariate response (i.e., taxa counts). Inference is performed using a tailored Hamiltonian Monte Carlo estimation method combined with a spike‐and‐slab variable selection procedure. Extensive simulation studies and an application to a human gut microbiome dataset highlight the proposed model's substantial improvements over competing models in terms of fit, interpretability, and predictive performance.

## 1 Introduction

The human microbiome refers to the collection of genes associated with the microbiota, that is, the microbial community residing within the human body. This community includes bacteria, viruses, and some unicellular eukaryotes [1, 2]. It is well established that the microbiota outnumbers human cells and that the microbiome functions as an extended human genome—or a second genome—providing metabolic and genetic capabilities beyond those intrinsic to human cells alone [3, 4, 5]. Despite the generally beneficial mutualistic relationship between the microbiota and humans, disruptions in this balance can adversely affect health outcomes. For instance, alterations in gut microbiome composition have been linked to diabetes, cardiovascular disease, obesity, autoimmune disease, anxiety, colorectal cancer, and many other conditions affecting human health [1, 6, 7, 8]. Reviews by Libertucci and Young [9] and Lloyd‐Price et al. [10] provide further insights into these associations.

The recent surge in microbiome research is driven not only by its profound implications for medicine and health but also by advances in next‐generation sequencing technologies. These technologies enable researchers to analyze microbiome composition using direct DNA sequencing of marker genes or entire metagenomics without the need for burdensome isolation and culturing [11]. Among the most commonly used approaches to quantify bacterial abundance in a community are targeted amplicon sequencing and shotgun metagenomic sequencing [1, 12]. Both methodologies generate short DNA reads that, after pre‐processing, assembly, and alignment to reference databases, are classified into known taxa and clustered into operational taxonomic units (OTUs)—microbial “species”—based on DNA sequence similarity, typically at the 97% level. This clustering can be achieved through closed‐reference, open‐reference, or *de novo* strategies. More recently, amplicon sequence variants (ASVs)—which represent inferred single DNA sequences recovered from high‐throughput analyses—have gained popularity as units for strain‐level resolution.

The final data can be represented as a matrix reporting the count (or relative abundance) of each bacterial taxon in each sample. Here, we focus on counts (i.e., discrete models) to account for the different sizes of DNA materials across samples. An alternative approach is to normalize bacterial counts into proportions. However, inference based solely on proportions overlooks the fact that smaller samples inherently exhibit greater variability compared to larger ones. Furthermore, proportions often contain many zeros, whereas statistical methods for compositional data assume strictly positive proportions. Ad hoc adjustments are often applied to address this, but their impact on downstream analyses remains largely unexplored.

In addition to high dimensionality, microbiome data present several further distinctive challenges, including sparsity, complex correlation structures, and overdispersion. These issues arise because, while some taxa are common across samples, many others are present in only a few samples in much smaller quantities.

Numerous statistical methods have been developed to analyze microbiome data, including models designed to characterize community structure and to explore relationships between ASVs/OTUs and covariates. Most early studies employed exploratory statistical methods to search for natural patterns in microbiome data. For instance, ordination techniques such as principal component analysis were used to reduce dimensionality while preserving (Euclidean) distances between samples. Similarly, multidimensional scaling was often applied to summarize abundances and, in some cases, to link microbiome data with clinical, biological, or environmental predictors [13, 14].

More recent approaches adopt a generative modeling framework that directly models count data using sampling schemes. Some methods treat each ASV/OTU count individually, leveraging Poisson or negative binomial models (see Zhang et al. [15], Shuler et al. [16], and Jonsson et al. [17], among others). However, these approaches typically ignore the community structure of the microbiome.

Alternatively, joint modeling of all counts has gained traction, as it improves inference by borrowing strength across ASVs/OTUs. This is often achieved using a multinomial distribution, where the underlying parameters, that is, the proportions Π, are treated as random variables (compound multinomial models). A common choice is the Dirichlet distribution for Π, resulting in a Dirichlet‐multinomial (DM) distribution [18]. Building on this, some researchers have proposed DM regression models to analyze taxa counts more effectively. Chen and Li [19] developed a sparse group $l_{1}$ penalty penalized likelihood approach for variable selection, while Wadsworth et al. [20] addressed the same goal using a Bayesian framework. Pedone et al. [21] deployed a hierarchical Bayesian model building upon the DM regression framework, whereas Koslovsky [22] proposed a zero‐inflated DM model to address an excessive presence of zeros. Moreover, Harrison et al. [23] claimed the superiority of the DM model over alternatives in analyzing microbiome and other ecological count data.

Despite its popularity and its capacity to model overdispersed count data, the DM distribution often fails to capture the complexity of real microbiome datasets. This limitation arises from the rigid covariance structure imposed by its parameterization, which inherently imposes pairwise negative correlations. As a result, it cannot adequately describe the co‐occurrence and co‐absence relationships between microbial taxa.

To enhance flexibility in terms of dependence structure, various additional regression models have been developed (e.g., Xia et al. [24], Zhang et al. [25], Grantham et al. [26], and Ren et al. [27]). These models share a compound multinomial structure for the response variable but differ in the distribution assigned to the random multinomial parameter Π. However, none of these models regress the response mean vector directly onto covariates due to the analytical intractability of this vector. For instance, Zhang et al. [25] proposed a generalized Dirichlet distribution for Π [28] which provides an explicit expression for the mean. However, this mean is a complex function of the parameters, leading the authors to link covariates to parameters without clear interpretability. Xia et al. [24] and Grantham et al. [26] assumed a logistic normal distribution for Π [29] defined on log‐ratio transformations of Π. Unfortunately, the likelihood of the resulting model for taxa counts lacks a closed‐form expression. This induces difficulties both from a computational perspective and in the interpretation of the impact of the covariates on the response variable. Ren et al. [27] introduced a regression model within a Bayesian nonparametric framework based on dependent Dirichlet processes. Their approach is designed to incorporate sample‐specific latent factors.

We propose a new compound multinomial distribution that generalizes the DM, referred to as the extended flexible Dirichlet‐multinomial (EFDM), along with a regression model based on this distribution.

The EFDM model can be viewed as a structured DM mixture, that is, a special mixture entailing suitable links among the parameters of its mixture components. This structured approach distinguishes it from a general DM mixture model, which often suffers from challenges like over‐parameterization, lack of interpretability, and computational issues. In contrast, the EFDM exhibits crucial mathematical and computational properties, including identifiability, and explicit expressions for dependence measures. At the same time, the EFDM model demonstrates remarkable flexibility in capturing a diverse range of shapes, variability, and dependence structures.

This model addresses two fundamental objectives in microbiome analysis. First, it offers a comprehensive framework for exploring the dependence structure among bacterial taxa. Unlike the DM model, the EFDM admits positive correlations, enabling the modeling of co‐occurrence relationships between taxa [30]. Moreover, the EFDM enhances the interpretability of complex microbiome interactions by providing explicit measures of inter‐ and intraclass correlations, offering a more nuanced understanding of association patterns within and across bacterial genera. Second, the new regression model based on the EFDM distribution facilitates clear identification and interpretation of relationships between the ASVs/OTUs and covariates through regression of the marginal mean of the multivariate response (i.e., taxa counts). This is due to its flexible and interpretable parametric structure, which ensures accurate parameter inference, and clear detection of biological insights. Additionally, the mixture structure of the EFDM naturally accommodates excessive zeros, making it particularly suitable for microbiome datasets.

The remainder of this paper is organized as follows. Section 2 introduces an analysis of the general compound multinomial model, which encompasses both the DM distribution (2.1) and the newly proposed EFDM distribution (2.2 and 2.3). Particular attention is given to issues related to overdispersion as well as inter‐ and intraclass correlations. Section 3 describes the regression model based on the EFDM distribution. Specifically, Section 3.1 outlines a reparameterization of the EFDM tailored for regression purposes. The Bayesian estimation procedure is detailed in Section 3.2, while Section 3.3 discusses the variable selection approach based on “spike and slab” mixtures. An extensive simulation study is presented in Section 4. Here, an evaluation of the variable selection and parameter estimation procedures is given, together with a comparison between the EFDMReg model and a zero‐inflated model [22]. Section 5 applies our model to the human gut microbiome “COMBO” dataset, originally introduced by Wu et al. [31]. Section (5.1) focuses on analyzing correlations among taxa, while Section (5.2) examines associations between the gut microbiome and several covariates. Finally, Section 6 offers some concluding remarks. Proofs and supplementary analyses are provided in the Supporting Information (SM).

## 2 Compound Multinomial Distributions

Let Y be a D‐dimensional random vector (r.v.) with integer elements constrained to sum to a fixed positive integer n, that is, having support on the D‐part discrete simplex ${𝒮}_{n}^{D}=\left\{\text{y}={\left(y_{1},\ldots ,y_{D}\right)}^{⊺}:y_{r}\in \{0,1,\ldots ,n\},\sum_{r=1}^{D}y_{r}=n\right\}$. Note that, in our definition, the simplex—an (D−1)‐dimensional object—is embedded in a D‐dimensional space. In this way, all elements are treated symmetrically. The standard probability distribution for Y is the multinomial distribution M(n,π), which is characterized by the following probability mass function (p.m.f.):

$$f_{M}(\text{y};\pi )=\frac{n!}{\prod_{r=1}^{D}(y_{r}!)}\prod_{r=1}^{D}\pi_{r}^{y_{r}},\;\text{y}\in {𝒮}_{n}^{D}$$

where the parameter $\pi ={\left(\pi_{1},\ldots ,\pi_{D}\right)}^{⊺}$ is a D‐dimensional vector of probabilities lying in the D‐part continuous simplex ${𝒮}^{D}=\left\{\pi \in {\mathbb{R}}^{D}:\pi_{r}>0,\;\sum_{r=1}^{D}\pi_{r}=1\right\}$. Its first two moments can be expressed as

(1) $${𝔼}_{M}\left[\text{Y}\right]=n\pi \;\text{and}\;{𝕍}_{M}\left(\text{Y}\right)=n\text{M}(\pi )$$

where $\text{M}(\pi )=(\text{Diag}(\pi )-\pi \pi^{⊺})$ and Diag(π) defines a diagonal matrix whose diagonal entries are equal to π. This type of covariance matrix M(π), which will often appear in the following, entails a very restrictive dependence structure. Indeed, it is completely determined by the (scaled) mean vector π, and it only allows for negative pairwise correlations. This often leads to a poor fit to real datasets, in particular to microbiome data for which the actual variation is usually larger than would be predicted by the multinomial model.

Indeed, the fact that the multinomial distribution entails limited variability can be argued even from a theoretical point of view by resorting to the notion of normalized variance. For a bounded random variable Y, the range of possible values of its variance is heavily dependent on its mean. In particular, in the following, we shall be interested in bounded random variables taking values in the interval [0,q]. Therefore, the maximum variance for a given mean value μ is μ(q−μ), so that very large or very small values of μ imply low variances. This is the reason why, hereafter, to assess the variability of bounded random variables, we shall resort to the normalized variance

(2) $$\bar{V}(Y)=\frac{\mathrm{Var}\left(Y\right)}{\mu (q-\mu )}$$

The normalized variance of the multinomial distribution components is easily seen to be 1/n, which is quite low if n is large.

To account for a larger variation (i.e., overdispersion), and for a more flexible dependence structure, a promising alternative is based on a compound approach [18]. This assumes that the parameter Π of the multinomial distribution is random with distribution ℱ defined on ${𝒮}^{D}$, and it relies on the marginalization over ℱ of the joint distribution of Y and Π. In particular, the total expectation, variance, and covariance laws allow us to write the moments of Y in terms of moments of Π. Namely:

(3) 𝔼[Y]=nμand𝕍(Y)=n(M(μ)+(n−1)𝕍(Π))

where $\text{M}(\mu )=\left(\text{Diag}(\mu )-\mu \mu^{⊺}\right)$ and μ=𝔼[Π]. The main difference with respect to the multinomial variance in Equation (1) is represented by the extra term n(n−1)𝕍(Π), which introduces the desired flexibility, being dominant for large n. In particular, one has

$$\bar{V}(Y_{r})=\frac{1}{n}+\frac{n-1}{n}\;\bar{V}(\Pi_{r}),\;\;\;\;r=1,\ldots ,D$$

As a consequence, for any given $\mu_{r}$, $\bar{V}(Y_{r})$ can take any value in [1/n,1], thus capturing any degree of extra variation with respect to the multinomial. Moreover, it can be easily seen that

$$\lim_{n\to \infty }\text{Corr}\left(Y_{r},Y_{h}\right)=\text{Corr}\left(\Pi_{r},\Pi_{h}\right)$$

thus, an arbitrary dependence structure can be reached by the general compound multinomial model, including positive correlations, for a sufficiently large n.

We shall now introduce a different perspective on the general compound model, which is aimed at showing its potential and flexibility in modeling very crucial objects of investigation for microbiome data (i.e., inter‐ and intraclass correlations). More precisely, let ${\text{U}}_{j}={\left(U_{1j},\ldots ,U_{rj},\ldots ,U_{Dj}\right)}^{⊺}∼M(1,\pi )$ represent the outcome of the j‐th drawing (j=1,…,n), so that $\text{Y}=\sum_{j=1}^{n}{\text{U}}_{j}$. In this way, the sample counts Y are viewed as generated by n identically distributed drawings. When Y follows a multinomial distribution M(n,π), the ${\text{U}}_{j}$s are independent. This hinders the possibility of modeling co‐occurrences or co‐absences of bacteria, which are often encountered in applications, that is, $\text{Cov}\left(U_{rj},U_{r^{\prime }j^{\prime }}\right)=0$ for any two drawings $j\neq j^{\prime }$ and any two bacteria r and $r^{\prime }$.

A simple way to introduce dependence among the ${\text{U}}_{j}$s is to assume that they are exchangeable for any given n. Then, by De Finetti's theorem, conditional on a random vector Π=π, the ${\text{U}}_{j}$s are i.i.d. M(1,π). Thus, this sampling scheme is exactly the one generating the compound multinomial distribution. Therefore, it is of interest to investigate the ${\text{U}}_{j}$s dependence structure in terms of inter‐ and intraclass correlations. In the context of microbiome research, interclass correlation reflects between‐host variation by measuring the correlation between single drawings of different taxa. In contrast, intraclass correlation quantifies within‐host stability, as it captures the degree of similarity among units within the same group. It is easy to see that, for any two drawings $j\neq j^{\prime }$ and any two bacteria, r and $r^{\prime }$, the general covariance is

$$\text{Cov}\left(U_{rj},U_{r^{\prime }j^{\prime }}\right)=\text{Cov}\left(\Pi_{r},\Pi_{r^{\prime }}\right)$$

and the correlation is

(4) $$\text{Corr}\left(U_{rj},U_{r^{\prime }j^{\prime }}\right)=\text{Corr}\left(\Pi_{r},\Pi_{r^{\prime }}\right)\sqrt{\bar{V}(\Pi_{r})\bar{V}(\Pi_{r^{\prime }})}$$

Note that the interclass correlation (i.e., $r\neq r^{\prime }$) can take any value in [−1,1], thus modeling any co‐occurrence or co‐absence of bacteria. The intraclass correlation ($r=r^{\prime }$) is given by $\bar{V}(\Pi_{r})$, which entails any positive degree of dependence, in coherence with natural expectations.

As a byproduct of the exchangeable representation of the compound multinomial model, we obtain convergence of Y/n to Π. Specifically, the general De Finetti's representation theorem (see Theorem 1.49 in Schervish [32]) entails almost sure convergence of the empirical distribution of observations to a random probability measure P such that, conditionally on P, the observations are i.i.d. with distribution P. It is then not difficult to check that, in our framework, this is equivalent to

$$\frac{\text{Y}}{n}\overset{a.s.}{\to }\Pi$$

as n tends to infinity. Note that this implies convergence in distribution, and also the convergence of all joint moments as Y/n is a bounded r.v. The latter convergence was also proven by Mosimann [18] by direct calculation of the limiting joint moments.

### 2.1 The Dirichlet‐Multinomial Distribution

Compounding the multinomial distribution with the Dirichlet distribution leads to the well‐known DM distribution. More precisely, we use the following parameterization of the Dirichlet probability density function (p.d.f.):

(5) $$f_{\text{Dir}}(\pi ;\mu ,\alpha^{+})=\frac{\Gamma (\alpha^{+})}{\prod_{r=1}^{D}\Gamma (\mu_{r}\alpha^{+})}\prod_{r=1}^{D}\pi_{r}^{(\mu_{r}\alpha^{+})-1}$$

where $\mu =𝔼\left[\Pi \right]\in {𝒮}^{D}$, $\alpha^{+}>0$ represents a precision parameter, as the variance of each element of Π can be expressed as a decreasing function of $\alpha^{+}$, and Γ(·) denotes the Gamma function. As a consequence, the p.m.f. of the DM is

(6) $$f_{\text{DM}}(\text{y};\mu ,\alpha^{+})=\frac{n!\;\Gamma (\alpha^{+})}{\Gamma (n+\alpha^{+})}\prod_{r=1}^{D}\frac{\Gamma (y_{r}+(\mu_{r}\alpha^{+}))}{\left(y_{r}!)\;\Gamma (\mu_{r}\alpha^{+}\right)}$$

with $\text{y}\in {𝒮}_{n}^{D}$ and mean vector ${𝔼}_{\text{DM}}\left[\text{Y}\right]=n\mu$. To derive the covariance matrix of the DM using Formula (3), it is useful to write the Dirichlet covariance matrix as

$${𝕍}_{\text{Dir}}\left(\Pi \right)=\frac{\text{M}(\mu )}{\alpha^{+}+1}$$

with $\text{M}(\mu )=\left(\text{Diag}(\mu )-\mu \mu^{⊺}\right)$. Thus, the Dirichlet distribution has the same type of very restrictive covariance matrix as the multinomial, with just an extra parameter $\alpha^{+}$ modeling the overall variability. As a consequence, the covariance matrix of the DM can be written as

(7) $${𝕍}_{\text{DM}}\left(\text{Y}\right)=n\text{M}(\mu )\left[1+\frac{n-1}{\alpha^{+}+1}\right]$$

Therefore, the DM maintains the same negative type of dependencies of the multinomial distribution, leading, in particular, to the same correlations between counts. The gain of the DM over the multinomial distribution is the additional parameter $\alpha^{+}$, which allows completely flexible modeling of the variability in the sense that the normalized variance ${\bar{V}}_{\text{DM}}(Y_{r})=\frac{1}{n}+\frac{n-1}{n}\frac{1}{\alpha^{+}+1}$ has range (1/n,1). However, note that this model entails the same normalized variance for all the counts $Y_{r}$, since ${\bar{V}}_{\text{DM}}(Y_{r})$ does not depend on r. Although this additional parameter greatly improves the fit of the model (e.g., see Chen and Li [19] and Wadsworth et al. [20]), the DM model is still often too rigid to deal with real complex datasets, as we shall show.

Let us now investigate the DM distribution in terms of inter‐ and intraclass correlations (see Formula (4)). The intraclass coefficient is equal to $1/(\alpha^{+}+1)$. This positive dependence is also responsible for the “overdispersion” of the DM with respect to the multinomial, which is exactly determined by this quantity as it emerges from Formula (7). The interclass coefficients

$${\text{Corr}}_{\text{DM}}\left(U_{rj},U_{r^{\prime }j^{\prime }}\right)=-\frac{1}{\alpha^{+}+1}\sqrt{\frac{\mu_{r}}{1-\mu_{r}}\frac{\mu_{r^{\prime }}}{1-\mu_{r^{\prime }}}},\;r\neq r^{\prime }$$

are all negative, therefore accounting only for the co‐absences of the bacteria.

### 2.2 The Extended Flexible Dirichlet‐Multinomial Distribution

To obtain a richer distribution for count vectors on ${𝒮}_{n}^{D}$, we consider the extended flexible Dirichlet (EFD) [33] as the distribution for Π on ${𝒮}^{D}$. The latter is a structured finite mixture with Dirichlet‐distributed components, sharing some constraints on the parameters. The p.d.f. of the EFD distribution can be expressed according to the following mixture representation:

(8) $$f_{\text{EFD}}(\pi ;\alpha ,\tau ,\text{p})=\sum_{r=1}^{D}p_{r}f_{\text{Dir}}(\pi ;\lambda_{r},\alpha^{+}+\tau_{r})$$

where $f_{\text{Dir}}(\cdot ;\cdot )$ is given by (5), the component‐specific mean vectors are defined as

(9) $$\lambda_{r}=\frac{\alpha +\tau_{r}{\text{e}}_{r}}{\alpha^{+}+\tau_{r}},\;r=1,\ldots ,D$$

α and τ are two vectors defined on ${\mathbb{R}}_{+}^{D}$, $\alpha^{+}=\sum_{r=1}^{D}\alpha_{r}$, $\text{p}={\left(p_{1},\ldots ,p_{D}\right)}^{⊺}$ is such that $0\leq p_{r}<1$ and $\sum_{r=1}^{D}p_{r}=1$, and ${\text{e}}_{r}$ is the D‐dimensional vector with zero elements except for the r‐th that is equal to one. Moreover, $\tau_{r}$ is set (arbitrarily) equal to 1 whenever $p_{r}=0$. Equation (9) highlights that $\lambda_{r}$ can be written as a weighted average of a common barycenter $\bar{\alpha }=\alpha /\alpha^{+}$ and the r‐th simplex vertex ${\text{e}}_{r}$, namely, $\lambda_{r}=(1-w_{r})\bar{\alpha }+w_{r}{\text{e}}_{r}$, where

(10) $$w_{r}=\frac{\tau_{r}}{\alpha^{+}+\tau_{r}}$$

This alternative representation allows us to geometrically interpret the link (i.e., the constraint) among the component means: $\lambda_{r}$ departs from the common barycenter $\bar{\alpha }$ in the direction of the vertex ${\text{e}}_{r}$ by a factor controlled by $w_{r}$ (or, equivalently, by $\tau_{r}$). Furthermore, each mixture component has its own precision parameter, which is greater than $\alpha^{+}$.

Unlike the Dirichlet distribution, the EFD can model arbitrarily large positive correlations between elements of Π [33].

The new EFDM distribution on ${𝒮}_{n}^{D}$, denoted by EFDM(α,τ,p), can be obtained by compounding the multinomial distribution with the EFD. Its p.m.f. takes the form:

(11) $$f_{\text{EFDM}}(\text{y};\alpha ,\tau ,\text{p})=\frac{n!}{\prod_{l=1}^{D}(y_{l}!)}\left(\prod_{l=1}^{D}\alpha_{l}^{\left[y_{l}\right]}\right)\sum_{r=1}^{D}p_{r}\frac{1}{{\left(\alpha^{+}+\tau_{r}\right)}^{\left[n\right]}}\frac{{\left(\alpha_{r}+\tau_{r}\right)}^{\left[y_{r}\right]}}{\alpha_{r}^{\left[y_{r}\right]}}$$

where $y^{\left[n\right]}=y(y+1)\ldots (y+n-1)$ is the rising factorial function and $y^{\left[0\right]}=1$. Quite interestingly, the p.m.f. (11) can be expressed as a finite mixture with DM components, namely:

(12) $$f_{\text{EFDM}}(\text{y};\alpha ,\tau ,\text{p})=\sum_{r=1}^{D}p_{r}f_{\text{DM}}(\text{y};\lambda_{r},\alpha^{+}+\tau_{r})$$

where $f_{\text{DM}}(\cdot ;\cdot )$ and $\lambda_{r}$ are given by Equations (6) and (9), respectively.

It is worth noting that the EFDM distribution includes the DM and the multinomial distributions as particular cases. More precisely, when $\tau_{r}=1$ and $p_{r}=\alpha_{r}/\alpha^{+}$ for every r=1,…,D, then (11) coincides with (6), so that the DM is recovered. If, in addition, the precision parameter $\alpha^{+}$ diverges, then the DM (and consequently the EFDM) reduces to the multinomial distribution.

The EFDM shares the same type of mixture structure (8) of the EFD model, and consequently, many properties of the latter model are inherited by the EFDM. For example, it maintains its flexibility in terms of shapes, allowing for multimodality (see Section S1.1 of the SM for an illustration of multimodality via a ternary diagram).

Another property of the EFDM model is strong identifiability, which requires that two elements of a parametric family of distributions are equal if and only if the corresponding parameters are identical. It is well‐known that general mixture models are typically not identifiable, which may cause inferential difficulties, such as in estimation. A typical source of non‐identifiability in mixture models is invariance under permutations of the component labels of the mixture. Another typical lack of identifiability comes from overfitting (i.e., using an undue number of components), thus implying some null mixing weights.

The following proposition shows that our model, although allowing for some null weights, exhibits the property of strong identifiability.

Proposition 1 *Let* Y∼EFDM(θ) *and* ${\text{Y}}^{\prime }∼\text{EFDM}(\theta^{\prime })$, *where* $\theta ={\left(\alpha ,\tau ,\text{p}\right)}^{⊺}$ *and* $\theta^{\prime }={\left(\alpha^{\prime },\tau^{\prime },{\text{p}}^{\prime }\right)}^{⊺}$. *Then* $\text{Y}∼{\text{Y}}^{\prime }$ *if and only if* $\theta =\theta^{\prime }$.

The proof is presented in Section S1.2 of the SM.

The (scaled) mean vector μ of the EFDM can be expressed as a weighted mean of the component‐specific barycenters $\lambda_{r}$ defined in Equation (9), namely:

(13) $$\mu ={𝔼}_{\text{EFDM}}\left[\text{Y}/n\right]=\sum_{r=1}^{D}p_{r}\lambda_{r}$$

and it represents the probability vector of the D taxa under study.

The covariance matrix of the EFDM can be computed by exploiting the relationship between the moments of Y and Π given by (3), together with the moments of Π reported in Section S1.3 of the SM for ease of reference, and it takes the form:

(14) $$\begin{array}{cc} {𝕍}_{\text{EFDM}}\left(\text{Y}\right) & =n\text{M}(\mu )\left[1+(n-1)\left(1-\frac{k_{2}}{k_{1}^{2}}\right)\right]+n(n-1)\left[\frac{k_{2}}{k_{1}^{2}}\text{M}(\text{d})+\Psi +\text{W}\right] \end{array}$$

where

(15) $$k_{1}=\sum_{r=1}^{D}\frac{p_{r}}{\phi_{r}},\;\;\;\;k_{2}=\sum_{r=1}^{D}\frac{p_{r}}{\phi_{r}(\phi_{r}+1)},\;\;\;\;\text{with}\;\;\;\;\phi_{r}=\alpha^{+}+\tau_{r}$$

and

$$\Psi =\text{Diag}(\psi ),\;\;\;\;\;\;\;\;\text{W}=\alpha \gamma^{⊺}+\gamma \alpha^{⊺}$$

with d, ψ, and γ representing vectors with elements $d_{r}=\frac{p_{r}\tau_{r}}{\phi_{r}}$, $\psi_{r}=\alpha_{r}\left(\frac{k_{2}}{k_{1}}-k_{1}+k_{2}\right)-\frac{\alpha^{+}d_{r}}{\phi_{r}+1}$, and $\gamma_{r}=d_{r}\left(\frac{1}{\phi_{r}+1}-\frac{k_{2}}{k_{1}}\right)$, respectively. We recall that for a generic vector a, $\text{M}(\text{a})=(\text{Diag}(\text{a})-\text{a}{\text{a}}^{⊺}$).

Concerning the DM corresponding expression (7), we note two main differences, namely, the coefficient multiplying the matrix M(μ) and the newly introduced matrices M(d), Ψ, and W. For the former, the positive coefficient ${\left(\alpha^{+}+1\right)}^{-1}$ is replaced by $\left(1-k_{2}/k_{1}^{2}\right)$ which can take on both positive and negative values (for a proof of this result and an analysis of the new matrices, see Section S1.4 of the SM).

In essence, as the correlations of the EFD model can be both negative and positive with arbitrarily large values [33], and as, by Formula (3), the leading term of the EFDM variances and covariances is the EFD covariance matrix ${𝕍}_{\text{EFD}}\left(\Pi \right)$, the EFDM correlations maintain this flexibility for large n. However, it is important to note that the parsimonious parametrization of the EFDM model does not permit the representation of a fully general covariance structure. This limitation arises from the fact that the number of parameters increases linearly with D, rather than quadratically, as required to model an arbitrary covariance matrix. Nonetheless, this linear growth significantly reduces the computational burden, particularly when D is large.

Explicit expressions for the intra‐ and interclass correlations are as follows

(16) $${\text{Corr}}_{\text{EFDM}}\left(U_{rj},U_{rj^{\prime }}\right)=\left(1-\frac{k_{2}}{k_{1}^{2}}\right)+\frac{\frac{k_{2}}{k_{1}^{2}}d_{r}(1-d_{r})+\psi_{r}+2\gamma_{r}\alpha_{r}}{\mu_{r}(1-\mu_{r})}$$

and for $r\neq r^{\prime }$

(17) $$\begin{array}{ll} {\text{Corr}}_{\text{EFDM}}\left(U_{rj},U_{r^{\prime }j^{\prime }}\right) & =-\sqrt{\frac{\mu_{r}}{1-\mu_{r}}\frac{\mu_{r^{\prime }}}{1-\mu_{r^{\prime }}}}\;\left[\left(1-\frac{k_{2}}{k_{1}^{2}}\right)+\left.\frac{\frac{k_{2}}{k_{1}^{2}}d_{r}d_{r^{\prime }}-\alpha_{r}\gamma_{r^{\prime }}-\alpha_{r^{\prime }}\gamma_{r}}{\mu_{r}\mu_{r^{\prime }}}\right]\right. \end{array}$$

Note that the EFDM's intraclass correlations (16) are not fixed, but they may vary for different bacteria (i.e., they depend on r), unlike the DM model. The interclass correlations (17), being proportional to the EFD correlations by Formula (4), may take arbitrarily large negative as well as positive values.

### 2.3 The Flexible Dirichlet‐Multinomial Distribution

The EFDM distribution includes a relevant sub‐model obtained by imposing $\tau_{r}=\tau$ for every r=1,…,D. Indeed, under this parametric constraint, the EFD distribution of Π coincides with the flexible Dirichlet (FD), which displays special properties [34, 35]. Compounding the multinomial with the FD distribution leads to the definition of the flexible Dirichlet‐multinomial (FDM). The latter is a multivariate generalization of the flexible beta‐binomial (FBB) [36] and can be expressed as a finite mixture with DM components sharing the same precision parameter.

The FDM model is halfway between the EFDM and the DM in terms of both complexity (e.g., number of parameters) and flexibility. In particular, the FDM's covariance matrix can be written in a simple form as

(18) $${𝕍}_{\text{FDM}}\left(\text{Y}\right)=n\text{M}(\mu )\left[1+\frac{n-1}{\phi +1}\right]+n\frac{(n-1)\tau^{2}}{\phi (\phi +1)}\text{M}(\text{p})$$

where $\phi =\alpha^{+}+\tau$ is the (common) precision parameter. It is noteworthy that (18) greatly simplifies the corresponding matrix of the EFDM in Equation (14), since, under the FDM model, the matrix W contains null entries, and the term $\frac{k_{2}}{k_{1}^{2}}\text{M}(\text{d})+\Psi$ simplifies to $\tau^{2}\text{M}(\text{p})/[\phi (\phi +1)]$. While this simplification enhances computational efficiency, it comes at the cost of being characterized by only negative correlations. However, when compared to the covariance matrix (7) of the DM, it becomes clear that the new term proportional to M(p) enriches the dependence structure by introducing D additional parameters. As a result, the covariance matrix is no longer proportional to the multinomial one, as is the case for the DM. This allows the normalized marginal variances to vary across counts (i.e., bacterial taxa), while the correlations between counts are no longer solely dependent on the mean counts, as seen in the DM model.

The enhanced flexibility of the FDM distribution over the DM, along with its simplification compared to EFDM, is most evident when inspecting the expression for the intra‐ and interclass correlations. Specifically, the intraclass correlation is

(19) $${\text{Corr}}_{\text{FDM}}\left(U_{rj},U_{rj^{\prime }}\right)=\frac{1}{\phi +1}\left[1+\frac{\tau^{2}}{\phi }\frac{p_{r}(1-p_{r})}{\mu_{r}(1-\mu_{r})}\right]$$

and the interclass correlation is

(20) $${\text{Corr}}_{\text{FDM}}\left(U_{rj},U_{r^{\prime }j^{\prime }}\right)=-\frac{1}{\phi +1}\sqrt{\frac{\mu_{r}}{1-\mu_{r}}\frac{\mu_{r^{\prime }}}{1-\mu_{r^{\prime }}}}\left[1+\frac{\tau^{2}}{\phi }\frac{p_{r}p_{r^{\prime }}}{\mu_{r}\mu_{r^{\prime }}}\right],\;r\neq r^{\prime }$$

Comparing these formulas with the corresponding formulas for the DM, one can see that the intraclass correlations may vary with the class (bacterium), and the interclass correlations, despite being still negative, allow for larger flexibility.

## 3 Regression Model and Inferential Issues

In this section, we present the development of the extended flexible Dirichlet‐multinomial regression (EFDMReg) model, starting with a suitable reparameterization of the EFDM to make it applicable to regression settings (3.1). Building on this foundation, we propose a Bayesian framework to address inference for the EFDMReg model (3.2) and outline the variable selection procedure employed, which is based on a spike‐and‐slab prior approach (3.3).

### 3.1 The New Regression Model

For a clearer understanding of how covariates influence the overall response, we directly link the marginal response mean to the covariates rather than separately modeling the means of the mixture components, as is typically done in mixture regression models. To achieve this, we adopt an alternative parameterization of the EFDM model that explicitly incorporates the mean vector μ=𝔼[Y/n] as defined in Equation (13). Therefore, we can take advantage of the parameterization consisting of μ, $\alpha^{+}$, the vector of mixing weights p, and the vector $\text{w}={\left(w_{1},\ldots ,w_{D}\right)}^{⊺}$ whose elements are defined in Equation (10), which controls the extent to which the means of the mixture components $\lambda_{r}$s are far apart from each other. Nevertheless, this parameterization is not variation‐independent since the constraints $p_{r}w_{r}<\mu_{r}$ (r=1,…,D) must be satisfied. The absence of variation independence may cause inferential problems; however, it can be removed by resorting to a further reparameterization that replaces the $w_{r}$s with their normalized version. More precisely, since it can be easily shown that $w_{r}\in \left(0,\min\left\{1,\frac{\mu_{r}}{p_{r}}\right\}\right)$, we define

(21) $${\tilde{w}}_{r}=\frac{w_{r}}{\min\left\{1,\frac{\mu_{r}}{p_{r}}\right\}}\in (0,1)$$

Therefore, we shall adopt the final parameterization of the EFDM$\left(\mu ,\alpha^{+},\text{p},\tilde{\text{w}}\right)$ where $\mu \in {𝒮}^{D}$, $\text{p}={\left(p_{1},\ldots ,p_{D}\right)}^{⊺}$ such that $0\leq p_{r}<1$ and $\sum_{r=1}^{D}p_{r}=1$, $\alpha^{+}>0$ and $\tilde{\text{w}}={\left({\tilde{w}}_{1},\ldots ,{\tilde{w}}_{D}\right)}^{⊺}$ with ${\tilde{w}}_{r}\in (0,1)$, where we conventionally set ${\tilde{w}}_{r}=1/2$ if $p_{r}=0$.

To define the new regression model, let us consider a set of independent microbiome responses $\text{Y}={\left({\text{Y}}_{1},\ldots ,{\text{Y}}_{i},\ldots ,{\text{Y}}_{N}\right)}^{⊺}$ collected on a group of N samples. For the i‐th sample, ${\text{Y}}_{i}\in {𝒮}_{n_{i}}^{D}$ counts the number of times each of the D taxa occurred among $n_{i}$ bacterial reads, and ${\text{x}}_{i}$ is a (K+1)‐dimensional vector of subject‐specific covariates. To regress the mean vector $\mu_{i}=𝔼\left[{\text{Y}}_{i}/n_{i}\right]\in {𝒮}^{D}$ onto the covariates, a smooth and invertible link function $g(\cdot ):{𝒮}^{D}\mapsto {\mathbb{R}}^{\left(D-1\right)}$ must be chosen. A fruitful choice is the multinomial logit‐link function, defined as

(22) $$g(\mu_{ir})=\log\left(\frac{\mu_{ir}}{\mu_{iD}}\right)={\text{x}}_{i}^{⊺}\beta_{r},\;r=1,\ldots ,D-1$$

where $\beta_{r}={\left(\beta_{r0},\beta_{r1},\ldots ,\beta_{rK}\right)}^{⊺}$ is a vector of regression coefficients for the r‐th element of $\mu_{i}$. The strength of this choice mainly lies in the ease of interpretation of the regression coefficients in terms of the log‐odds ratios of each category with respect to the baseline category. Note that formulation (22) assumes that the last category has been chosen as the baseline, thus implying that $\beta_{D}=\text{0}$. Nonetheless, any category could be selected as the baseline, due to the symmetry of the EFDM model in its elements, leading the corresponding vector of coefficients to be the null vector.

We remark that our choice to model the response mean μ using a multinomial link function contrasts with most of the literature on the DM regression model, where a log‐linear function of the standard parameter $\mu \alpha^{+}$ (see Formula (6)) is typically employed. The log‐linear formulation may pose interpretability challenges, as each element of the vector $\mu \alpha^{+}$ jointly affects both the mean and the variance of the counts. In particular, the signs of the corresponding regression coefficients do not generally determine the type of associations (positive or negative) between the covariates and the mean of the counts. For further details, see the discussion in the introduction to Section S3 of the SM.

By assuming that ${\text{Y}}_{i}$ follows an EFDM($\mu_{i},\alpha^{+},\text{p},\tilde{\text{w}}$) distribution, we obtain the EFDMReg model. Clearly, if ${\text{Y}}_{i}$ is $M(n_{i},\mu_{i}$) or DM($\mu_{i},\phi$), with ϕ representing a precision parameter, distributed, the multinomial regression (MultReg) and the Dirichlet‐multinomial regression (DMReg) are then specified.

It is noteworthy that, though the main goal of the EFDMReg model is to describe the effect of the covariates on the general mean μ, both inter‐ and intraclass correlations (Formulas (16) and (17)) as well as component‐specific means $\lambda_{r}$s (see (9)) depend on μ. Therefore, all these quantities are also naturally modeled as functions of biological and environmental covariates. Finally, we underline that the above‐described EFDMReg model only regresses the mean parameter μ onto covariates. Indeed, this regression framework can be easily enriched by linking also the remaining parameters $\alpha^{+}$, p, and $\tilde{\text{w}}$ to (possibly overlapping) covariates through proper link functions. This allows the model to be adapted to specific contexts without particular difficulties.

### 3.2 Bayesian Inference

From an inferential perspective, the finite mixture structure of the EFDM model can be profitably dealt with by means of a Bayesian approach. The latter allows for overcoming many computational and analytical mixture‐related difficulties. In the following, we resort to Monte Carlo techniques and, in particular, to the Hamiltonian Monte Carlo (HMC) algorithm [37], which is a generalization of the Metropolis‐Hastings combining classical Markov Chain Monte Carlo (MCMC) and deterministic simulation methods.

To simulate the posterior distribution of $\eta ={\left(\beta_{1},\ldots ,\beta_{D-1},\alpha^{+},\text{p},\tilde{\text{w}}\right)}^{⊺}$, knowledge of the full joint distribution of (Y,η) is needed. The latter can be written as the product of the full likelihood and the prior distribution. The likelihood is simply based on N independent drawings from ${\text{Y}}_{i}∼\text{EFDM}(\mu_{i},\alpha^{+},\text{p},\tilde{\text{w}})$, where $\mu_{i}$ depends on the regression coefficients as specified in Equation (22). As for the prior distribution of η, the variation independence of the parameter space (prompted by the reparameterization illustrated in Section 3.1) greatly simplifies the specification of the joint prior distribution by allowing us to assume prior independence. Moreover, we use noninformative or weakly informative priors to induce minimum impact on the posterior distributions and thus select the following priors:

1. $\beta_{r}∼N_{K+1}(\text{0},\sum )$, where 0 is the (K+1)‐dimensional vector with zero elements and ∑ is a diagonal matrix with “large” variance values,
2. $\alpha^{+}∼$ Gamma(k·g,g) for “small” values of the rate hyperparameter g so to induce a large variability around the prior mean k,
3. a uniform distribution on ${𝒮}^{D}$ for p (i.e., a Dirichlet distribution with mean vector ${\left(1/D,\ldots ,1/D\right)}^{⊺}$ and precision equal to D),
4. ${\tilde{w}}_{r}∼$ Beta$\left(\delta_{1},\delta_{2}\right)$, r=1,…,D, with $\delta_{1}=\delta_{2}$ so to consider a symmetric prior.

Note that a subset of these priors can be adopted for the MultReg (namely, prior 1) and the DMReg (priors 1 and 2) models.

Of course, when prior information is available on some parameters, the corresponding prior distributions can be modified accordingly. For example, in the presence of many taxa displaying all low counts, excluding the corresponding mixture components may be beneficial to the estimation process. This can be achieved by concentrating the priors of the relative weights on the zero value. Furthermore, when D is large and only a few components of the mixture are expected to be present, a sparse prior on the weights $p_{r}$ can be adopted to favor the identification of these components by the inferential process. For example, one may choose a symmetric Dirichlet prior with parameters all equal to c/D for a suitable constant c as proposed in Section 22.4 of Gelman et al. [38].

To obtain simulated samples from the posterior distribution, we implemented the HMC method employing the Stan modeling language [39] (the code used to implement our method is publicly available in the following GitHub repository: https://github.com/robertoascari/EFDMReg). Moreover, Section S4.1 of the SM provides a detailed, step‐by‐step guide for estimating the EFDMReg model using the rstan package.

Furthermore, the fit of the models was compared via Watanabe‐Akaike information criterion (WAIC) [40, 41], a fully Bayesian criterion that balances between goodness‐of‐fit and complexity of a model: Lower values of WAIC indicate a better fit.

### 3.3 Variable Selection

Microbiome datasets are often characterized by high dimensionality. Indeed, depending on the aggregation level, researchers may handle scenarios with large values of the number of taxa D, and/or large values of the number of covariates K. The latter case is often problematic since it typically gives rise to interpretability as well as estimation issues. Interpretability issues are due to the possible irrelevant association between a subset of explanatory variables and the microbiome composition. Furthermore, especially when dealing with K>>D, problems may arise in the estimation procedure, and ad hoc strategies must be taken into account. In this framework, it is fundamental to perform variable selection (VS) by looking for the subset of P≤K covariates $X_{1}^{*},\ldots ,X_{P}^{*}$ that truly affect the mean response. One could consider the “best” (according to some criterion) model among all the $2^{K}$ models that are restrictions of the original model with K covariates; however, this is unfeasible when the number of covariates is large.

Here, we propose to resort to the (Bayesian) “spike and slab” mixture [42] VS method by adapting it to our multivariate context. Indeed, when the response variable is unidimensional, the regression coefficients $\beta_{k}$ (k=1,…,K) are endowed with the following prior:

(23) $$\begin{array}{cc} \beta_{k}|\gamma_{k} & ∼\gamma_{k}N\left(0,\sigma_{1}^{2}\right)+(1-\gamma_{k})\delta_{0},\\ \gamma_{k} & ∼\text{Bern}(\theta_{k}) \end{array}$$

where $\gamma_{k}\in \{0,1\}$ is a latent (i.e., unobservable) Bernoulli random variable, and $\delta_{0}$ denotes a degenerate distribution at zero. The slab and the spike are represented by the first and the second mixture components in Equation (23), respectively.

When dealing with a multidimensional response vector Y, such as in the EFDMReg model, one can generalize this idea in different ways. Indeed, each element $\mu_{ir}$ of the response mean vector $\mu_{i}$ in Equation (22) is linked to covariates through a different vector of regression coefficients $\beta_{r}$ (i=1,…,N; r=1,…,D−1). Then, a covariate $X_{k}$ (k=1,…,K) may provide a significant effect in all of the (D−1) equations (i.e., $\beta_{rk}\neq 0$ ∀r), in no equation ($\beta_{rk}=0$ ∀r), or in a subset of equations ($\beta_{rk}\neq 0$ for some r∈{1,…,D−1}).

In the following, we shall define a VS that identifies a covariate as relevant if it provides significant effects in at least one element of the response mean vector, as suggested in Brown et al. [43].

More precisely, let $\beta_{k}^{*}={\left(\beta_{1k},\beta_{2k},\ldots ,\beta_{(D-1)k}\right)}^{⊺}$ be the vector of regression coefficients associated with the k‐th covariate within the D−1 equations in (22) for k∈{0,1,…,K} ($\beta_{0}^{*}$ refers to the vector of intercepts). To perform an automatic VS, we shall consider the following set of priors for the regression coefficients:

(24) $$\beta_{0}^{*}∼N_{D-1}\left(\text{0},\tau^{2}I_{\left(D-1\right)}\right)$$

(25) $$\beta_{k}^{*}|\gamma_{k}∼\gamma_{k}N_{D-1}\left(\text{0},\sigma_{1}^{2}I_{\left(D-1\right)}\right)+(1-\gamma_{k})N_{D-1}\left(\text{0},\sigma_{0}^{2}I_{\left(D-1\right)}\right),\;k=1,\ldots ,K$$

(26) $$\gamma_{k}∼\text{Bern}(\theta_{k})$$

where $I_{\left(D-1\right)}$ is the identity matrix of order (D−1), and $\sigma_{1}^{2}$ and $\sigma_{0}^{2}$ in Equation (25) represent “large” and “small” variances, respectively. The first and the second normal mixture components in Equation (25) can be thought of as the slab and the spike, respectively. This is because, when $\gamma_{k}=0$, the prior distribution of $\beta_{k}^{*}$ is highly concentrated around the null vector, suggesting that the k‐th covariate does not affect the mean vector μ in any of its elements and can therefore be excluded. In contrast, when $\gamma_{k}=1$, the high variance $\sigma_{1}^{2}$ allows the elements of $\beta_{k}^{*}$ to take nonzero values. Thus, the prior (25) induces sparsity of the regression coefficients. In particular, a covariate will be considered significant if the posterior mean of the probability $\theta_{k}$ exceeds a predefined threshold.

The prior (24) for $\beta_{0}^{*}$ is a classical multivariate normal distribution with independent components and high common variances $\tau^{2}$, to provide a minimum impact on the posterior distribution.

Regarding our choice of variable selection mechanism, we adopted a procedure that evaluates the inclusion or exclusion of each covariate based on its impact on all the response variables, instead of considering a covariate‐taxon specific evaluation of presence/absence.

On one hand, this method simplifies the model by significantly reducing the number of parameters, especially when D is large. On the other hand, it does not lead to a substantial loss of flexibility in the selection process, still allowing, in particular, the possibility that a covariate affects only a few response variables. Indeed, when a covariate is included, the prior on its associated regression coefficient vector is diffuse and centered at zero (vague prior). This means that the inclusion of a covariate does not suggest its significance for all response variables; rather, in this case, the estimation is primarily data‐driven, allowing many regression coefficients to remain effectively null.

Moreover, this choice enables the detection of scenarios where a covariate has a moderate effect on multiple response variables, which is an aspect that is more challenging to capture in models where the inclusion/exclusion mechanism is taxon‐covariate specific. These considerations are confirmed by the simulations of Section 4.1.

Once the VS process is fulfilled, we produce the final estimate of all the regression coefficients, resorting to the priors specified in Section 3.2.

## 4 Simulation Studies

In this section, we summarize the simulation findings (a complete set of results can be found in Sections S2 and S3 of the SM), which serve two main objectives. The first objective is to evaluate the EFDMReg model in terms of variable selection, parameter estimation, correlation recovery, and scalability (Sections 4.1 and 4.2). The second is to compare the performance of the EFDMReg model with the zero‐inflated Dirichlet‐multinomial (ZIDM) proposed by Koslovsky [22], specifically under scenarios characterized by an abundance of zeros (Section 4.3). The ZIDM represents a versatile framework capable of addressing various challenges in microbiome data analysis while achieving competitive performance relative to state‐of‐the‐art alternatives.

### 4.1 Variable Selection Performance

Concerning variable selection, we set regression coefficients so that some covariates do not affect any taxa, some other covariates only a subset of taxa, and the remaining ones all taxa. In the following, we report a detailed description of one representative case with K=9 covariates and D=3 bacterial taxa (Case I), but similar results were obtained in more complex settings (see Section S2.1 of the SM for additional results, including Case II involving K=20 and D=3 and Case III with K=100 and D=15, which replicates conditions similar to the ones of the COMBO application presented in Section 5). Specifically, in Case I, we implemented a simulation study composed of B=300 replications. For each replication, we generated N=750 samples. For sample i, we read $n_{i}∼Po(50)$ bacteria and collected K additional covariates $x_{1},\ldots ,x_{K}$. Assuming standardized covariates, we generated them independently from N(0,1). Response vectors were generated according to an EFDMReg model.

Moreover, we set the following parameters:

$$\beta =\left[\beta_{0}^{*}\;|\;\beta_{1}^{*}\;|\;\beta_{2}^{*}\;|\;\cdots \;|\;\beta_{9}^{*}\right]=\left[\begin{array}{llllllllll} -\;4 & 0 & -3 & -3 & 2.7 & 0 & 3 & 0 & 0 & 0\\ 1.5 & 0 & -2 & 0.5 & -3 & 0 & 2.5 & 0 & 0 & 0\\ 0 & 0 & 0 & 0 & 0 & 0 & 0 & 0 & 0 & 0 \end{array}\right]$$

where $\beta_{k}^{*}$ is the vector of regression coefficients associated with the k‐th covariate, as specified in Section 3.3, $\alpha^{+}=50,\text{p}={\left(0.25,0.25,0.5\right)}^{⊺}$ and $\tilde{\text{w}}={\left(0.6,0.8,0.7\right)}^{⊺}$. Within the setting of β, we include three covariates (the second, the fourth, and the sixth) having an impact on both the equations defining the mean vector as in Equation (22) and one covariate (the third) affecting mainly the first equation.

**FIGURE 1:**
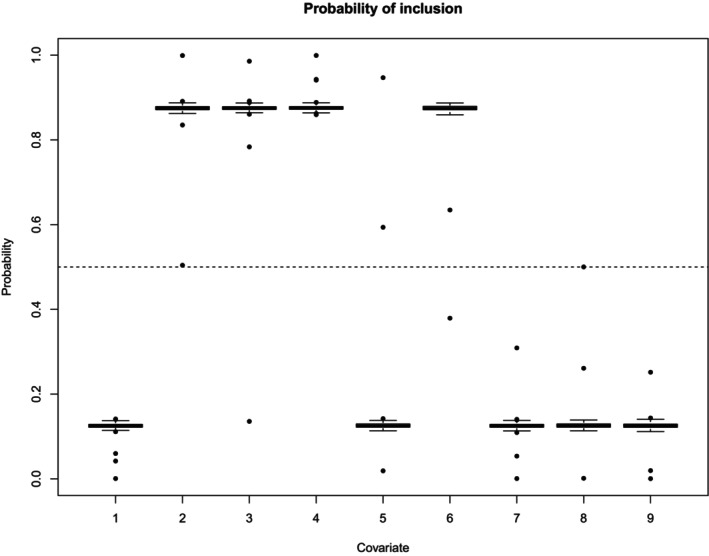
Case I—Boxplot of the posterior mean of the probability of inclusion for each covariate across replications.

Then, for each replication, we fitted the EFDMReg model with the spike and slab priors described in Section 3.3 and analyzed the posterior distribution of each $\theta_{k}$ (i.e., the probability that the k‐th covariate is included in the model). To summarize results for all the B=300 replications, Figure 1 reports the boxplots of the B posterior means of the probability of inclusion $\theta_{k}$'s for each covariate. These boxplots show very few cases of incorrect selection, thus implying sensitivity and specificity values close to 1.

**FIGURE 2:**
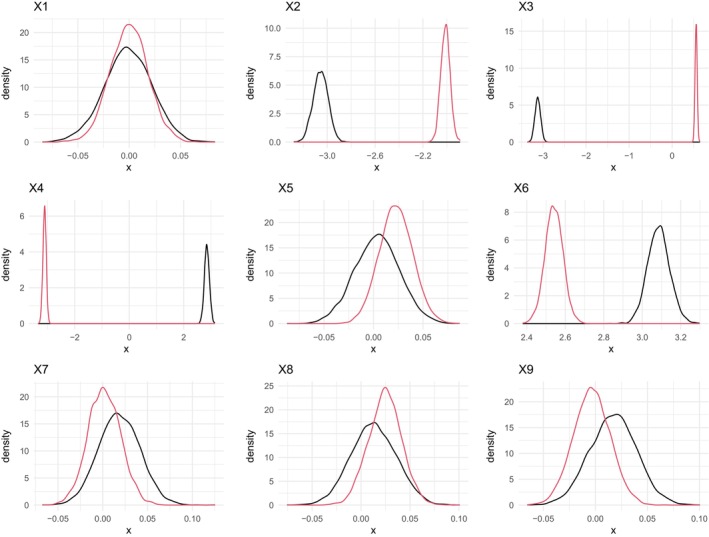
Case I—Posterior distribution of regression coefficients from a randomly selected MC replication. Black and red curves refer to the first and second elements of the mean vector, respectively.

Moreover, Figure 2 shows, for a randomly selected replication, the posterior distribution of the regression coefficients relative to the first (black curve) and second (red curve) taxon (Formula (22)). Also, this figure confirms the great precision of the inferential conclusions on regression coefficients.

Similar results are observed for all the considered Cases I–III (Section S2.1 of the SM) under typical threshold choices for the posterior means. In particular, threshold values between 0.1 and 0.2 provide a balanced control of both false negatives and false positives. Regarding the risk of false negatives, it is important to note that the posterior mean inclusion probabilities for covariates influencing only a small subset of response variables are, as expected, lower than those for covariates affecting multiple responses. However, in all scenarios, these probabilities remain sufficiently high (exceeding 0.2) to allow detection under commonly used threshold values. Moreover, comparison across the three cases reveals that these inclusion probabilities tend to decrease with increasing D, particularly when K is large.

### 4.2 Parameter Estimation, Correlation Estimation and Scalability

To assess the inferential accuracy of the proposed estimation methodology, we considered several simulation scenarios. The performance of the estimators was evaluated using their posterior mean, squared error (rMSE), and empirical coverage of 95% credible sets (CSs). Section S2.2 of the SM presents five scenarios. In the first four scenarios, the responses were generated from an EFDMReg model with D=5 and one covariate (Case IV), from a DMReg model with D=5 and one covariate (Case V), from a logistic‐normal multinomial model with D=6 and two covariates (Case VI), and from an EFDMReg model with D=5 and three covariates (Case VII), respectively. In the fifth scenario (Case VIII), we assess the accuracy of the estimation procedure of the model with D=15 and the 13 covariates selected from the initial set of 100 variables of Case III.

Table 1 refers to Case IV and reveals the high accuracy of the estimates under the EFDMReg model, with coverages very close to 0.95 for all parameters. Conversely, the other two models display worse performances, with the DMReg model showing remarkably negative behavior. Indeed, under the DMReg model, even in this simple case, the majority of regression coefficients are not well recovered, and in one case (namely, $\beta_{13}$) the negative association is missed.

**TABLE 1:** Case IV—Posterior means, rMSEs of estimators, and coverages of the 95% CS.

	DMReg			FDMReg			EFDMReg		
Parameter	Post. Mean	rMSE	Cov.	Post. Mean	rMSE	Cov.	Post. Mean	rMSE	Cov.
$\beta_{01}=-0.5$	−0.50	0.08	0.98	−0.46	0.20	0.89	−0.51	0.10	0.96
$\beta_{02}=1.5$	1.02	0.48	0.00	1.44	0.09	0.85	1.50	0.07	0.95
$\beta_{03}=2$	0.85	1.15	0.00	1.88	0.16	0.73	2.00	0.09	0.93
$\beta_{04}=3$	2.14	0.86	0.00	2.96	0.07	0.92	3.01	0.06	0.96
$\beta_{11}=1.8$	1.22	0.63	0.62	1.52	0.63	0.77	1.79	0.30	0.99
$\beta_{12}=-2.5$	−1.73	0.79	0.08	−2.20	0.39	0.70	−2.50	0.23	0.95
$\beta_{13}=-1$	0.34	1.37	0.00	−0.74	0.34	0.75	−0.99	0.20	0.95
$\beta_{14}=-2$	−1.54	0.49	0.49	−1.73	0.34	0.74	−2.00	0.21	0.94
$\alpha^{+}=50$	3.74	46.26	0.00	20.76	29.79	0.00	50.02	4.65	0.94
$p_{1}=0.25$	—	—	—	0.25	0.31	0.02	0.24	0.03	0.95
$p_{2}=0.3$	—	—	—	0.33	0.39	0.00	0.30	0.03	0.94
$p_{3}=0.2$	—	—	—	0.18	0.03	0.67	0.20	0.01	0.95
$p_{4}=0.1$	—	—	—	0.11	0.28	0.00	0.10	0.03	0.95
$p_{5}=0.15$	—	—	—	0.12	0.24	0.00	0.16	0.03	0.95
${\tilde{w}}_{1}=0.6$	—	—	—	0.86	—	—	0.59	0.34	0.95
${\tilde{w}}_{2}=0.3$	—	—	—	—	—	—	0.30	0.03	0.95
${\tilde{w}}_{3}=0.9$	—	—	—	—	—	—	0.90	0.70	0.97
${\tilde{w}}_{4}=0.4$	—	—	—	—	—	—	0.40	0.31	0.97
${\tilde{w}}_{5}=0.35$	—	—	—	—	—	—	0.35	0.20	0.96
WAIC	—	10598.28	—	—	9059.02	—	—	8705.30	—

Conversely, when the true data‐generating mechanism follows the DMReg model (Case V), the estimates are consistently accurate across all models (see Table S2 of the SM).

When the data are generated from a logistic‐normal multinomial model (Case VI), both the FDMReg and EFDMReg models exhibit clearly superior fit and greater precision in the estimation of regression coefficients compared to the DMReg model (see Figure S8 and Table S5 of the SM). Similarly, when data are generated from an EFDMReg model with three covariates (Case VII), this model achieves substantially lower WAIC values, confirming it as the best‐fitting model, and yields more accurate estimates of the regression coefficients compared to the DMReg model, as evidenced by the results reported in Table S7 and Figure S9. However, in Case VII, the remaining parameters (i.e., p's and w's) are estimated with slightly lower accuracy.

Interestingly, also in the high‐dimensional setting of Case VIII, the estimation of the EFDMReg model yields reliable (i.e., low‐bias) parameter estimates, as shown in Figure S12.

To evaluate the ability of the EFDM model to recover various correlation structures, we considered two additional scenarios (Section S2.3 of the SM), with a particular focus on positive associations that the usual DM model fails to capture. In both scenarios, models were estimated without covariates.

In the first scenario (Case IX, Section S2.3.1 of the SM), data were generated from an EFDM distribution with D=5, incorporating three positive correlations among the ten possible. The EFDM model demonstrated highly satisfactory performance in terms of model fit, parameter estimation, and accurate recovery of the specified correlations.

The second scenario (Case X, Section S2.3.2 of the SM) involves a complex data‐generating mechanism that differs substantially from our model. Specifically, the data are generated using a mixture of logistic‐normal multinomial distributions. Despite the considerable challenge posed by this setting, the EFDM model significantly outperforms the DM and FDM models in terms of WAIC and successfully identifies substantially positive correlations. However, the magnitude of these associations is not always accurately captured.

These results indicate that there may be cases where the model is not fully adequate. This limitation can be formally attributed to the EFDM model's parametrization, where the number of parameters increases linearly with D. While this characteristic reduces computational complexity, it also restricts the model's ability to represent arbitrary covariance matrices, which have quadratic dimensionality.

Finally, the study of scalability (Section S2.4 of the SM) has considered values of D up to 50 and shows good reliability of estimates once an adequate sample size is considered. As expected, the computational time increases as D increases due to the larger number of parameters and sample size, though retaining feasibility. Thanks to its simpler structure, the FDMReg model achieves a significant reduction in computational time, ranging from approximately 30% to 50%, compared to the EFDMReg model.

### 4.3 Comparison With a Zero‐Inflated Model

It is well‐known that microbiome data often display a large number of zero counts (excessive zeros). Consequently, it is valuable to assess how the EFDMReg performs compared to models specifically designed to handle this issue.

Unlike the DM model, the mixture structure of the EFDMReg model allows for the handling of excessive zeros by allocating different components of the mixture to address both zero and positive counts. An alternative approach is the development of zero‐inflated models, which introduce latent indicators to account for the presence of *structural zeros*. While this approach is interesting, it is not exempt from drawbacks [44]. One key challenge is the difficulty in untangling sampling zeros and structural zeros, which may cause biased inferences (see Section S3 of the SM for further discussion). Furthermore, it is important to note that the adoption of a zero‐inflated model complicates the evaluation of the overall impact of covariates on the response, which is typically the primary objective of such analyses.

The ZIDM model [22] is a recent and competitive zero‐inflated proposal that is obtained by modifying the Dirichlet distribution defining the DM by including point masses at zero. Covariates are linked to parameters that involve both the mean and the variance of responses, unlike the EFDMReg, which directly models the response means. This hinders the possibility of comparing the regression coefficients of the two models. Incidentally, note that care must be taken in the interpretation of the ZIDM coefficients as their signs do not generally determine the signs of the associations between covariates and count means (see the discussion in Section S3 of the SM). Standard goodness‐of‐fit criteria can not be applied, as the ZIDM does not have a closed‐form likelihood function. Therefore, we based the models' comparison on their predictive ability in terms of both zero‐counts handling (e.g., accuracy, sensitivity, specificity, positive (PPV), and negative (NPV) predictive values) and global performances measured by Kullback‐Leibler (KL) divergence of the predicted from the observed counts. We considered two main data‐generating settings, drawing data from a DM with the addition of an excess of zeros and from a ZIDM. A complete description of scenarios and results is reported in Section S3 of the SM. In essence, from the zero‐handling perspective, simulations highlight that the ZIDM regression model captures a much larger number of true zeros (sensitivity) than the EFDMReg model, as one can observe by inspecting Tables S3 and S4. However, the ZIDM shows a substantially smaller percentage of correctly predicted zeros (PPV). As for positive (i.e., nonzero) counts, the EFDMReg model succeeds in recognizing a much larger fraction of true positive counts (specificity), while still having good behavior in terms of the percentage of correctly predicted positive counts (NPV). This comparison highlights that no model completely dominates the other in zero‐handling, whereas the EFDMReg performs consistently better when positive counts are involved. As a consequence, the EFDMReg model displays an overall better performance in terms of accuracy (i.e., the ratio between the number of correct predictions and the total number of predictions). Concerning global predictive performance (KL divergence), the EFDMReg outperforms the ZIDM in all the considered situations, remarkably even when data are generated from the ZIDM itself.

**FIGURE 3:**
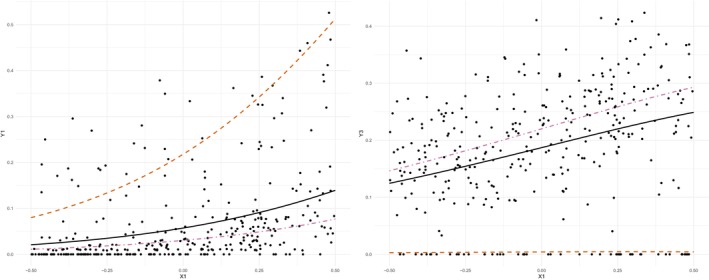
Data from DM with an excess of zeros. Scatterplot of $Y_{1}$ (left) and $Y_{3}$ (right) vs. $X_{1}$ for one randomly selected replication. Red dashed and pink dot‐dashed lines represent the regression curves for the two EFDMReg's non‐empty mixture components (namely, $\lambda_{1}$ and $\lambda_{3}$, respectively). The black solid curve represents the overall EFDMReg's fitted $\mu_{i}$.

To better understand how the EFDMReg model can handle an excessive number of zeros, we consider one randomly selected replication from the scenario with data generated from a DMReg model with an excess of zeros. More specifically, in Figure 3, we report a scatterplot of $x_{1}$ vs. $Y_{1}$ and $Y_{3}$ together with the regression curves for the two mixture components with positive estimated weights (namely, the first and the third). As for $Y_{1}$ (left panel), where no excess of zeros is present, the two component‐specific regression curves $\lambda_{1}$ and $\lambda_{3}$ capture low and, respectively, high values. Conversely, for $Y_{3}$ (right panel), the excessive number of zeros is modeled by the first component‐specific regression $\lambda_{1}$.

## 5 Application to COMBO Data

In this section, we analyze the 16S rRNA sequencing data from the COMBO dataset, originally introduced by Wu et al. [31] and subsequently examined by numerous studies (e.g., Xia et al. [24], Chen and Li [19], Shi et al. [45], Sohn and Li [46], Tang and Chen [47], Song et al. [48], and Koslovsky [22]). For a further application to a real dataset from the North American Breeding Bird Survey, see Section S6 of the SM.

The COMBO dataset, focused on the human gut microbiome, originates from a cross‐sectional study of 98 healthy volunteers. DNA extracted from stool samples was analyzed using 454/Roche pyrosequencing of 16S rRNA gene segments. Taxonomic classification identified 3068 OTUs, which were grouped into 87 genera that were present in at least one sample. The total read counts across samples exhibit substantial variation, ranging from 1242 to 14616, with an average of 9265 reads per sample and a standard deviation of 3864.

In addition to bacterial composition, the COMBO dataset includes K=120 covariates. Of these, 117 are derived from a food frequency questionnaire and provide information about micronutrient intake in participants' habitual long‐term diets. The remaining three covariates pertain to demographic and clinical characteristics: Age, gender, and BMI.

Previous studies involving these covariates have focused either on three key bacterial genera (e.g., Xia et al. [24]) or on a broader set of 13 genera (e.g., Chen and Li [19]).

**FIGURE 4:**
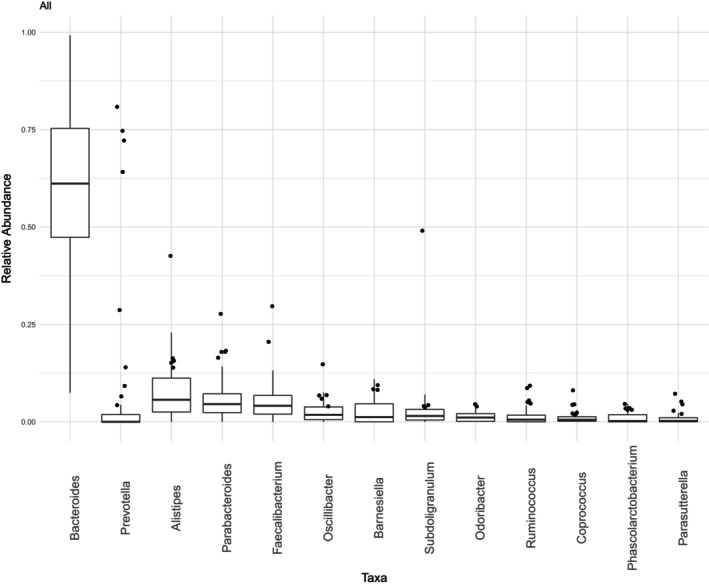
COMBO dataset. Boxplots of the relative abundances of the D=13 taxa.

Figure 4 presents boxplots of the abundances of these 13 genera, arranged in descending order of their mean values. This selection includes the three genera (i.e., *Bacteroides*, *Prevotella*, and *Ruminococcus*), which have been the subject of extensive investigation in previous studies [24, 31]. Notably, *Bacteroides* is the most abundant genus in the dataset.

In the following, we present the results of our analysis, focusing on two main aspects: Correlations among taxa (5.1) and their associations with selected covariates (5.2). First, we explore potential co‐occurrence relationships among taxa. These relationships were not highlighted in previous studies, which relied on models incapable of capturing positive correlations. Second, we investigate associations between taxa and properly selected covariates. This is particularly relevant given the large number of diet‐related covariates in the COMBO dataset, as dietary modification represents one of the most accessible therapeutic interventions for addressing compromised microbiome composition. In both analyses, we examine clinical information, specifically BMI, due to its established association with reduced bacterial diversity and altered gut microbiome composition [8].

It is important to note that while the COMBO dataset originally includes data from 98 healthy volunteers, our analysis focuses on 96 individuals. Two participants were excluded because their bacterial composition data were available only for three taxa (*Bacteroides*, *Prevotella*, and *Ruminococcus*) rather than the full 13‐dimensional compositional response.

All hyperparameter settings, HMC‐related choices (e.g., iterations, thinning, convergence assessment), as well as sensitivity analyses, can be found in Section S4.2 of the SM.

### 5.1 Correlation Study Among Taxa

A significant challenge in metagenomic studies is understanding the dependence structure among bacterial taxa. The complex interactions within the microbial community remain poorly characterized [49] In particular, while the sum constraint inherent to the simplex space typically induces negative associations among compositional elements, some bacterial taxa often exhibit positive associations, suggesting co‐occurrence relationships [30].

In this analysis, we focus on the subset of D=13 genera whose relative abundances are shown in Figure 4. To explore the correlation structure among these bacterial genera, we fit the EFDM model without including covariates. As detailed in Section 2.2, the EFDM, unlike the multinomial and DM models, accommodates positive correlations, allowing for the identification of potential co‐occurrence relationships. The EFDM correlation matrix, reported in Section S5.2 of the SM, was estimated by averaging over MCMC replications (see also Section S5.1 of the SM for remarks on the role of the number of bacterial reads within the correlation estimation process). Furthermore, the robustness of the estimates was validated through Bootstrap as detailed in Table S12 of Section S5.2 of the SM.

**FIGURE 5:**
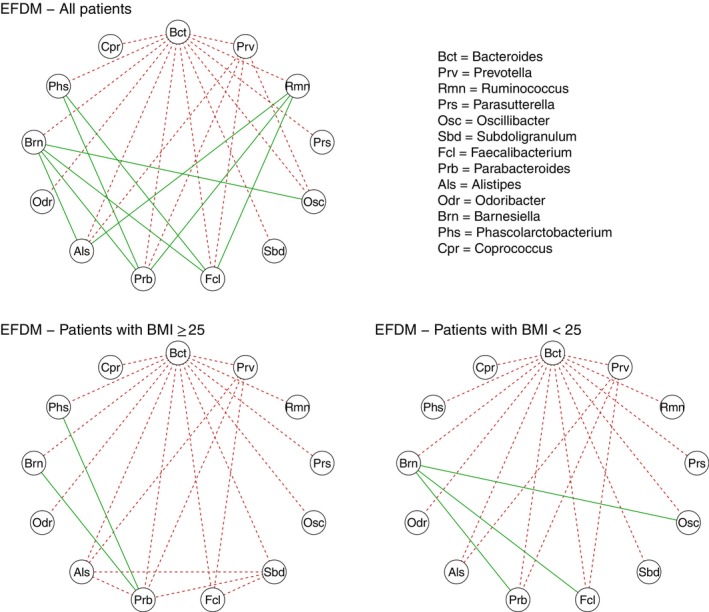
COMBO application. Correlation network among D=13 bacterial taxa. Red‐dashed lines represent negative correlations lower than −0.05, whereas green‐solid lines represent positive correlations.

The correlation estimates are visualized through a correlation network (upper panel of Figure 5). In this network, edges represent correlations between pairs of bacterial genera. Note that compositional exhibits a natural tendency toward negative dependence due to the summation constraint. In particular, the sum of the covariances of each taxon with all other taxa is necessarily negative, implying that, for instance, not all taxa can be positively associated. Therefore, we displayed all positive correlations while reporting only negative correlations below −0.05 to improve readability.

*Bacteroides* emerges as a pivotal genus, exhibiting negative correlations with all other genera. This is consistent with recent findings that identify several species within the *Bacteroides* genus as keystone taxa of the gut microbiome [50]. The correlation network further highlights *Prevotella* as a secondary key genus, showing negative correlations with five other genera, including *Bacteroides*. This result aligns with previous studies by Arumugam et al. [51] and Wu et al. [31], which identify these two genera as central to the differentiation of gut microbial enterotypes.

Notably, the EFDM model also captures clusters of positive correlations, involving seven genera. These genera are characterized by a significant number of connections, ranging from three to five edges. Among them, *Parabacteroides*, *Barnesiella*, and *Ruminococcus* stand out as central nodes within these positive correlation groups.

A deeper understanding of the correlation structure can be achieved by analyzing subgroups based on BMI, since obesity has been associated with reduced bacterial diversity and significant changes in gut microbiome composition [8]. To investigate this relationship, we divided the participants into two groups: A lean group of 63 individuals (BMI <25) and an obese group of 35 individuals (BMI ≥25). The estimated correlation matrices of the two subgroups are reported in Section S5.2 of the SM, while the corresponding correlation networks for bacterial genera are displayed in the lower panels of Figure 5 (lower right panel for lean subjects and lower left panel for obese subjects). The differences between the two groups are evident. *Bacteroides* and *Prevotella* remain pivotal in both groups, reaffirming their key roles in gut microbiome composition. However, the patterns of positive correlations distinctly differentiate lean and obese individuals. Specifically, while *Parabacteroides* retains its central role in the obese group and *Barnesiella* in the lean group, all positive correlations involving *Ruminococcus* vanish within the subgroups.

The EFDM model facilitates the exploration of both intra‐ and interclass correlations, leading to entirely new insights. Intraclass correlations, often considered measures of experimental reliability, quantify the degree to which units within the same group resemble each other [52]. Under the multinomial model, all intraclass correlations are zero, while the DM model imposes a constant value of 0.10 for all taxa in the COMBO dataset (see Section S5.3 of the SM). Conversely, the EFDM reveals a broader range of intraclass correlations, varying from 0.10 to 0.45 (see Table S15 of the SM), which underscores greater reliability across bacterial genera. In addition, the EFDM is capable of capturing various levels of overdispersion, that is, normalized variability of counts $\bar{V}(Y_{r})$ (see Section 2). For instance, *Bacteroides* exhibits a normalized variability of 0.17, whereas *Prevotella* reaches 0.45. These findings highlight the EFDM model's flexibility and its ability to account for heterogeneity in dispersion levels, unlike the multinomial or DM models.

Turning to interclass correlations, which measure the correlation between single drawings of different bacterial taxa, Table S17 of the SM reveals over 20 positive correlations identified by the EFDM. For example, strong positive correlations are observed between *Ruminococcus* and *Barnesiella* with *Oscillibacter*, *Faecalibacterium*, and *Parabacteroides*. These co‐occurrence patterns, unveiled by the EFDM, represent a significant advancement compared to competing models, which fail to identify such relationships (e.g., see Table S16 of the SM for the interclass correlations estimated under the DM model).

### 5.2 Associations Between Nutrients and Gut Microbiome

To evaluate the association between gut microbiome counts and nutritional, demographic, and clinical information, we analyze the same D=13 genera considered in Section 5.1, alongside all K=120 covariates described at the beginning of Section 5. To ensure comparability, all quantitative covariates have been standardized to operate on the same scale. The multinomial logit link function (22) requires a baseline category, which we assign to the *Coprococcus* taxon. This choice is informed by its weak association with covariates, as previously observed (e.g., Chen and Li [19]), enabling regression coefficients to approximate direct measures of association between taxa and covariates. However, it is important to note that the choice of the reference taxon does not introduce interpretative bias due to the symmetry of the EFDM model in its elements.

Given the substantial number of covariates, we employed the spike‐and‐slab VS procedure described in Section 3.3 to identify key covariates influencing the response vector (for additional details on the model's goodness‐of‐fit and posterior predictive checks, please refer to Sections S5.2.1 and S5.2.2 of the SM).

This process revealed 12 significant covariates, comprising four minerals (phosphorus, sodium, manganese, and iodine), five vitamins (vitamin B12, riboflavin B2, pyridoxine B6, choline‐phosphatidylcholine, and total choline‐no betaine), one carbohydrate (maltose), one protein (proline), and the dummy variable relative to sex. As a second step, we compared the DMReg, flexible Dirichlet‐multinomial regression (FDMReg), and EFDMReg models by re‐fitting them on the reduced design matrix, including only the intercept and the selected covariates. The posterior means and credible sets for the regression coefficients of all three models are provided in Tables S18–S20 of Section S6.1 of the SM.

The comparison of the (penalized) model fit using the WAIC metric underscores the superior performance of the EFDMReg model, which achieved a WAIC of 12,484.4. This result highlights its clear advantage over the competing models, with FDMReg also outperforming DMReg (WAIC values of 12,530.5 and 12,662.8, respectively). A significant implication of this improved fit is the enhanced precision of parameter estimates under the EFDMReg model. This is evidenced by the narrower credible sets for the regression coefficients, reflecting greater certainty in the associations detected between taxa and the selected covariates.

#### 5.2.1 Biological Findings of the EFDMReg Model

**FIGURE 6:**
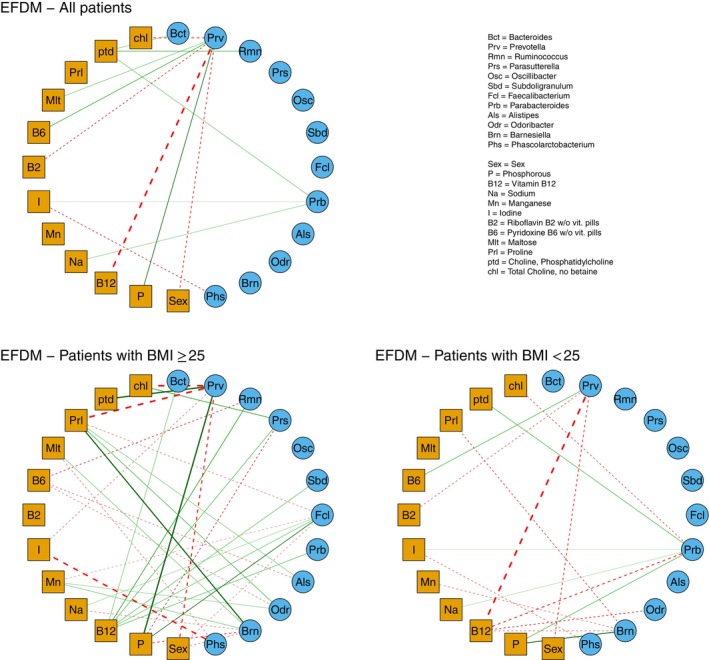
COMBO application. Bipartite graph of associations among covariates (circles) and bacterial taxa (squares). A line connecting a covariate to a taxon represents a significant positive (solid green line) or negative (dashed red line) association, determined by regression coefficients whose 90% credible sets exclude zero. The thickness of the lines is proportional to the intensity of the association.

A clear visualization of the nutrient‐taxon associations identified by the EFDMReg model (see Table S18 in the SM) is presented in the bipartite graph shown in the upper panel of Figure 6. In this graph, bacterial taxa are represented by circles, while nutrients are depicted as squares, and a line connecting a taxon to a nutrient indicates a significant association. Exponentiation of the estimates of the significant regression coefficients corresponds to estimated odds ratios ($\hat{\text{OR}}$) of each bacterial taxon to the baseline.

The results corroborate findings from Wu et al. [31], who used a pairwise approach based on the Spearman correlation coefficient. In particular, *Bacteroides* exhibited a positive association with choline‐phosphatidylcholine ($\hat{\text{OR}}=1.68$), and *Prevotella* showed a positive association with maltose ($\hat{\text{OR}}=1.77$) and a negative association with total choline‐no betaine ($\hat{\text{OR}}=0.23$). Moreover, by adopting lower confidence levels (65% to 85%) to avoid excessive filtering and thus, potentially miss diet‐bacteria interactions, additional associations were observed. Specifically, proline and sodium (whose main sources are protein‐rich foods) show a positive association with *Bacteroides* ($\hat{\text{OR}}=1.22$ and $\hat{\text{OR}}=1.14$, respectively) and a negative association with *Prevotella* ($\hat{\text{OR}}=0.58$ and $\hat{\text{OR}}=0.79$, respectively). These findings strongly support the hypothesis that *Bacteroides* and *Prevotella* characterize distinct dietary patterns [19, 24, 31] rich in proteins and sodium for *Bacteroides*, and rich in carbohydrates for *Prevotella*.

Figure 6 also highlights that *Parabacteroides* exhibits associations coherent with those of *Bacteroides*, specifically regarding choline and sodium, with a positive significant association ($\hat{\text{OR}}=1.82$ and $\hat{\text{OR}}=1.62$, respectively). This coherence aligns with the typical co‐occurrence of *Parabacteroides* within the *Bacteroides* enterotype, as both genera belong to the *Bacteroidetes* phylum [51].

Interestingly, the EFDMReg model identified further significant associations that were missed by the above‐cited models. In particular, vitamin B12, an essential nutrient for the functioning of the nervous and circulatory systems, showed a negative association with *Prevotella* ($\hat{\text{OR}}=0.02$) and a positive association with *Bacteroides* ($\hat{\text{OR}}=1.20$, at a 70% credible level). This is consistent with the protein characterization of *Bacteroides* vs. *Prevotella*, as animal‐based protein sources such as meat, liver, fish, and eggs are rich in vitamin B12. Analogous but opposite remarks hold for pyridoxine B6, which exhibited a positive association with *Prevotella* ($\hat{\text{OR}}=2.45$) and a negative association with *Bacteroides* ($\hat{\text{OR}}=0.84$, at an 80% credible level). This aligns with the nutritional profiles of these taxa, as *Prevotella* is commonly associated with diets rich in fruits, vegetables, and grains, which are primary sources of pyridoxine B6, while *Bacteroides* tends to be associated with diets less reliant on these food groups.

For a more in‐depth analysis, the EFDMReg model was applied separately to the subgroups of lean and obese individuals, defined based on BMI as in Section 5.1. The estimates of the regression coefficients and their 90% credible sets for these subgroups are reported in Tables S21 and S22 in Section S6.1 of the SM. The lower panels of Figure 6 show the significant nutrient‐taxon associations for lean (right panel) and obese (left panel) individuals. The results reveal markedly different network structures between the two groups. Obese individuals exhibit a richer and more intricate association architecture, involving almost all bacterial genera, whereas the associations for lean individuals are noticeably sparser. This suggests that obesity significantly alters the complexity and connectivity of the gut microbiome, consistent with the hypothesis that obesity plays a major role in shaping gut microbiome properties [53, 54].

#### 5.2.2 Comparison With Alternative Models

A comparison of the EFDMReg and DMReg models (Tables S18 and S20 of the SM) reveals notable differences in their capacity to detect associations between covariates and taxa. Specifically, only the EFDMReg model identifies the already mentioned positive association of choline‐phosphatidylcholine with *Bacteroides* and a new positive association of iodine with *Parabacteroides* ($\hat{\text{OR}}=1.28$), consistent with the high protein/choline diet characterizing these taxa. Conversely, the DMReg model alone identifies a negative association between Manganese and *Prevotella*. This finding, however, may be less reliable, as manganese is primarily sourced from cereals, nuts, and legumes; thus, the negative association aligns poorly with the dietary patterns typically associated with Prevotella. These observations suggest a superior ability of EFDMReg to identify biologically plausible associations.

The comparison between the EFDMReg and the ZIDM models offers additional insights, particularly given the sparsity of the COMBO dataset, where the proportion of zero values ranges from 0% to 65%, with an average of 20% (see Table S9 of the SM). Results reported in Section S6.2 of the SM show that, in terms of zero‐handling, the ZIDM regression model demonstrates greater sensitivity and NPV, while EFDMReg exhibits higher specificity and PPV, but the accuracy measure favors EFDMReg as the superior model. Moreover, when evaluating the overall predictive performance using the KL divergence, the EFDMReg model substantially outperforms the ZIDM. Finally, from the perspective of significant associations between taxa and covariates, EFDMReg provides broader and more nuanced findings. While the ZIDM model detects associations for only two taxa—Bacteroides (2 associations) and Prevotella (11 associations)—EFDMReg uncovers a richer network of relationships (see Figure 6) in closer alignment with established microbiome literature [19]. This highlights the enhanced capacity of EFDMReg to capture biologically meaningful patterns in the data.

## 6 Concluding Remarks

The EFDMReg model represents a significant advancement over the standard DMReg model and is, in many respects, advantageous compared to state‐of‐the‐art models. Its enhanced flexibility allows it to capture critical features of microbiome data, such as inter‐ and intraclass correlations and taxon‐specific overdispersion. Both simulation and application studies highlight that these capabilities not only improve fitting and predictive performance but also lead to more accurate parameter estimation and improved detection of taxa‐covariate associations.

Importantly, in datasets characterized by excessive zeros, the specific mixture structure of the EFDMReg model enables competitive zero handling compared to models explicitly incorporating a structural zero component, while simultaneously offering more reliable association detection and straightforward interpretability.

Future work will focus on extending the EFDMReg model to further enhance its flexibility, fitting capability, and ability to capture intricate data patterns. One promising direction is the inclusion of random effects, which would enable the model to accommodate longitudinally measured responses Additionally, the model's computational efficiency could be further optimized to handle scenarios with a large number of taxa D.

Finally, the model's potential applications extend far beyond microbiome research. The ability to handle complex count data makes it suitable for various fields, including text analysis and social surveys. Exploring its applicability in these areas could open new avenues for research and broaden its impact.

## Conflicts of Interest

The authors declare no conflicts of interest.

## Supporting information




**Data S1**: Supporting Information.

## Data Availability

The data that support the findings of this study are available from the corresponding author upon reasonable request.

## References

[1] K. Amato , “An Introduction to Microbiome Analysis for Human Biology Applications,” American Journal of Human Biology 29 (2017): e22931, 10.1002/ajhb.22931. 27762069

[2] X. C. Morgan and C. Huttenhower , “Human Microbiome Analysis,” PLoS Computational Biology 8, no. 12 (2012): e1002808, 10.1371/journal.pcbi.1002808. 23300406 PMC3531975

[3] F. Bäckhed , R. E. Ley , J. L. Sonnenburg , D. A. Peterson , and J. I. Gordon , “Host‐Bacterial Mutualism in the Human Intestine,” Science 307, no. 5717 (2005): 1915–1920, 10.1126/science.1104816. 15790844

[4] B. Zhu , X. Wang , and L. Li , “Human Gut Microbiome: The Second Genome of Human Body,” Protein & Cell 1, no. 8 (2010): 718–725, 10.1007/s13238-010-0093-z. 21203913 PMC4875195

[5] E. A. Grice and J. A. Segre , “The Human Microbiome: Our Second Genome,” Annual Review of Genomics and Human Genetics 13 (2012): 151–170, 10.1146/annurev-genom-090711-163814. PMC3518434 22703178

[6] R. A. Koeth , Z. Wang , B. S. Levison , et al., “Intestinal Microbiota Metabolism of l‐Carnitine, a Nutrient in Red Meat, Promotes Atherosclerosis,” Nature Medicine 19, no. 5 (2013): 576–585, 10.1038/nm.3145. PMC3650111 23563705

[7] J. Qin , Y. Li , Z. Cai , et al., “A Metagenome‐Wide Association Study of Gut Microbiota in Type 2 Diabetes,” Nature 490, no. 7418 (2012): 55–60, 10.1038/nature11450. 23023125

[8] P. J. Turnbaugh , M. Hamady , T. Yatsunenko , et al., “A Core Gut Microbiome in Obese and Lean Twins,” Nature 457, no. 7228 (2009): 480–484, 10.1038/nature07540. 19043404 PMC2677729

[9] J. Libertucci and V. B. Young , “The Role of the Microbiota in Infectious Diseases,” Nature Microbiology 4 (2019): 35–45, 10.1038/s41564-018-0278-4. 30546094

[10] J. Lloyd‐Price , C. Arze , A. N. Ananthakrishnan , et al., “Multi‐Omics of the Gut Microbial Ecosystem in Inflammatory Bowel Diseases,” Nature 569, no. 7758 (2019): 655–662, 10.1038/s41586-019-1237-9. 31142855 PMC6650278

[11] W. R. Streit and R. A. Schmitz , “Metagenomics ‐ the Key to the Uncultured Microbes,” Current Opinion in Microbiology 7, no. 5 (2004): 492–498, 10.1016/j.mib.2004.08.002. 15451504

[12] P. D. Schloss , “The Effects of Alignment Quality, Distance Calculation Method, Sequence Filtering, and Region on the Analysis of 16S rRNA Gene‐Based Studies,” PLoS Computational Biology 6, no. 7 (2010): 19, 10.1371/journal.pcbi.1000844. PMC2900292 20628621

[13] B. P. Youmans , N. J. Ajami , Z. D. Jiang , et al., “Characterization of the Human Gut Microbiome During Travelers' Diarrhea,” Gut Microbes 6, no. 2 (2015): 110–119, 10.1080/19490976.2015.1019693. 25695334 PMC4615231

[14] M. Hamady , C. Lozupone , and R. Knight , “Fast UniFrac: Facilitating High‐Throughput Phylogenetic Analyses of Microbial Communities Including Analysis of Pyrosequencing and PhyloChip Data,” ISME Journal 4, no. 1 (2010): 17–27, 10.1038/ismej.2009.97. 19710709 PMC2797552

[15] X. Zhang , H. Mallick , Z. Tang , et al., “Negative Binomial Mixed Models for Analyzing Microbiome Count Data,” BMC Bioinformatics 18, no. 4 (2017): 1–10, 10.1186/s12859-016-1441-7. 28049409 PMC5209949

[16] K. Shuler , M. Sison‐Mangus , and J. Lee , “Bayesian Sparse Multivariate Regression With Asymmetric Nonlocal Priors for Microbiome Data Analysis,” Bayesian Analysis 15, no. 2 (2020): 559–578, 10.1214/19-BA1164.

[17] V. Jonsson , T. Österlund , O. Nerman , and E. Kristiansson , “Modelling of Zero‐Inflation Improves Inference of Metagenomic Gene Count Data,” Statistical Methods in Medical Research 28, no. 12 (2019): 3712–3728, 10.1177/0962280218811354. 30474490

[18] J. E. Mosimann , “On the Compound Multinomial Distribution, the Multivariate β‐ Distribution, and Correlations Among Proportions,” Biometrika 49, no. 1/2 (1962): 65–82, 10.2307/2333468.

[19] J. Chen and H. Li , “Variable Selection for Sparse Dirichlet‐Multinomial Regression With an Application to Microbiome Data Analysis,” Annals of Applied Statistics 111, no. 1 (2013): 418–442, 10.1214/12-AOAS592. PMC3846354 24312162

[20] W. D. Wadsworth , R. Argiento , M. Guindani , J. Galloway‐Pena , S. A. Shelbourne , and M. Vannucci , “An Integrative Bayesian Dirichlet‐Multinomial Regression Model for the Analysis of Taxonomic Abundances in Microbiome Data,” BMC Bioinformatics 18, no. 1 (2017): 1–12, 10.1186/s12859-017-1516-0. 28178947 PMC5299727

[21] M. Pedone , A. Amedei , and F. Stingo , “Subject‐Specific Dirichlet‐Multinomial Regression for Multi‐District Microbiota Data Analysis,” Annals of Applied Statistics 17, no. 1 (2023): 539–559, 10.1214/22-AOAS1641.

[22] M. Koslovsky , “A Bayesian Zero‐Inflated Dirichlet‐Multinomial Regression Model for Multivariate Compositional Count Data,” Biometrics 79 (2023): 3239–3251, 10.1111/biom.13853. 36896642

[23] J. Harrison , W. Calder , V. Shastry , and C. Buerkle , “Dirichlet‐Multinomial Modelling Outperforms Alternatives for Analysis of Microbiome and Other Ecological Count Data,” Molecular Ecology Resources 20 (2020): 481–497, 10.1111/1755-0998.13128. 31872949

[24] F. Xia , J. Chen , W. K. Fung , and H. Li , “A Logistic Normal Multinomial Regression Model for Microbiome Compositional Data Analysis,” Biometrics 69, no. 4 (2013): 1053–1063, 10.1111/biom.12079. 24128059

[25] Y. Zhang , H. Zhou , J. Zhou , and W. Sun , “Regression Models for Multivariate Count Data,” Journal of Computational and Graphical Statistics 26, no. 1 (2017): 1–13, 10.1080/10618600.2016.1154063. 28348500 PMC5365157

[26] N. S. Grantham , Y. Guan , B. J. Reich , E. Borer , and K. Gross , “MIMIX: A Bayesian Mixed‐Effects Model for Microbiome Data From Designed Experiments,” Journal of the American Statistical Association 115, no. 530 (2020): 599–609, 10.1080/01621459.2019.1626242.

[27] B. Ren , S. Bacallado , S. Favaro , T. Vatanen , C. Huttenhower , and L. Trippa , “Bayesian Mixed Effects Models for Zero‐Inflated Compositions in Microbiome Data Analysis,” Annals of Applied Statistics 14, no. 1 (2020): 494–517, 10.1214/19-AOAS1295.

[28] R. Connor and J. E. Mosimann , “Concepts of Independence for Proportions With a Generalization of the Dirichlet Distribution,” Journal of the American Statistical Association 64, no. 325 (1969): 194–206, 10.2307/2283728.

[29] J. Aitchison , The Statistical Analysis of Compositional Data, 2nd ed. (Blackburn Press, 2003).

[30] K. Faust , J. Sathirapongsasuti , J. Izard , et al., “Microbial Co‐Occurrence Relationships in the Human Microbiome,” PLoS Computational Biology 8 (2012): e1002606, 10.1371/journal.pcbi.1002606. 22807668 PMC3395616

[31] G. D. Wu , J. Chen , C. Hoffmann , et al., “Linking Long‐Term Dietary Patterns With Gut Microbial Enterotypes,” Science 334 (2011): 105–109, 10.1126/science.1208344. 21885731 PMC3368382

[32] M. Schervish , Theory of Statistics (Springer New York, 1995).

[33] A. Ongaro , S. Migliorati , and R. Ascari , “A New Mixture Model on the Simplex,” Statistics and Computing 30 (2020): 749–770, 10.1007/s11222-019-09920-x.

[34] A. Ongaro and S. Migliorati , “A Generalization of the Dirichlet Distribution,” Journal of Multivariate Analysis 114, no. 1 (2013): 412–426, 10.1016/j.jmva.2012.07.007.

[35] S. Migliorati , A. Ongaro , and G. S. Monti , “A Structured Dirichlet Mixture Model for Compositional Data: Inferential and Applicative Issues,” Statistics and Computing 27, no. 4 (2017): 963–983, 10.1007/s11222-016-9665-y.

[36] R. Ascari and S. Migliorati , “A New Regression Model for Overdispersed Binomial Data Accounting for Outliers and an Excess of Zeros,” Statistics in Medicine 40, no. 17 (2021): 3895–3914, 10.1002/sim.9005. 33960503 PMC8360060

[37] R. M. Neal , “An Improved Acceptance Procedure for the Hybrid Monte Carlo Algorithm,” Journal of Computational Physics 111, no. 1 (1994): 194–203, 10.1006/jcph.1994.1054.

[38] A. Gelman , J. B. Carlin , H. S. Stern , D. B. Dunson , A. Vehtari , and D. B. Rubin , Bayesian Data Analysis, 3rd ed. (CRC Press, 2014).

[39] B. Carpenter , A. Gelman , M. D. Hoffman , et al., “Stan: A Probabilistic Programming Language,” Journal of Statistical Software 76, no. 1 (2017): 1–32. 36568334 10.18637/jss.v076.i01 PMC9788645

[40] A. Vehtari , A. Gelman , and J. Gabry , “Practical Bayesian Model Evaluation Using Leave‐One‐Out Cross‐Validation and WAIC,” Statistics and Computing 27, no. 5 (2017): 1413–1432, 10.1007/s11222-016-9696-4.

[41] S. Watanabe , “A Widely Applicable Bayesian Information Criterion,” Journal of Machine Learning Research 14, no. 1 (2013): 867–897.

[42] T. Mitchell and J. Beauchamp , “Bayesian Variable Selection in Linear Regression,” Journal of the American Statistical Society 83 (1988): 1023–1036, 10.1080/01621459.1988.10478694.

[43] P. Brown , M. Vannucci , and T. Fearn , “Multivariate Bayesian Variable Selection and Prediction,” Journal of the Royal Statistical Society B 60, no. 3 (1998): 627–641, 10.1111/1467-9868.00144.

[44] J. Silverman , K. Roche , S. Mukherjee , and L. David , “Naught All Zeros in Sequence Count Data Are the Same,” Computational and Structural Biotechnology Journal 18 (2020): 2789–2798, 10.3389/fgene.2020.602594. 33101615 PMC7568192

[45] P. Shi , A. Zhang , and H. Li , “Regression Analysis for Microbiome Compositional Data,” Annals of Applied Statistics 10, no. 2 (2016): 1019–1040, 10.1214/16-AOAS928.

[46] M. B. Sohn and H. Li , “Compositional Mediation Analysis for Microbiome Studies,” Annals of Applied Statistics 13, no. 1 (2019): 661–681, 10.1214/18-AOAS1210.

[47] Z. Z. Tang and G. Chen , “Zero‐Inflated Generalized Dirichlet Multinomial Regression Model for Microbiome Compositional Data Analysis,” Biostatistics 20, no. 4 (2019): 698–713. 29939212 10.1093/biostatistics/kxy025 PMC7410344

[48] Y. Song , H. Zhao , and T. Wang , “An Adaptive Independence Test for Microbiome Community Data,” Biometrics 76, no. 2 (2020): 661–681, 10.1111/biom.13154. 31538660

[49] K. Coyte , J. Shluter , and K. Foster , “The Ecology of the Microbiome: Networks, Competition, and Stability,” Science 350, no. 6261 (2015): 663–666, 10.1126/science.aad2602. 26542567

[50] C. Fisher and P. Metha , “Identifying Keystone Species in the Human Gut Microbiome From Metagenomic Timeseries Using Sparse Linear Regression,” PLoS One 7, no. 9 (2014): e102451, 10.1371/journal.pone.0102451. PMC4108331 25054627

[51] M. Arumugam , J. Raes , E. Pelletier , et al., “Enterotypes of the Human Gut Microbiome,” Nature 473, no. 7346 (2011): 174–180, 10.1038/nature09944. 21508958 PMC3728647

[52] D. Liljequist , B. Elfving , and K. Skavberg Roaldsen , “Intraclass Correlation ‐ A Discussion and Demonstration of Basic Features,” PLoS One 14 (2019): e0219854, 10.1371/journal.pone.0219854. 31329615 PMC6645485

[53] J. Zhang and W. Lin , “Scalable Estimation and Regularization for the Logistic Normal Multinomial Model,” Biometrics 75 (2019): 1098–1108, 10.1111/biom.13071. 31009062

[54] Y. Cao , W. Lin , and H. Li , “Large Covariance Estimation for Compositional Data via Compositional‐Adjusted Thresholding,” Journal of the American Statistical Association 114, no. 526 (2019): 759–772, 10.1080/01621459.2018.1442340.

