# Supplementary material for: A New Dirichlet‐Multinomial Mixture Regression Model for the Analysis of Microbiome Data
Source: Stat Med. 2025 Aug 7;44(18-19):e70220. doi: 10.1002/sim.70220 (PMC12330782; doi:10.1002/sim.70220)
Supplement: Supplementary file 1 — Data S1: Supporting Information. [file SIM-44-0-s001.pdf]

Supplementary Material for the paper "A new Dirichlet-multinomial mixture regression model for the analysis of microbiome data"

by R. Ascari, S. Migliorati, and A. Ongaro

June 25, 2025

Contents

1 Supplementary results relative to the extended flexible Dirichlet-multinomial (EFDM) distribution (Section 2.2 of the paper) 3

1.1 Multimodality . . . 3

1.2 Proof of Proposition 1: identifiability of the EFDM distribution . 3

1.3 Moments of the EFD distribution . . . 4

1.4 Covariance matrix of the EFDM distribution . . . 5

2 Simulation study 1: EFDMReg variable selection (Section 4.1 of the paper), parameter estimation, correlation estimation, and scalability (Section 4.2 of the paper) 6

2.1 Variable selection . . . 6

2.1.1 Case I ($D = 3$ taxa and $K = 9$ covariates) and case II ($D = 3$ taxa and $K = 20$ covariates) . . . 6

2.1.2 Case III ($D = 15$ taxa and $K = 100$ covariates) . . . 8

2.2 Parameter estimation . . . 10

2.2.1 Cases IV and V: data from an extended flexible Dirichlet-multinomial regression (EFDMReg) and from a Dirichlet-multinomial regression (DMReg) with one covariate . . . 10

2.2.2 Case VI: data from a logistic-normal multinomial model with two covariates . . . 15

2.2.3 Case VII: data from an EFDMReg with three covariates . 16

2.2.4 Case VIII: estimation of the model with the 13 covariates selected in case III . . . 19

2.3 Correlation estimation . . . 21

2.3.1 Case IX: data from EFDM model with large positive correlations . . . 22

2.3.2 Case X: data from a mixture of logistic-normal multinomial 24

2.4 Scalability . . . 27

3 Simulation study 2: Comparison of EFDMReg and ZIDM models (Section 4.3 of the paper) 30

3.1 Data from DM with an excess of zeros in two elements of the response . . . 33

3.2 Data from ZIDM . . . 34

4 Computational aspects 38

4.1 Instructions for running the EFDMReg Model . . . 38

4.2 Hyperparameter choices, HMC implementation, computational time, and sensitivity analysis (Section 5 of the paper) . . . 39

5 COMBO application (Section 5 of the paper) 45

5.1 Correlation estimation (Section 5.1 of the paper) . . . 45

5.1.1 Correlation behavior for increasing bacterial reads . . . 45

5.1.2 EFDM fitted correlation matrix . . . 45

5.1.3 Intra- and interclass correlation coefficients with $D = 13$. 51

5.2 Estimation of regression models (Section 5.2 of the paper) . . . 54

5.2.1 Estimation of EFDMReg, FDMReg, and DMReg models . 54

5.2.2 Posterior predictive checks . . . 60

5.2.3 Estimation of ZIDM model . . . 63

6 Birds Application 67

1 Supplementary results relative to the EFDM distribution (Section 2.2 of the paper)

1.1 Multimodality

The mixture structure of the EFDM model, among other properties, allows for up to D possible modes.

In Figure S1, the probability mass function (p.m.f.) of the Dirichlet-multinomial (DM) and EFDM distributions are compared employing a discrete ternary diagram, which is an equilateral triangle representing the three elements of $\mathbf{y}$ in a completely symmetric fashion. Here ($D = 3$) a clear bimodality of the EFDM can be noted. Indeed, even if the EFDM considers three nonempty components, its p.m.f. shows only two distinct modes due to two small (with respect to α^+) τ_r values that place the second and the third clusters close to each other.

Figure S1: Probability mass function of the DM (left panel) and the EFDM (right panel) distributions with $n = 15, \boldsymbol{\alpha} = (20, 100, 30)^\intercal, \boldsymbol{\tau} = (80, 10, 10)^\intercal$, and $\mathbf{p} = (0.5, 0.25, 0.25)^\intercal$. The blue squares represent the component-specific barycenters $\boldsymbol{\lambda}_1$, $\boldsymbol{\lambda}_2$, and $\boldsymbol{\lambda}_3$.

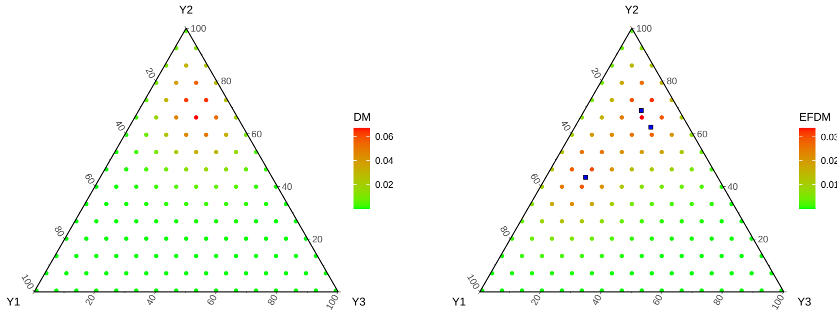

1.2 Proof of Proposition 1: identifiability of the EFDM distribution

Clearly if $\boldsymbol{\theta} = \boldsymbol{\theta}'$, then $\mathbf{Y} \sim \mathbf{Y}'$. Let us show the converse, and therefore suppose $\mathbf{Y} \sim \mathbf{Y}'$. By definition, $\mathbf{Y}$ admits the representation $\mathbf{Y}|\boldsymbol{\Pi} \sim \text{Mult}(n, \boldsymbol{\Pi})$ with $\boldsymbol{\Pi} \sim EFD(\boldsymbol{\theta})$, and similarly for $\mathbf{Y}'$ it holds $\mathbf{Y}'|\boldsymbol{\Pi}' \sim \text{Mult}(n, \boldsymbol{\Pi}')$ with $\boldsymbol{\Pi}' \sim EFD(\boldsymbol{\theta}')$. For a non-negative integer m, let ${}_{[m]}x$ be the falling factorial ${}_{[m]}x = x(x-1)\dots(x-m+1)$ with ${}_{[0]}x = 1$. The joint factorial moments of $\mathbf{Y}$, defined as $\mathbb{E}\left[\prod_{r=1}^{D} {}_{[m_r]}Y_r\right]$, exist for arbitrary non-negative integers m_js as the

random vector (r.v.) $\mathbf{Y}$ is bounded. Furthermore, the factorial joint moments of a multinomial distribution $\mathbf{Z} \sim \text{Mult}(n, \boldsymbol{\pi})$ are given by

$$\mathbb{E}\left[\prod_{r=1}^{D} {}_{[m_r]}Z_r\right] = n^{m^+} \prod_{r=1}^{D} \pi_r^{m_r},$$

where $m^+ = \sum_{r=1}^{D} m_r$ (see, e.g., [3]). It follows that

$$\mathbb{E}\left[\prod_{r=1}^{D} {}_{[m_r]}Y_r\right] = n^{m^+} \mathbb{E}\left[\prod_{r=1}^{D} \Pi_r^{m_r}\right] \tag{1}$$

and, likewise,

$$\mathbb{E}\left[\prod_{r=1}^{D} {}_{[m_r]}Y_r'\right] = n^{m^+} \mathbb{E}\left[\prod_{r=1}^{D} {\Pi'_r}^{m_r}\right]. \tag{2}$$

Expressions (1) and (2) are equal as $\mathbf{Y} \sim \mathbf{Y}'$. It follows that the joint moments of any order of $\boldsymbol{\Pi}$ and $\boldsymbol{\Pi}'$ coincide. Since $\boldsymbol{\Pi}$ and $\boldsymbol{\Pi}'$ are bounded vectors, then $\boldsymbol{\Pi} \sim \boldsymbol{\Pi}'$, and identifiability of the EFD distribution implies that $\boldsymbol{\theta} = \boldsymbol{\theta}'$.

1.3 Moments of the EFD distribution

Let $\boldsymbol{\Pi}$ be a r.v. distributed according to an EFD$(\boldsymbol{\alpha}, \boldsymbol{\tau}, \mathbf{p})$ distribution. Then, for $r, l \in \{1, \dots D\}$, $r \neq l$,

$$\mathbb{E}\left[\Pi_r\right] = \alpha_r k_1 + \tau_r \frac{p_r}{\alpha^+ + \tau_r}, \tag{3}$$

$$\begin{aligned} \text{Var}\left(\Pi_r\right) = \alpha_r^2 (k_2 - k_1^2) + \frac{p_r \tau_r (2\alpha_r + \tau_r + 1)}{(\alpha^+ + \tau_r)(\alpha^+ + \tau_r + 1)} + \\ + \alpha_r k_2 - \frac{p_r^2 \tau_r^2}{(\alpha^+ + \tau_r)^2} - k_1 \frac{2\alpha_r p_r \tau_r}{\alpha^+ + \tau_r}, \end{aligned} \tag{4}$$

$$\begin{aligned} \text{Cov}\left(\Pi_r, \Pi_l\right) = \alpha_i \alpha_l (k_2 - k_1^2) - \frac{p_r p_l \tau_r \tau_l}{(\alpha^+ + \tau_r)(\alpha^+ + \tau_l)} + \\ + \frac{\alpha_r p_l \tau_l}{\alpha^+ + \tau_l} \left(\frac{1}{\alpha^+ + \tau_l + 1} - k_1\right) + \frac{\alpha_l p_r \tau_r}{\alpha^+ + \tau_r} \left(\frac{1}{\alpha^+ + \tau_r + 1} - k_1\right), \end{aligned} \tag{5}$$

where k_1 and k_2 are defined as

$$k_1 = \sum_{r=1}^{D} \frac{p_r}{\phi_r}, \quad k_2 = \sum_{r=1}^{D} \frac{p_r}{\phi_r(\phi_r + 1)}, \text{ with } \phi_r = \alpha^+ + \tau_r \text{ and } \alpha^+ = \sum_{r=1}^{D} \alpha_r. \tag{6}$$

For more details about the EFD distribution, see [4].

1.4 Covariance matrix of the EFDM distribution

Formula (14) of the main paper gives the covariance matrix of the EFDM, i.e.:

$$\mathbb{V}_{\text{EFDM}}(\mathbf{Y}) = n\mathbf{M}(\boldsymbol{\mu})\left[1+(n-1)\left(1-\frac{k_2}{k_1^2}\right)\right]+n(n-1)\left[\frac{k_2}{k_1^2}\mathbf{M}(\mathbf{d})+\boldsymbol{\Psi}+\mathbf{W}\right],$$

where

$$k_1 = \sum_{r=1}^{D}\frac{p_r}{\phi_r}, \quad k_2 = \sum_{r=1}^{D}\frac{p_r}{\phi_r(\phi_r+1)}, \quad \text{with} \quad \phi_r = \alpha^+ + \tau_r$$

and

$$\boldsymbol{\Psi} = \text{Diag}(\boldsymbol{\psi}), \qquad \mathbf{W} = \boldsymbol{\alpha}\boldsymbol{\gamma}^\intercal + \boldsymbol{\gamma}\boldsymbol{\alpha}^\intercal,$$

with $\mathbf{d}$, $\boldsymbol{\psi}$, and $\boldsymbol{\gamma}$ representing vectors with elements $d_r = \frac{p_r\tau_r}{\phi_r}$, $\psi_r = \alpha_r\left(\frac{k_2}{k_1} - k_1 + k_2\right) - \frac{\alpha^+ d_r}{\phi_r+1}$, and $\gamma_r = d_r\left(\frac{1}{\phi_r+1} - \frac{k_2}{k_1}\right)$, respectively.

A comparison with the covariance matrix of the DM given by (7), i.e.

$$\mathbb{V}_{\text{DM}}(\mathbf{Y}) = n\mathbf{M}(\boldsymbol{\mu})\left[1+\frac{n-1}{\alpha^+ + 1}\right]$$

highlights two main differences, namely, the coefficient multiplying the matrix $\mathbf{M}(\boldsymbol{\mu})$ and the newly introduced matrices $\mathbf{M}(\mathbf{d})$, $\boldsymbol{\Psi}$, and $\mathbf{W}$. Concerning the coefficient, we underline that the positive coefficient $(\alpha^+ + 1)^{-1}$ of the DM is replaced by $\left(1 - k_2/k_1^2\right)$ in the EFDM. The latter can take on both positive and negative values, depending on the variability of the τ_rs, as the following expression holds:

$$k_1^2 - k_2 = \mathbb{E}\left[\frac{1}{(\alpha^+ + T)^2(\alpha^+ + T + 1)}\right] - \text{Var}\left(\frac{1}{\alpha^+ + T}\right),$$

where T is a discrete random variable taking values τ_r with probability p_r, $(r = 1, \ldots, D)$.[4] Thus, the term involving the matrix $\mathbf{M}(\boldsymbol{\mu})$ in the EFDM model can produce positive dependencies. For the new matrices, $\mathbf{M}(\mathbf{d})$ only entails a negative contribution to the dependencies. The matrix $\mathbf{W}$ may contain both positive and negative values since the sign of each off-diagonal term depends on the γ_rs. The latter assume positive values when τ_rs are small and negative values for large τ_rs. Interestingly, the γ_rs are all equal to zero, leading to a null matrix $\mathbf{W}$, iff the τ_rs are all equal.

2 Simulation study 1: EFDMReg variable selection (Section 4.1 of the paper), parameter estimation, correlation estimation, and scalability (Section 4.2 of the paper)

This section aims to evaluate the validity of the variable selection method described in Section 3.3 of the main paper, the performance of the proposed parameter estimation procedure for the EFDMReg model (Section 3.2 of the main paper) and correlation estimation, and the scalability of the EFDMReg model. To this end, we considered several scenarios with increasing values of the number of covariates K and the number of taxa D.

2.1 Variable selection

Concerning variable selection, we set regression coefficients so that some covariates do not affect any taxa, some other covariates only a subset of taxa, and the remaining ones all taxa. In the following, we report a detailed description of three representative cases with $K = 9$ covariates (case I), $K = 20$ covariates (case II) and $K = 100$ covariates (case III) respectively but similar results were obtained in different settings.

2.1.1 Case I ($D = 3$ taxa and $K = 9$ covariates) and case II ($D = 3$ taxa and $K = 20$ covariates)

For cases (I) and (II) we implemented simulation studies composed of $B = 300$ replications. For each replication, we generated $N = 750$ samples and assumed to read $D = 3$ bacterial taxa. For sample i, we read $n_i \sim Po(50)$ bacteria and collected K additional covariates $x_1, \ldots, x_K$. Assuming standardized covariates, we generated them independently from $N(0, 1)$. Response vectors were generated according to an EFDMReg model.

In case I, we set the following parameters:

$$\boldsymbol{\beta} = [\boldsymbol{\beta}_0^* \mid \boldsymbol{\beta}_1^* \mid \boldsymbol{\beta}_2^* \mid \cdots \mid \boldsymbol{\beta}_9^*] = \begin{bmatrix} -4 & 0 & -3 & -3 & 2.7 & 0 & 3 & 0 & 0 & 0 \\ 1.5 & 0 & -2 & 0.5 & -3 & 0 & 2.5 & 0 & 0 & 0 \\ 0 & 0 & 0 & 0 & 0 & 0 & 0 & 0 & 0 & 0 \end{bmatrix},$$

where $\boldsymbol{\beta}_k^*$ is the vector of regression coefficients associated with the k-th covariate, as specified in Section 4.3 of the main paper, $\alpha^+ = 50$, $\mathbf{p} = (0.25, 0.25, 0.5)^\intercal$, and $\tilde{\mathbf{w}} = (0.6, 0.8, 0.7)^\intercal$. Within the setting of $\boldsymbol{\beta}$, we include three covariates (the second, the fourth, and the sixth) having an impact on both the equations defining the mean vector (see Formula (22) of the main paper) and one covariate (the third) affecting mainly the first equation.

Then, for each replication, we fitted the EFDMReg model with the spike and slab priors described in Section 3.3 of the main paper. Figure S2 shows

the posterior (simulated) density for each θ_k (i.e., the probability that the k-th covariate is included in the model) for a randomly selected replication.

Figure S2: Case I - Posterior distribution of the probability of inclusion of all the $K = 9$ covariates in a randomly selected replication.

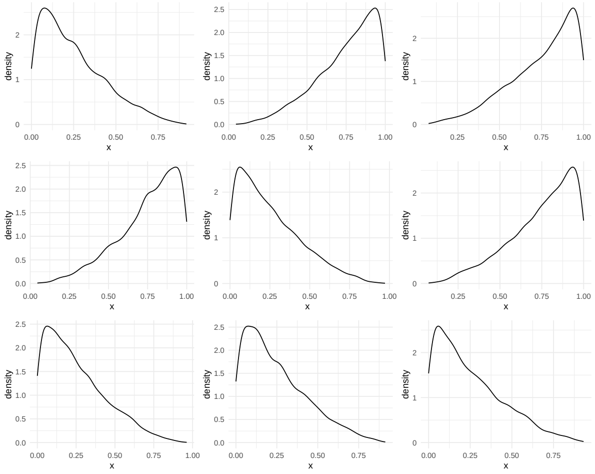

To summarize results for all the $B = 300$ replications, Figure S3 reports the boxplots of the B posterior means of the probability of inclusion θ_k's for each covariate.

Figure S3: Case I - Boxplot of the posterior mean of the probability of inclusion for each covariate across replications.

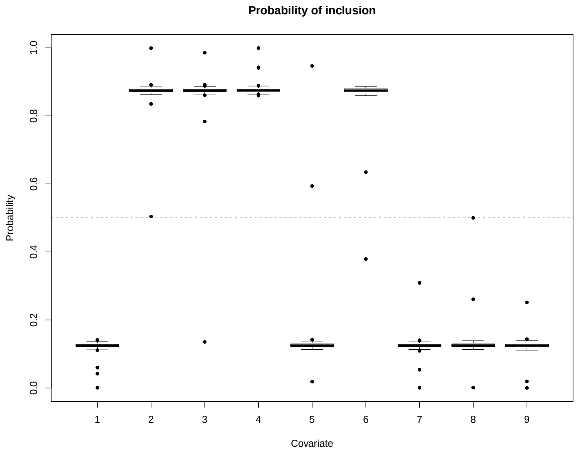

Both figures highlight the ability to recover the relevant covariates. In particular, the boxplots in Figure S3 show very few cases of incorrect selection,

thus implying values of sensitivity and specificity close to 1.

Moreover, Figure S4 shows, for a randomly selected replication, the posterior distribution of the regression coefficients relative to the first (black curve) and second (red curve) taxon (Formula (22) of the main paper). Also this figure confirms the great precision of the inferential conclusions on regression coefficients.

Figure S4: Case I - Posterior distribution of regression coefficients from a randomly selected MC replication. Black and red curves refer to the first and second elements of the mean vector, respectively.

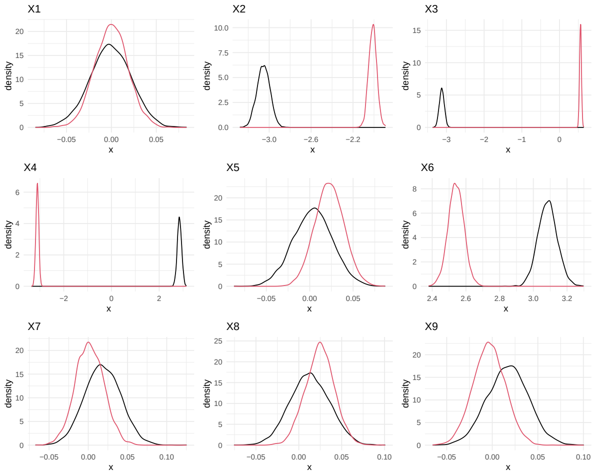

Case II refers to a scenario characterized by 20 covariates, seven of which are relevant. Among these important covariates, five (i.e., the second, the fourth, the sixth, the tenth, and the twelfth) affect both the regression equations, whereas the remaining two (i.e., the first and the twentieth) affect only one equation. Inspecting Figure S5, it is possible to note that the VS procedure correctly recognizes the variables to be selected, detecting the variables affecting both the regression equations with higher confidence. Moreover, Figure S6 validates the reliability of the estimation procedure of regression coefficients.

2.1.2 Case III ($D = 15$ taxa and $K = 100$ covariates)

Lastly, we considered a third scenario (Case III), in which both D and K are increased. More precisely, we set $D = 15$ and $K = 100$ to mimic the settings of the COMBO application. In this scenario, we made the following assumptions:

- two covariates (specifically, the first and the second) exhibit a non-zero association across all regression equations with randomly selected regression coefficients in the set $\{\pm 1.5, \pm 1.8\}$;
- eight covariates (i.e., third to tenth) have a medium effect on (different)

Figure S5: Case II - Boxplot of the posterior mean of the probability of inclusion for each covariate across replications.

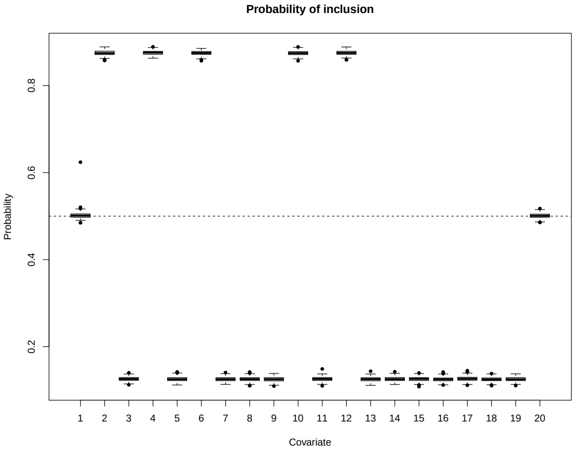

Figure S6: Case II - Posterior distribution of regression coefficients from a randomly selected MC replication. Black and red curves refer to the first and second elements of the mean vector, respectively.

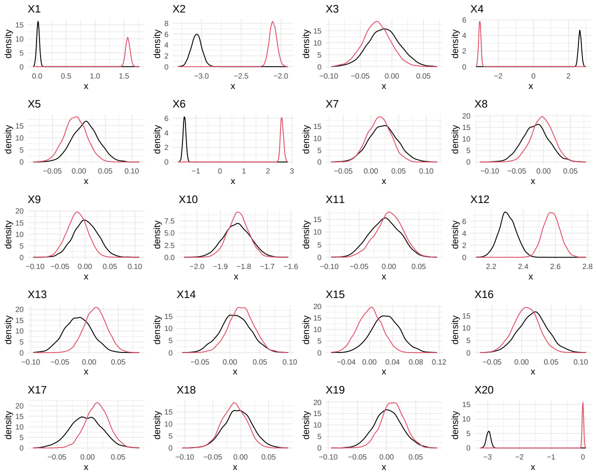

eight regression equations. Here, the non-null regression coefficients were drawn from the set $\{\pm 1.5, \pm 2\}$;

- three covariates (eleventh, twelfth, and thirteenth) strongly impact only three regression equations, with the non-null regression coefficients drawn from the set $\{\pm 3\}$;
- the remaining covariates have no impact in any regression equation.

Furthermore, we set $\alpha^+ = 50$, all elements of $\mathbf{p}$ equal to $1/D$, all elements of $\tilde{\mathbf{w}}$ equal to 0.7, $N = 1000$ and $n_i = 5000$. In Figure S7, we display the boxplots of the $B = 100$ posterior means of the inclusion probabilities for each covariate. These results clearly demonstrate that the proposed VS procedure reliably identifies the relevant covariates for inclusion in the analysis under typical threshold choices for the posterior means. In particular, threshold values between 0.1 and 0.2 provide a balanced control of both false negatives and false positives.

An important issue is the assessment of false negative risk, particularly in determining whether the model can detect a covariate that influences only a small subset of the response variables. As expected, the posterior mean inclusion probabilities for such covariates are lower than those for covariates that affect multiple responses. Nevertheless, in all scenarios considered—cases (I), (II), and (III)—these probabilities remain sufficiently high (exceeding 0.2) to allow detection under commonly used threshold values.

Moreover, comparison across the three cases reveals that these inclusion probabilities tend to decrease with increasing D, particularly when K is large.

These findings suggest that an appropriate threshold can be identified to balance false negatives and false positives and that this threshold should be tailored to the specific context.

2.2 Parameter estimation

To provide an idea of the inferential accuracy of the estimation methodology, we considered several simulation scenarios. The estimator performances were evaluated by deriving their posterior mean and relative mean squared error (rMSE). Empirical coverage of 95% credible sets (CSs) for each parameter was also computed.

Here we present four scenarios where the responses were generated from an EFDMReg model with one covariate (case IV), from a DMReg model with one covariate (case V), from a logistic-normal multinomial model with two covariates (case VI), and from an EFDMReg model with three covariates (case VII) respectively. Finally, we perform the second step of the two-step variable selection procedure applied in case III of Section 2.1.2, assessing the accuracy of the estimation procedure of the model with the 13 covariates selected from the initial set of 100 variables (case VIII).

2.2.1 Cases IV and V: data from an EFDMReg and from a DMReg with one covariate

In these two scenarios, we generated $B = 300$ microbiome responses with $N = 350$ samples and a number n_i of total taxa for each sample chosen according to a Poisson distribution with a parameter equal to 50.

The design matrix is common across replicas and is composed of a unit column and a single covariate $x_1 \sim \text{Unif}(-0.5, 0.5)$. In particular, we considered

Figure S7: Case III - Boxplot of the posterior mean of the probability of inclusion for each covariate across replications. Black dotted lines represent two reasonable thresholds (i.e., 0.1 and 0.2).

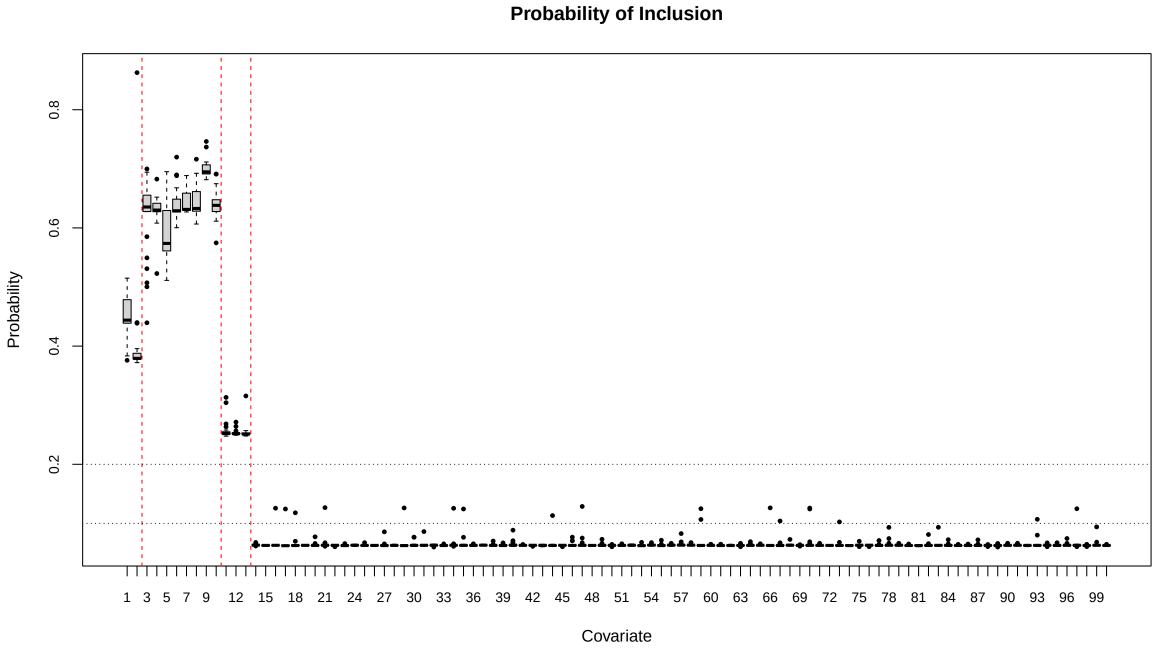

the following true values for the parameters:

$$\boldsymbol{\beta} = [\boldsymbol{\beta}_0^* \mid \boldsymbol{\beta}_1^*] = \begin{bmatrix} -0.5 & 1.8 \\ 1.5 & -2.5 \\ 2 & -1 \\ 3 & -2 \\ 0 & 0 \end{bmatrix},$$

and $\alpha^+ = 50$. Moreover, for case IV we set $\mathbf{p} = (0.25, 0.3, 0.2, 0.1, 0.15)^\intercal$, and $\tilde{\mathbf{w}} = (0.6, 0.3, 0.9, 0.4, 0.35)^\intercal$. The main results are reported in Tables S1 and S2.

Table S1: Case IV - Posterior Means, rMSEs of estimators, and coverages of the 95% CS.

	DMReg			FDMReg			EFDMReg		
Param.	Post. Mean	rMSE	Cov.	Post. Mean	rMSE	Cov.	Post. Mean	rMSE	Cov.
$\beta_{01} = -0.5$	-0.50	0.08	0.98	-0.46	0.20	0.89	-0.51	0.10	0.96
$\beta_{02} = 1.5$	1.02	0.48	0.00	1.44	0.09	0.85	1.50	0.07	0.95
$\beta_{03} = 2$	0.85	1.15	0.00	1.88	0.16	0.73	2.00	0.09	0.93
$\beta_{04} = 3$	2.14	0.86	0.00	2.96	0.07	0.92	3.01	0.06	0.96
$\beta_{11} = 1.8$	1.22	0.63	0.62	1.52	0.63	0.77	1.79	0.30	0.99
$\beta_{12} = -2.5$	-1.73	0.79	0.08	-2.20	0.39	0.70	-2.50	0.23	0.95
$\beta_{13} = -1$	0.34	1.37	0.00	-0.74	0.34	0.75	-0.99	0.20	0.95
$\beta_{14} = -2$	-1.54	0.49	0.49	-1.73	0.34	0.74	-2.00	0.21	0.94
$\alpha^+ = 50$	3.74	46.26	0.00	20.76	29.79	0.00	50.02	4.65	0.94
$p_1 = 0.25$	—	—	—	0.25	0.31	0.02	0.24	0.03	0.95
$p_2 = 0.3$	—	—	—	0.33	0.39	0.00	0.30	0.03	0.94
$p_3 = 0.2$	—	—	—	0.18	0.03	0.67	0.20	0.01	0.95
$p_4 = 0.1$	—	—	—	0.11	0.28	0.00	0.10	0.03	0.95
$p_5 = 0.15$	—	—	—	0.12	0.24	0.00	0.16	0.03	0.95
$\tilde{w}_1 = 0.6$	—	—	—	0.86	—	—	0.59	0.34	0.95
$\tilde{w}_2 = 0.3$	—	—	—	—	—	—	0.30	0.03	0.95
$\tilde{w}_3 = 0.9$	—	—	—	—	—	—	0.90	0.70	0.97
$\tilde{w}_4 = 0.4$	—	—	—	—	—	—	0.40	0.31	0.97
$\tilde{w}_5 = 0.35$	—	—	—	—	—	—	0.35	0.20	0.96
WAIC	—	10598.28	—	—	9059.02	—	—	8705.30	—

Table S1 reveals the high accuracy of the estimates under the EFDMReg model, with coverages very close to 0.95 for all parameters. Conversely, the other two models display worse performances, with the DMReg model showing remarkably negative behavior. Indeed, under this model, even in this simple case, the majority of regression coefficients are not well recovered, and in one case (namely β_{13}) the negative association is missed.

Conversely, when the true data-generating mechanism is the DMReg (Table S2) estimates are accurate under all models.

Table S2: Case V - Posterior Means, rMSEs of estimators, and coverages of the 95% CS.

	DMReg			FDMReg			EFDMReg		
Param.	Post. Mean	rMSE	Cov.	Post. Mean	rMSE	Cov.	Post. Mean	rMSE	Cov.
$\beta_{01} = -0.5$	-0.50	0.11	0.93	-0.48	0.11	0.96	-0.47	0.11	0.97
$\beta_{02} = 1.5$	1.51	0.07	0.94	1.50	0.07	0.95	1.48	0.07	0.97
$\beta_{03} = 2$	2.01	0.07	0.95	1.99	0.07	0.96	1.98	0.07	0.97
$\beta_{04} = 3$	3.01	0.07	0.94	2.99	0.07	0.97	2.98	0.07	0.97
$\beta_{11} = 1.8$	1.78	0.33	0.96	1.75	0.34	0.96	1.70	0.35	0.96
$\beta_{12} = -2.5$	-2.52	0.25	0.95	-2.50	0.25	0.94	-2.46	0.24	0.95
$\beta_{13} = -1$	-1.01	0.24	0.94	-1.00	0.24	0.94	-0.96	0.23	0.94
$\beta_{14} = -2$	-2.01	0.23	0.93	-2.00	0.23	0.95	-1.96	0.23	0.93
$\alpha^+ = 50$	50.40	4.41	0.95	56.45	11.12	0.82	48.81	7.53	0.80
p_1	—	—	—	0.23	—	—	0.16	—	—
p_2	—	—	—	0.31	—	—	0.42	—	—
p_3	—	—	—	0.19	—	—	0.22	—	—
p_4	—	—	—	0.05	—	—	0.10	—	—
p_5	—	—	—	0.22	—	—	0.10	—	—
$\tilde{\mathbf{w}}_1$	—	—	—	0.35	—	—	0.48	—	—
$\tilde{\mathbf{w}}_2$	—	—	—	—	—	—	0.48	—	—
$\tilde{\mathbf{w}}_3$	—	—	—	—	—	—	0.49	—	—
$\tilde{\mathbf{w}}_4$	—	—	—	—	—	—	0.48	—	—
$\tilde{\mathbf{w}}_5$	—	—	—	—	—	—	0.49	—	—
WAIC	—	5794.89	—	—	5802.85	—	—	5802.81	—

Table S3: Case VI - Normal correlation matrix.

$$\begin{bmatrix} 1.000 & 0.252 & -0.134 & 0.636 & 0.209 & -0.092 \\ 0.252 & 1.000 & 0.513 & 0.021 & 0.657 & -0.363 \\ -0.134 & 0.513 & 1.000 & 0.039 & 0.427 & -0.244 \\ 0.636 & 0.021 & 0.039 & 1.000 & -0.101 & -0.425 \\ 0.209 & 0.657 & 0.427 & -0.101 & 1.000 & -0.351 \\ -0.092 & -0.363 & -0.244 & -0.425 & -0.351 & 1.000 \end{bmatrix}$$

2.2.2 Case VI: data from a logistic-normal multinomial model with two covariates

Case VI pertains to a simulation study in which the data-generating mechanism is entirely different from our model. As in cases IV and V, we generated $B = 300$ microbiome responses with $N = 350$ samples and a number n_i of total taxa for each sample chosen according to a Poisson distribution with a parameter equal to 50. Differently, data are generated from a logistic-normal multinomial distribution, where the mean vector is regressed onto covariates, and the covariance (correlation) matrix is presented in Table S3. In the logistic-normal multinomial distribution, the probabilities $\mathbf{\Pi}$ are an additive log-ratio (alr) transformation of a normally distributed vector, that is $\mathbf{\Pi} = alr(\mathbf{Q})$ with $\mathbf{Q} \in \mathbb{R}^{D-1}$, and

$$\Pi_r = \begin{cases} \frac{\exp(Q_r)}{1+\sum_{l\neq D}\exp(Q_l)}, & r = 1, \ldots, D-1 \\ \frac{1}{1+\sum_{l\neq D}\exp(Q_l)}, & r = D \end{cases} .$$

Thus, we assume that

- $\mathbf{Y}_i|\mathbf{\Pi}_i = \boldsymbol{\pi}_i \sim$ multinomial$(n_i, \boldsymbol{\pi}_i)$;
- $\mathbf{\Pi}_i = alr(\mathbf{Q}_i)$
- $\mathbf{Q}_i$ is distributed as a multivariate normal: $\mathbf{Q}_i \sim N_6(\boldsymbol{\mu}_i, \Sigma)$.

We considered two covariates, namely $X_1\ Unif(-0.5, 0.5)$ and $X_2 \sim Bernoulli(0.3)$, so that

$$\mu_{i,r} = \beta_{0,r} + \beta_{1,r}x_{i,1} + \beta_{2,r}x_{i,2},$$

where intercepts and regression coefficients are reported in Table S4.

Furthermore, to evaluate the models in an even more challenging scenario, we exclude X_2 from the design matrix, thereby introducing a linear predictor misspecification.

The WAIC values for DMReg, FDMReg, and EFDMReg models computed over 300 replications are reported in Figure S8, and show that the additional parameters introduced in the FDMReg and EFDMReg models contribute to a better fit compared to the DMReg model, as indicated by the non-overlapping boxplots. Furthermore, the FDMReg and EFDMReg models exhibit similar WAIC values, with EFDMReg generally achieving lower scores in 98.3% of cases.

d	$\beta_{0,r}$	$\beta_{1,r}$	$\beta_{2,r}$
1	-0.5	1.8	1.5
2	1.5	-2.5	0
3	2	-1	-2
4	3	0.3	1.1
5	-0.3	1.3	0
6	1	-1	0.5

Table S4: Case VI - Regression coefficients.

Figure S8: Case VI - Boxplot of WAIC for each model across 300 replications.

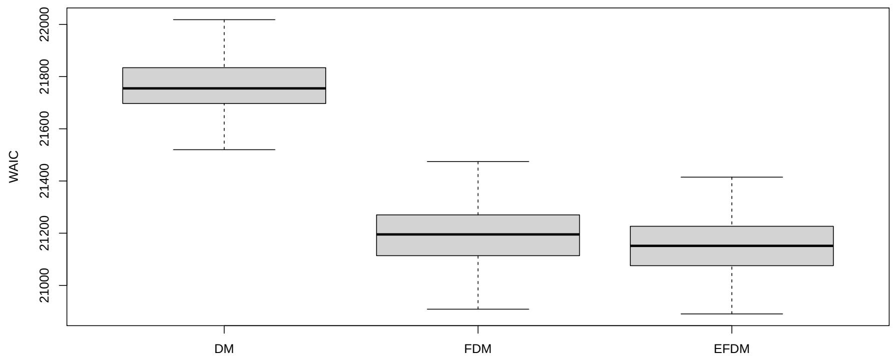

As for the estimates of the regression coefficients, note that a direct comparison with those from the logistic-normal multinomial model is not feasible, as they deserve a different meaning (different regression equations). Nonetheless, we report the estimates and their MSE in Table 2.2.2. The EFDMreg and FDMReg models generally demonstrate higher precision compared to the DMReg model.

2.2.3 Case VII: data from an EFDMReg with three covariates

This scenario involved $B = 150$ datasets, each composed $N = 400$ observations, generated from an EFDMReg model with $D = 5$ and considering three covariates (two continuous and one categorical) having an effect on the regression structure. More specifically, the covariates are distributed as follows:

- $X_1 \sim Unif(-0.5, 0.5)$;
- $X_2 \sim Bernoulli(0.3)$;
- $X_3 \sim Norm(0, 1)$.

	DMReg	FDMReg	EFDMReg
$\beta_{0,1}$	-0.010 (0.241)	-0.001 (0.251)	0.004 (0.251)
$\beta_{0,2}$	0.795 (0.498)	1.045 (0.211)	1.081 (0.211)
$\beta_{0,3}$	0.694 (1.707)	1.183 (0.670)	1.210 (0.670)
$\beta_{0,4}$	2.175 (0.683)	2.561 (0.196)	2.590 (0.196)
$\beta_{0,5}$	-0.131 (0.029)	-0.141 (0.027)	-0.139 (0.027)
$\beta_{0,6}$	0.565 (0.191)	1.085 (0.026)	0.996 (0.026)
$\beta_{1,1}$	0.733 (1.147)	0.875 (0.868)	0.903 (0.820)
$\beta_{1,2}$	-1.534 (0.947)	-1.559 (0.910)	-1.626 (0.791)
$\beta_{1,3}$	-0.492 (0.272)	-0.431 (0.350)	-0.471 (0.304)
$\beta_{1,4}$	0.295 (0.013)	0.062 (0.071)	0.026 (0.090)
$\beta_{1,5}$	0.533 (0.597)	0.622 (0.472)	0.640 (0.449)
$\beta_{1,6}$	-0.553 (0.215)	-0.563 (0.221)	-0.575 (0.205)

Table S5: Case VI - Posterior Means and MSE (in parenthesis) for each model.

To define vectors $\boldsymbol{\mu}_i$, $i = 1, \ldots, N$, we considered the regression parameters reported in Table S6. Please refer to Equation (22) of the main paper for the link between $\boldsymbol{\mu}_i$ and these parameters.

Table S6: Case VII - True parameters involved in defining the mean vectors $\boldsymbol{\mu}_i$.

r	$\beta_{0,r}$	$\beta_{1,r}$	$\beta_{2,r}$	$\beta_{3,r}$
1	-0.5	1.8	1.5	-1
2	1.5	-2.5	0	0.8
3	2	-1	-2	0
4	3	-2	1	-0.5
5	0	0	0	0

Additional parameters are set as follows: $\alpha^+ = 50$, $\mathbf{p} = (0.25, 0.3, 0.2, 0.1, 0.15)^\intercal$, and $\tilde{\mathbf{w}} = (0.6, 0.2, 0.9, 0.4, 0.3)^\intercal$.

Table S7 reports the posterior means, rMSEs and coverages of the models' parameters together with the values of WAICs. Moreover, Figure S9 shows the boxplots of the posterior distributions of the regression coefficients $\beta_{k,r}$ under the three models, and Figures S10 and S11 display the boxplots of the posterior distribution of parameters p_r and $\tilde{w}_r$ under the EFDMReg model.

The EFDMReg model achieves substantially lower WAIC values, confirming it as the best-fitting model for the simulated data. More notably, both the EFDMReg and FDMReg models yield more accurate estimates of the regression coefficients $\beta_{k,r}$ compared to the DMReg model, as evidenced by Figure S9. This improved accuracy in the estimation of the regression coefficients is achieved despite greater uncertainty in the estimates of the remaining parameters, as indicated by some intervals exhibiting limited coverage, particularly for the parameters $\tilde{w}_r$.

Table S7: Case VII - Posterior Means, rMSEs of estimators, and coverages of the 95% CS.

	DMReg			FDMReg			EFDMReg		
Param.	Post. Mean	rMSE	Cov.	Post. Mean	rMSE	Cov.	Post. Mean	rMSE	Cov.
$\beta_{0,1} = -0.5$	-0.534	0.111	0.987	-0.464	0.280	0.773	-0.531	0.141	0.933
$\beta_{0,2} = 1.5$	1.137	0.370	0.020	1.451	0.176	0.893	1.507	0.094	0.913
$\beta_{0,3} = 2$	0.844	1.161	0.000	1.679	0.384	0.300	1.944	0.129	0.893
$\beta_{0,4} = 3$	2.294	0.710	0.000	2.924	0.163	0.847	2.996	0.086	0.933
$\beta_{1,1} = 1.8$	1.261	0.111	0.987	1.725	0.280	0.773	1.732	0.141	0.933
$\beta_{1,2} = -2.5$	-1.960	0.370	0.020	-2.239	0.176	0.893	-2.508	0.094	0.913
$\beta_{1,3} = -1$	0.018	1.161	0.000	-0.748	0.384	0.300	-1.004	0.129	0.893
$\beta_{1,4} = -2$	-1.612	0.710	0.000	-1.748	0.163	0.847	-2.003	0.086	0.933
$\beta_{2,1} = 1.5$	1.120	0.616	0.713	1.399	0.548	0.813	1.481	0.339	0.940
$\beta_{2,2} = 0$	0.050	0.579	0.573	-0.077	0.361	0.813	0.008	0.259	0.953
$\beta_{2,3} = -2$	-1.490	1.087	0.140	-1.685	0.350	0.827	-1.994	0.238	0.927
$\beta_{2,4} = 1$	0.818	0.434	0.673	0.912	0.344	0.833	1.004	0.244	0.933
$\beta_{3,1} = -1$	-0.762	0.616	0.713	-0.913	0.548	0.813	-0.993	0.339	0.940
$\beta_{3,2} = 0.8$	0.579	0.579	0.573	0.771	0.361	0.813	0.804	0.259	0.953
$\beta_{3,3} = 0$	0.123	1.087	0.140	0.023	0.350	0.827	0.007	0.238	0.927
$\beta_{3,4} = -0.5$	-0.428	0.434	0.673	-0.466	0.344	0.833	-0.495	0.244	0.933
$\alpha^{+} = 50$	4.502	45.499	0.000	21.497	29.453	0.000	47.888	9.659	0.627
$p_1 = 0.25$	—	—	—	0.261	0.282	0.153	0.227	0.034	0.853
$p_2 = 0.3$	—	—	—	0.332	0.376	0.000	0.277	0.163	0.587
$p_3 = 0.2$	—	—	—	0.154	0.054	0.240	0.190	0.016	0.887
$p_4 = 0.1$	—	—	—	0.104	0.272	0.000	0.095	0.096	0.780
$p_5 = 0.15$	—	—	—	0.150	0.252	0.000	0.210	0.164	0.520
$\tilde{w}_1 = 0.6$	—	—	—	0.818	—	—	0.557	0.309	0.833
$\tilde{w}_2 = 0.2$	—	—	—	—	—	—	0.311	0.089	0.160
$\tilde{w}_3 = 0.9$	—	—	—	—	—	—	0.889	0.689	0.593
$\tilde{w}_4 = 0.4$	—	—	—	—	—	—	0.441	0.345	0.887
$\tilde{w}_5 = 0.3$	—	—	—	—	—	—	0.411	0.266	0.360
WAIC	—	8380.985	—	—	7432.220	—	—	7066.361	—

Figure S9: Case VII - Boxplots of the posterior distribution of the regression coefficients $\beta_{k,r}$ under the three models.

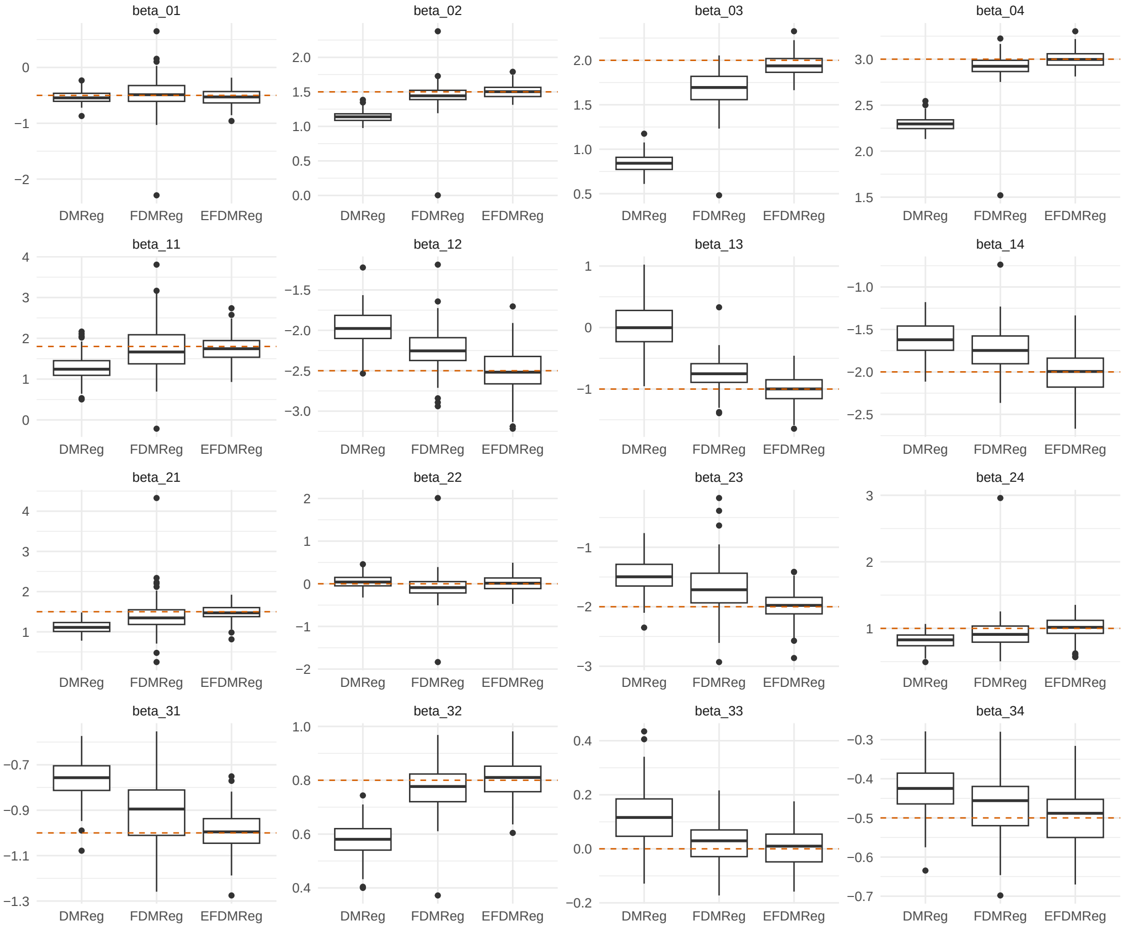

2.2.4 Case VIII: estimation of the model with the 13 covariates selected in case III

To further assess the performance of the EFDMReg model in scenarios characterized by large values of D and K, we extended the analysis presented in Section 2.1.2 by implementing the second step of the proposed VS procedure, namely, fitting the EFDMReg model on the subset of selected covariates.

This second fitting step enables more accurate parameter estimation. Specifically, by evaluating the bias (i.e., the difference between the posterior mean and the true value of each parameter), we observe overall reliable estimates. As shown in Figure S12, the biases associated with each covariate (represented by

Figure S10: Case VII - Boxplots of the posterior distribution of the parameters p_r under the EFDMReg model.

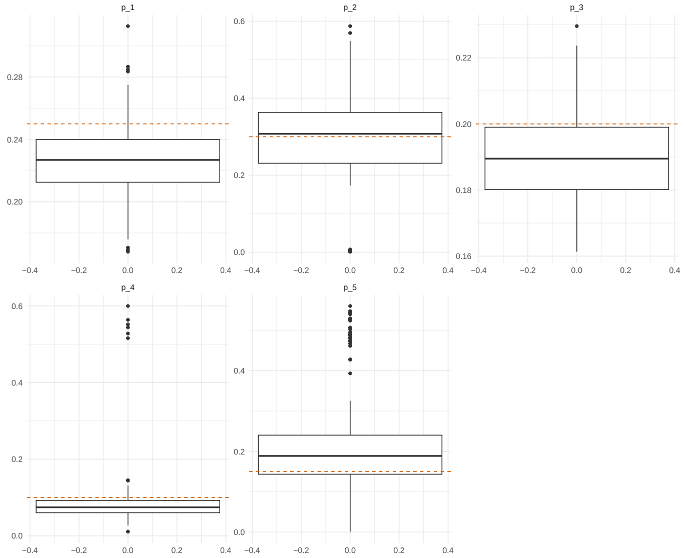

Figure S11: Case VII - Boxplots of the posterior distribution of the parameters $\tilde{w}_r$ under the EFDMReg model.

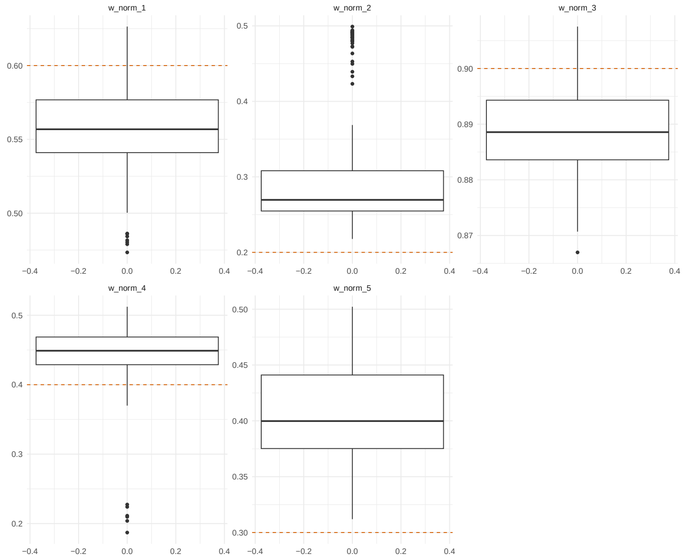

the boxplots across the $D-1$ regression equations) are generally centered around

zero and exhibit limited variability. This indicates that the proposed procedure leads to low-bias parameter estimates, even in high-dimensional settings.

Figure S12: Case VIII: boxplots of the approximated biases for the regression coefficients associated with each covariate, computed across the $D-1$ regression equations.

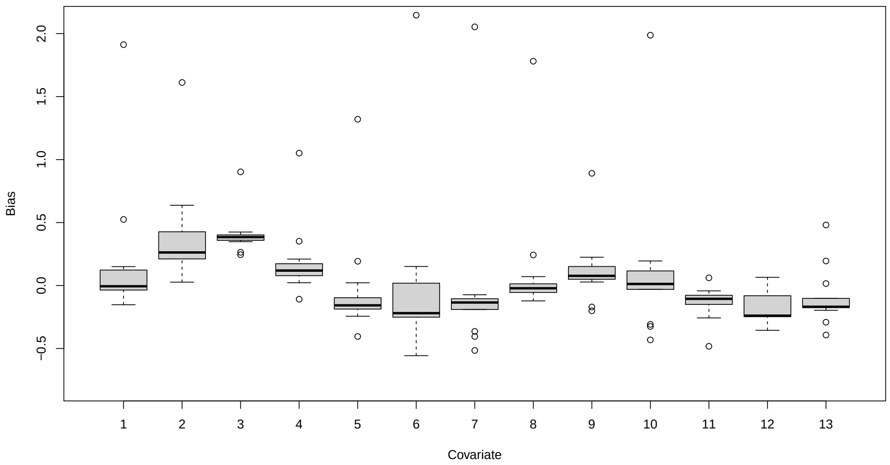

As for the other parameters, the precision parameter $\alpha^+ = 50$ is accurately estimated, with an approximate bias of 0.933. Regarding the parameters $\mathbf{p}$ and $\tilde{\mathbf{w}}$, their estimated biases lie within the ranges $(-0.0039, 0.0122)$ for the elements of $\mathbf{p}$, and $(-0.1957, -0.0065)$ for those of $\tilde{\mathbf{w}}$. These results indicate a good estimation accuracy for both parameters, with particularly small biases for $\mathbf{p}$ and slightly larger, yet still acceptable, biases for $\tilde{\mathbf{w}}$.

Considering all the performed simulation studies, under the EFDMReg model, good inferential accuracy has been found in estimating regression coefficients even for large values of K and D. However, note that in the more complex simulation settings, a larger uncertainty has been detected in estimating cluster parameters, i.e., p_r and $\tilde{w}_r$ $(r = 1, \ldots, D)$.

2.3 Correlation estimation

In this section, we present the results of two additional simulation studies (cases IX and X) designed to assess the ability of the EFDM model to recover various correlation structures, with a particular focus on positive associations that the usual DM model fails to capture.

In both scenarios, models were estimated without covariates with $n_i = n = 5000$ (i.e., the same number of bacterial reads for each observation).

Table S8: Case IX - EFDM correlation matrix.

$$\begin{bmatrix} 1 & 0.503 & 0.189 & -0.194 & -0.415 \\ 0.503 & 1 & 0.105 & -0.237 & -0.428 \\ 0.189 & 0.105 & 1 & -0.341 & -0.461 \\ -0.194 & -0.237 & -0.341 & 1 & -0.478 \\ -0.415 & -0.428 & -0.461 & -0.478 & 1 \end{bmatrix}$$

Table S9: Case IX - EFDM estimated correlation matrix averaged across 300 replications.

$$\begin{bmatrix} 1.000 & 0.527 & 0.196 & -0.202 & -0.424 \\ 0.527 & 1.000 & 0.123 & -0.242 & -0.440 \\ 0.196 & 0.123 & 1.000 & -0.333 & -0.460 \\ -0.202 & -0.242 & -0.333 & 1.000 & -0.477 \\ -0.424 & -0.440 & -0.460 & -0.477 & 1.000 \end{bmatrix}$$

2.3.1 Case IX: data from EFDM model with large positive correlations

The first scenario (case IX) involves 300 samples from an EFDM distribution characterized by $D = 5$. The parameters:

- $\boldsymbol{\mu} = (0.09, .17, .23, .25, .26)$;
- $\tilde{\mathbf{w}} = (.1, .25, .5, .75, .9)$;
- $\alpha^+ = 50$;
- $\mathbf{p} = (.2, .2, .2, .2, .2)$.

have been selected to introduce three positive correlations among the ten possible ones, thus leading to the correlation matrix reported in Table S8.

We fitted the DM, FDM, and EFDM models to the 300 samples. The results indicate that both the DM and FDM models not only fail to capture positive associations but also exhibit poor model fit, as evidenced by higher average WAIC values (21,514.03 and 21,137.38, respectively) compared to the substantially lower WAIC of 18,286.90 for the EFDM model. Focusing on EFDM, Figures S13—S16 confirm the ability of the estimation procedure to recover the true parameters' value.

This results in an accurate estimation of the correlation structure, as evidenced by Table S9, which reports the average of the 300 estimated correlation matrices. All estimated correlations closely approximate the corresponding theoretical values reported in S8. Notably, the EFDM correctly identifies the three large positive correlations.

Following a referee's suggestion, we calculated the average of a nonparametric estimate of associations (using Spearman's rank correlation index) across

Figure S13: Case IX - Boxplots of estimates for each element of $\boldsymbol{\mu}$ across 300 replications.

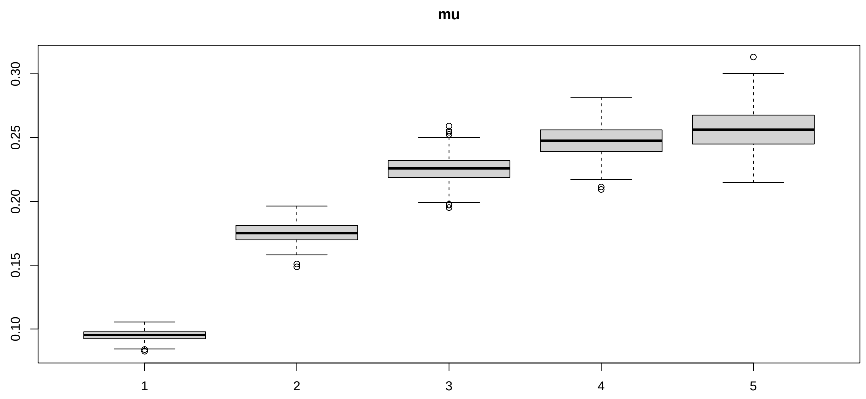

Figure S14: Case IX - Boxplots of estimates for each element of $\mathbf{p}$ across 300 replications.

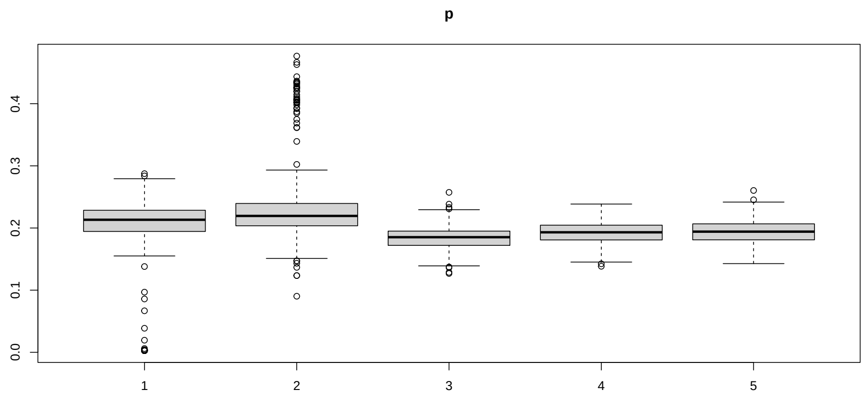

the 300 samples, resulting in matrix S10. It should be noted that, using this method, the number and magnitude of positive associations are significantly greater than those in matrix S8 corresponding to the data-generating model.

Figure S15: Case IX - Boxplots of estimates for each element of $\tilde{\mathbf{w}}$ across 300 replications.

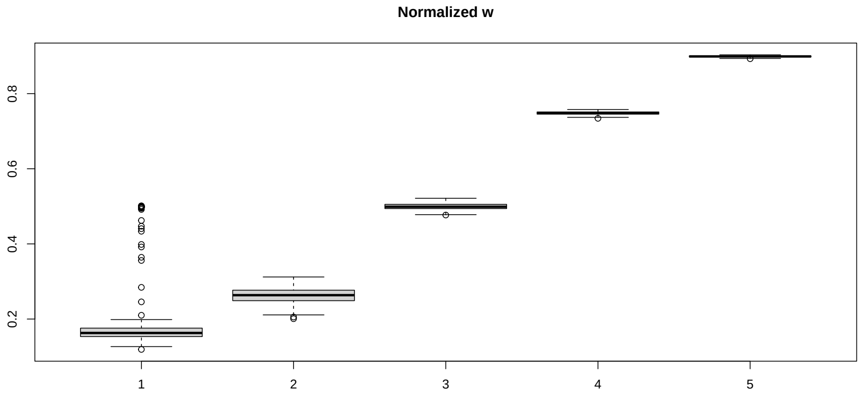

Figure S16: Case IX - Boxplot of estimates for the parameter α^+ across 300 replications.

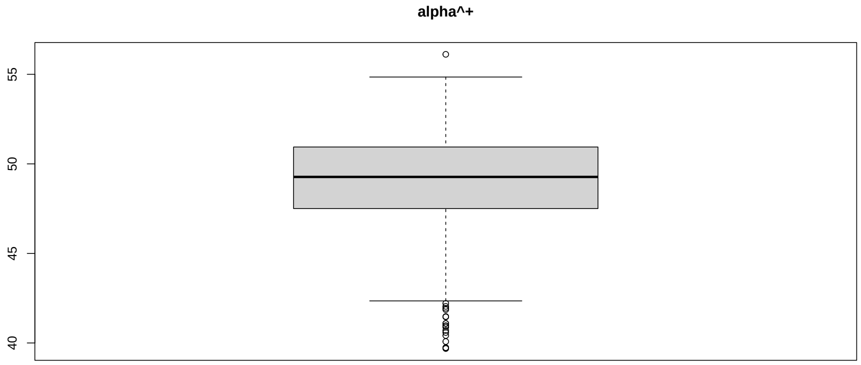

2.3.2 Case X: data from a mixture of logistic-normal multinomial

To further evaluate the ability of the EFDM model to recover correlations, we consider a scenario (case X) involving a complex data-generating mechanism that substantially differs from our model. Specifically, the data are generated using a mixture of logistic-normal multinomial distributions with $D = 7$ as follows:

- $\mathbf{Y}|\mathbf{\Pi} = \boldsymbol{\pi} \sim$ multinomial$(n, \boldsymbol{\pi})$;

Table S10: Case IX - Spearman rank correlation matrix averaged across 300 replications.

$$\begin{bmatrix} 1.000 & 0.798 & 0.618 & 0.280 & -0.116 \\ 0.798 & 1.000 & 0.576 & 0.257 & -0.138 \\ 0.618 & 0.576 & 1.000 & 0.050 & -0.331 \\ 0.280 & 0.257 & 0.050 & 1.000 & -0.677 \\ -0.116 & -0.138 & -0.331 & -0.677 & 1.000 \end{bmatrix}$$

- $\mathbf{\Pi}$ is obtained by applying the *additive log-ratio transformation (alr)* to a vector $\mathbf{Q} \in \mathbb{R}^{D-1}$: $\mathbf{\Pi} = alr(\mathbf{Q})$, namely,

$$\Pi_r = \begin{cases} \frac{\exp(Q_r)}{1+\sum_{l \neq D} \exp(Q_l)}, r = 1, \ldots, D-1 \\ \frac{1}{1+\sum_{l \neq D} \exp(Q_l)}, r = D \end{cases} ;$$

- $\mathbf{Q}$ is distributed according to a mixture of D multivariate Normal components of dimension $D-1$, each having its own barycentre and common covariance matrix. Thus, the p.d.f. of $\mathbf{Q}$ can be expressed as $f_{\mathbf{Q}}(\mathbf{q}; \cdot) = \sum_{r=1}^{D} p'_r f_N(\mathbf{q}; \boldsymbol{\mu}_r; \Sigma)$.

The component-specific barycentres and the shared covariance (correlation) matrix are the following:

- $\boldsymbol{\mu}_1 = (0, 0, 0, 0, 0, 0)^\intercal$;
- $\boldsymbol{\mu}_2 = (0.57, 0.45, 0.12, 1.03, 0.14, 0.56)^\intercal$;
- $\boldsymbol{\mu}_3 = (0.41, 3.860.71, 4.65, 0.83, 3.20)^\intercal$;
- $\boldsymbol{\mu}_4 = (1.01, 0.02, 3.47, 3.64, 3.17, 2.54)^\intercal$;
- $\boldsymbol{\mu}_5 = (-3.20, -3.47, -2.90, -4.28 - 3.15, -2.20)^\intercal$;
- $\boldsymbol{\mu}_6 = (4.77, 3.76, 4.67, 4.83, 4.50, 3.94)^\intercal$;
- $\boldsymbol{\mu}_7 = (-3.46, 4.58, 1.17, 0.94, -8.24, 2.99)^\intercal$,

and

Please note that $\boldsymbol{\mu}_1$ has been set to the null vector, while the remaining vectors have been sampled from different univariate Normal distributions to account for varying barycenters.

It is worth emphasizing that the correlation matrix in S11 pertains to log-ratios. As highlighted in the main paper, the first- and second-order moments of log-ratios do not have a correspondence with the moments of the original simplex-valued variables. The matrix S12 reports a Monte Carlo approximation

Table S11: Case X - True (log-ratio) correlation matrix.

1.000	0.252	−0.134	0.636	0.209	−0.092
0.252	1.000	0.513	0.021	0.657	−0.363
−0.134	0.513	1.000	0.039	0.427	−0.244
0.636	0.021	0.039	1.000	−0.101	−0.425
0.209	0.657	0.427	−0.101	1.000	−0.351
−0.092	−0.363	−0.244	−0.425	−0.351	1.000

Table S12: Case X - Monte Carlo approximation of the true correlation matrix.

1.000	−0.303	−0.066	0.049	0.192	−0.140	−0.087
−0.303	1.000	−0.310	−0.265	−0.329	−0.080	−0.306
−0.066	−0.310	1.000	−0.079	0.388	−0.192	−0.177
0.049	−0.265	−0.079	1.000	−0.118	−0.222	−0.382
0.192	−0.329	0.388	−0.118	1.000	−0.236	−0.190
−0.140	−0.080	−0.192	−0.222	−0.236	1.000	−0.134
−0.087	−0.306	−0.177	−0.382	−0.190	−0.134	1.000

of the correlation matrix under this challenging data-generating mechanism. Notably, three of the correlations are positive.

We fitted the DM, FDM, and EFDM models to the 300 samples. Among these models, the DM consistently exhibited the poorest performance, with an average WAIC of 29656.7. The FDM achieved slightly better results (WAIC = 29495.47). In contrast, the EFDM obtained the lowest WAIC (29140.16), indicating that its greater flexibility is beneficial for capturing a wider range of data-generating mechanisms.

To recover the correlation structures, we estimated the EFDM correlation matrix over the 300 replications. The matrix S13 presents the average of the 300 estimated correlation matrices and reveals that two correlations are positive, specifically those corresponding to the strongest positive associations involving pairs (Y_1, Y_5) and (Y_3, Y_5). Interestingly, these two correlations are consistently estimated as positive across all 300 datasets, although their estimated magnitudes are generally significantly lower than the true values.

As suggested by a referee, we finally computed the Spearman correlation coefficients across the 300 simulated datasets and averaged them (Table S14). In this case, as well, the number and magnitude of positive associations are significantly greater than those in the matrix corresponding to the data-generating model.

In summary, these results suggest that, even when applied to data generated from markedly different models, the EFDM is capable of partially recovering positive associations. However, it is evident that the model's correlation structure is not flexible enough to accommodate all potential cases of interest. This limitation is also theoretically justified, as the number of parameters in

Table S13: Case X - EFDM estimated correlation matrix averaged across 300 replications.

1.000	−0.159	−0.011	−0.082	0.043	−0.036	−0.144
−0.159	1.000	−0.209	−0.298	−0.226	−0.267	−0.286
−0.011	−0.209	1.000	−0.107	0.061	−0.048	−0.189
−0.082	−0.298	−0.107	1.000	−0.147	−0.169	−0.267
0.043	−0.226	0.061	−0.147	1.000	−0.033	−0.233
−0.036	−0.267	−0.048	−0.169	−0.033	1.000	−0.243
−0.144	−0.286	−0.189	−0.267	−0.233	−0.243	1.000

Table S14: Case X - Spearman rank correlation matrix averaged across 300 replications.

1.000	−0.341	0.311	0.273	0.622	−0.110	0.183
−0.341	1.000	−0.333	−0.181	−0.407	−0.028	−0.184
0.311	−0.333	1.000	0.054	0.667	−0.155	0.021
0.273	−0.181	0.054	1.000	0.176	−0.161	−0.376
0.622	−0.407	0.667	0.176	1.000	−0.222	−0.004
−0.110	−0.028	−0.155	−0.161	−0.222	1.000	0.159
0.183	−0.184	0.021	−0.376	−0.004	0.159	1.000

the EFDM model increases linearly with D, rather than quadratically, as would be necessary to model an arbitrary covariance matrix. While this linear growth reduces computational complexity, particularly for moderate or large values of D, it inevitably imposes some flexibility constraints.

2.4 Scalability

To show the performance of the proposed EFDMReg model in the case of increasing values of D, we set up three simulation studies considering D equal to 10, 30, and 50, respectively. In particular, we generated data from the EFDMReg model with one continuous covariate for each scenario. Since the number of parameters increases with D (i.e., it is equal to $2D+(D-1)(K+1)$, where K is the number of covariates), the estimation procedure requires more information (i.e., data points) to provide adequate estimates. Thus, we considered a sample size equal to $N = 350$ for $D \in \{10, 30\}$ and $N = 500$ for $D = 50$.

Due to the large number of parameters, we generated their values according to the following scheme:

- the $2(D-1)$ β_{dk} parameters have been drawn from a Uniform distribution on the interval $(-2, 2)$;
- $\alpha^+ = 50$;

- the vector $\mathbf{p}$ has been generated from a Dirichlet distribution with barycentre equal to $(1/D, \ldots, 1/D)^\intercal$;
- each $\tilde{w}_r$ has been drawn from a Uniform distribution on the interval $(0.1, 0.95)$.

We found out that the estimation procedure is also reliable with high values of D, once an adequate sample size is considered. For example, inspecting Figure S17, it emerges that the coverage levels of the 95% credible sets for the $D-1$ regression coefficients are close to the nominal ones, and these levels seem to be stable even for large values of D. The same holds for the coverage levels related to the other parameters.

Figure S17: Empirical coverages of the 95% credible sets for the regression coefficients.

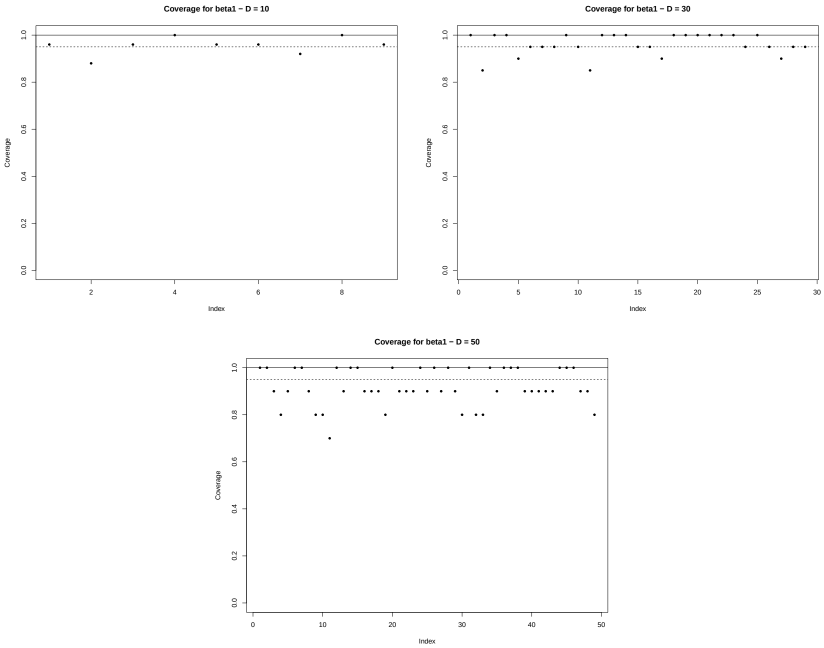

In Figure S18, we can inspect the computational time (expressed in seconds) required by one iteration of the EFDMReg's estimation procedure (the boxplot refers to $B = 25$ replications). As expected, the computational time increases as D increases due to the larger number of parameters and sample size, though retaining feasibility.

The scalability of the model with respect to K for moderately large values of D is demonstrated by scenario (III) of Section 2.1.2 where $D = 15$, $K = 100$, and the variable selection model is considered. In particular, the computational burden is still manageable (58 seconds for one replication).

For values of D larger than 50, the scalability issue of the EFDMReg model needs further deepening. However, the computational time of the estimation process can benefit from informative prior distributions. For example, in the presence of microbes with all low abundances, it is reasonable to a priori exclude the corresponding mixture components, thus reducing the parameter dimension.

Figure S18: Computational time required to fit the EFDMReg model.

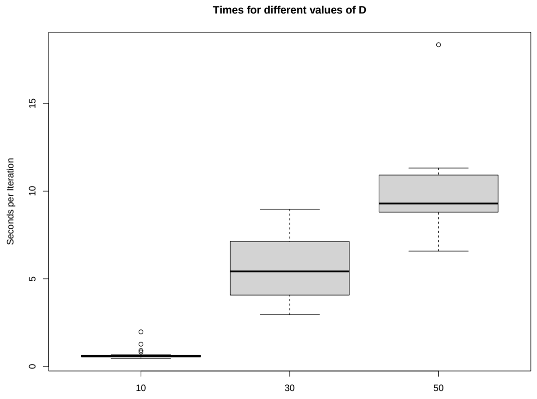

3 Simulation study 2: Comparison of EFDMReg and ZIDM models (Section 4.3 of the paper)

Unlike the DM model, the mixture structure of the EFDMReg model allows us to address excessive zeros by devoting different components of the mixture to zero and, respectively, positive counts handling. In particular, in the EFDMReg model, two parameters (namely, α_r and τ_r) are devoted to each count y_r ($r = 1, \dots, D$). In the presence of excessive zeros, this flexibility can be exploited to account for both zeros through a low value of α_r, and positive counts through a suitably high value of τ_r acting on the r-th mixture component. An example of this behavior is shown in Section 3.1 (see Figure S20 and related comments).

An alternative approach is to build zero-inflated models with an explicit structural zero component. Although interesting this approach is not exempt from drawbacks (see [5] for an in-depth discussion). In practice (that is relying purely on observations without external information), the main source of difficulty lies in untangling sampling zeros and structural zeros. Deciding whether an observed zero is generated by low abundance or absence seems problematic from an inferential point of view unless one has a very large number of observations. The uncertainty in recovering the true nature of zero counts leads in some cases to incorrect inferential conclusions. In particular, the presence of a structural component in the model may often induce an excessive number of structural zeros, as will be shown through simulations (Sections 3.1 and 3.2) and real data analysis (Section 5.2.3). This miss-classification, in turn, has consequences on the estimation of the abundances, which are typically biased upward when many zero counts are present.

Here we compare the EFDMReg model with the zero-inflated Dirichlet-multinomial (ZIDM) model by [2]. To such end, we perform extensive simulations considering various types of zero-generating mechanisms. In the following, we report the results relative to two mechanisms (Sections 3.1 and 3.2). In the first one, we generated data from a model different from both the EFDMReg and the ZIDM models. In particular, data were drawn from a DMReg model, and a fraction of randomly selected counts was replaced with zeros. In the second simulation, we generated data from a ZIDM model.

It is not possible to compare the ZIDM and the EFDMReg models in terms of regression coefficients because the structure of the relations between covariates and response variables is quite different. Specifically, the EFDMReg model explains the (scaled) mean of each count (as the inverse logit of the linear predictor). Therefore, its regression coefficients have a clear-cut interpretation in terms of log-odds ratios with respect to a baseline category. On the contrary, the ZIDM models the γ_j parameters defining the Dirichlet distribution (as inverse logarithms of the linear predictor). These parameters jointly involve both the mean and the variance of the counts, which makes their interpretation less evident. In particular, note that the signs of these coefficients do not determine, in general, the type of associations (positive or negative) between the

covariates and the mean of counts. For example, it is clear that when all regression coefficients relative to a given covariate are positive, that covariate cannot have an increasing influence on all count data, given that their sum is fixed. In fact, it is simple to show that the smallest of these regression coefficients always determines a monotonically decreasing relation (even when it is positive) and the highest implies an increasing relation (even when negative). Typically, non-monotonic relations are present in the other cases. Therefore, care must be paid in the interpretation of ZIDM coefficients.

Moreover, the ZIDM and the EFDMReg models can not be compared based on some goodness-of-fit criterion (e.g. WAIC) since the ZIDM does not admit a closed-form expression for its log-likelihood function.

Therefore, we decided to base the models' comparison on their predictive ability in terms of zero-count handling and global accuracy. As for zeros, we considered standard measures such as sensitivity, specificity, positive predictive value (PPV), and negative predictive value (NPV). Concerning global accuracy we quantified the prediction error through the average Kullback-Leibler (KL) divergence of the predicted from the observed counts.

Specifically, let $\boldsymbol{\theta}^{(b)}$ denote the b-th element generated from the simulated joint posterior distribution of $\boldsymbol{\theta}$, namely the vector of all the parameters involved in a given regression model $f_{\mathbf{Y}}(\mathbf{y}|\boldsymbol{\theta}, \mathbf{x})$. Then, we can use $\boldsymbol{\theta}^{(b)}$ to generate a completely new response matrix $\mathbf{Y}^{*(b)}$ by drawing $\mathbf{y}_i^{*(b)}$ from $f_{\mathbf{Y}}(\mathbf{y}|\boldsymbol{\theta}^{(b)}, \mathbf{x}_i)$. This approach allows us to have a complete (simulated) distribution, i.e., the posterior predictive distribution. Based on this distribution the following measures can be computed:

- Sensitivity$^{(b)}$ $= \sum_{i=1}^{N}\sum_{d=1}^{D} \mathbb{I}\left(Y_{i,d}^{*(b)} = 0, Y_{i,d} = 0\right) / \# \{Y_{i,d} = 0\}$, that is, the proportion of true zero counts correctly predicted as zero by a model.

- Specificity$^{(b)}$ $= \sum_{i=1}^{N}\sum_{d=1}^{D} \mathbb{I}\left(Y_{i,d}^{*(b)} \neq 0, Y_{i,d} \neq 0\right) / \# \{Y_{i,d} \neq 0\}$, that is, the proportion of true non-zero counts correctly predicted as non-zero by a model.

- Positive Predictive Value: $\text{PPV}^{(b)} = \sum_{i=1}^{N}\sum_{d=1}^{D} \mathbb{I}\left(Y_{i,d}^{*(b)} = 0, Y_{i,d} = 0\right) / \# \left\{Y_{i,d}^{*(b)} = 0\right\}$, that is, the proportion of zero predictions that correspond to zero observed counts.

- Negative Predictive Value: $\text{NPV}^{(b)} = \sum_{i=1}^{N}\sum_{d=1}^{D} \mathbb{I}\left(Y_{i,d}^{*(b)} \neq 0, Y_{i,d} \neq 0\right) / \# \left\{Y_{i,d}^{*(b)} \neq 0\right\}$, that is, the proportion of non-zero predictions that correspond to non zero observed counts.

Moreover, we can compute the average KL divergence of the predicted responses from the observed responses as:

$$\bar{d}_{KL}^{(b)} = \frac{1}{N}\sum_{i=1}^{N} d_{KL}\left(\frac{y_{i,d}}{n_i}, \frac{y_{i,d}^{*(b)}}{n_i}\right) = \frac{1}{N}\sum_{i=1}^{N}\sum_{d=1}^{D}\frac{y_{i,d}}{n_i}\log\left(\frac{y_{i,d}/n_i}{y_{i,d}^{*(b)}/n_i}\right).$$

When $y_{i,d} = 0$ the natural choice is to set to zero the corresponding term in the KL divergence. Specifically:

- when $y_{i,d}$ and $y_{i,d}^{*(b)}$ are both equal to zero, i.e., observed and predicted counts coincide, no prediction error is present.
- when $y_{i,d} = 0$ and $y_{i,d}^{*(b)} \neq 0$, this choice is justified by continuity as limit $\lim_{x \to 0} x \log x = 0$.

The critical case is when $y_{i,d} \neq 0$ and $y_{i,d}^{*(b)} = 0$. In this case, the traditional solution is to add a small positive value ε to $y_{i,d}^{*(b)}$. We chose $\varepsilon = 1$ as this is the value that operates the maximum reduction of the error among the values that systematically (that is, for any positive $y_{i,d}$) reduce the error. Thus, this choice favors models with a high rate of incorrectly predicted zeros (low specificity) as will be shown to be the case of the ZIDM model. Notice that more widespread choices for ε (values closer to zero) would produce poorer comparative performances for zero-inflated models with low specificity (for completeness we report results for $\varepsilon = 0.5$ as well).

3.1 Data from DM with an excess of zeros in two elements of the response

In this scenario, we drew data from a standard DMReg model with $\alpha^+ = 50$ and

$$\boldsymbol{\beta} = [\boldsymbol{\beta}_0^* \mid \boldsymbol{\beta}_1^*] = \begin{bmatrix} -0.5 & 1.8 \\ 1.5 & -2.5 \\ 2 & -1 \\ 3 & -2 \\ 0 & 0 \end{bmatrix}.$$

The covariate was generated from a uniform distribution with a range $(-0.5,\ 0.5)$ and the values of n (bacterial reads) from a Poisson(100). Furthermore, we randomly selected 15% and 25% of data points and set the corresponding entries for the third and, respectively, the fifth taxon to zero. To preserve the total number of taxa reads, we reallocated the removed taxa counts to the first element of $\mathbf{Y}$. We used this mechanism to generate 300 datasets of size equal to 350. We also considered different percentages of zeros, a different number of taxa, and a different allocation scheme with similar results.

With respect to zero-handling, Table S15 and Figure S19 show that the ZIDM regression model captures a much higher number of true zeros (sensitivity) than the EFDMReg model, however, a lower percentage of predicted zeros is correct (PPV). As for positive counts, the EFDMReg model succeeds in recognizing a much higher fraction of true positive counts (specificity) still having good behavior in terms of the percentage of correctly predicted positive counts (NPV). This comparison highlights that no model completely dominates the other in zero handling, whereas the EFDMReg performs consistently better when positive counts are involved. As a consequence, the EFDMReg model displays an overall better performance. For example, the accuracy (i.e., the ratio between the number of correct predictions and the total number of predictions) of the EFDMReg model is substantially larger (see Table S15).

Table S15: Data from DM with an excess of zeros. Summaries of Sensitivity, Specificity, PPV, NPV, and accuracy measures across the 300 replications.

Metric	Model	Min	1st Qu.	Mean	SD	Median	3rd Qu.	Max
Sensitivity	EFDM	0.306	0.330	0.339	0.014	0.339	0.347	0.379
	ZIDM	0.804	0.809	0.811	0.003	0.811	0.812	0.822
Specificity	EFDM	0.887	0.892	0.895	0.004	0.894	0.897	0.907
	ZIDM	0.238	0.241	0.242	0.001	0.242	0.243	0.247
PPV	EFDM	0.355	0.386	0.397	0.017	0.395	0.407	0.450
	ZIDM	0.165	0.175	0.179	0.006	0.179	0.183	0.203
NPV	EFDM	0.860	0.867	0.869	0.003	0.869	0.871	0.878
	ZIDM	0.854	0.860	0.863	0.003	0.863	0.865	0.871
Accuracy	EFDM	0.791	0.798	0.801	0.004	0.800	0.803	0.812
	ZIDM	0.329	0.336	0.338	0.004	0.338	0.341	0.356

Figure S19: Data from DM with an excess of zeros. Boxplots of the posterior predicted means of sensitivity, specificity, PPV, NPV, and accuracy by model computed on 300 simulated datasets.

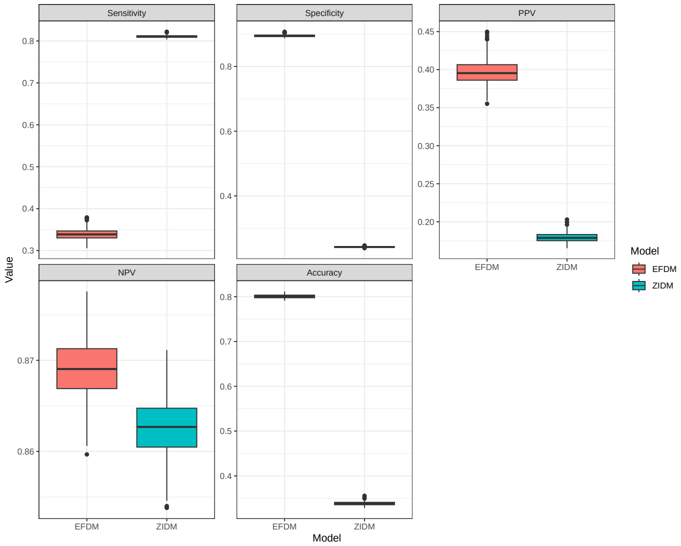

To better understand how the EFDMReg model can handle an excessive number of zeros we report the scatterplot of one randomly selected replication together with the regression curves for the two mixture components with positive estimated weights (namely the first and the third) for Y_1 and Y_3 (see Figure S20). As for Y_1 (left panel) where no excess of zeros is present, the two component-specific regression curves $\boldsymbol{\lambda}_1$ and $\boldsymbol{\lambda}_3$ capture low and, respectively high values. Conversely, for Y_3 (right panel) the excessive number of zeros is modeled by the first component-specific regression $\boldsymbol{\lambda}_1$.

Concerning the global accuracy of the predicted counts, the KL divergence shows a remarkably lower error measure for the EFDMReg model (see Figure S21).

3.2 Data from ZIDM

In this scenario, we simulate the response vector from a ZIDM regression model [2]. In particular, the true data-generating mechanism considers one covariate (X_1) that affects the mean regression. To generate zero inflation within the ZIDM model, we used an independent auxiliary variable (X_2) affecting the structural zeros component of the model. We used this mechanism to generate 300 datasets of size equal to 350 and generated X_1 from a N(0,4), X_2 from a N(0,1) and n (bacterial reads) from a Poisson(100). These data sets have, on average, 26.2% zeros in the first element of the response, 24.8% in the second, 14% in the third, 11.8% in the fourth, and 16.4% in the fifth.

Figure S20: Data from DM with an excess of zeros. Scatterplot of Y_1 (left) and Y_3 (right) vs. X for one randomly selected replication. Red dashed and pink dot-dashed lines represent the regression curves for the two EFDMReg's non-empty mixture components (namely $\boldsymbol{\lambda}_1$ and $\boldsymbol{\lambda}_3$, respectively). The black solid curve represents the overall EFDMReg's fitted $\boldsymbol{\mu}_i$.

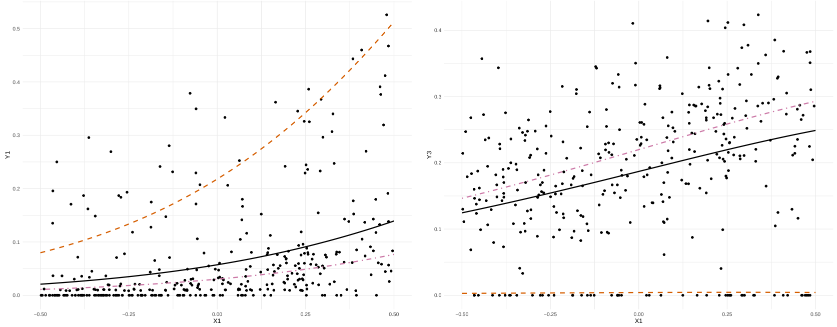

Figure S21: Data from DM with an excess of zeros. Posterior mean of the average KL divergence for the ZIDM and the EFDMReg models for $\varepsilon = 0.5$ and $\varepsilon = 1$.

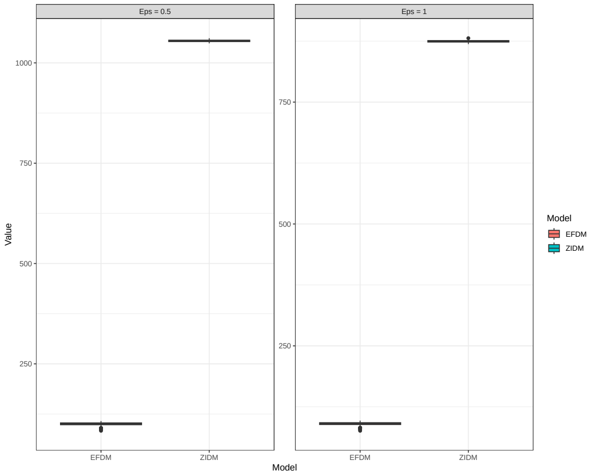

Then, the EFDMReg and ZIDM models have been fitted on the 300 simulated datasets by considering just X_1 in the mean regression structure.

Table S16 and Figure S22 deserve remarks similar to the case of Section 3.1 with only a slightly worse performance of the EFDMReg model. In particular, the overall performance measures, i.e., accuracy and KL divergence, still greatly favor the EFDMReg model (see also Figure S23).

Table S16: Data from ZIDM. Summaries of Sensitivity, Specificity, PPV, NPV, and accuracy measures across the 300 replications.

Metric	Model	Min	1st Qu.	Mean	SD	Median	3rd Qu.	Max
Sensitivity	EFDM	0.144	0.162	0.169	0.011	0.169	0.177	0.206
	ZIDM	0.775	0.777	0.778	0.001	0.778	0.779	0.782
Specificity	EFDM	0.828	0.850	0.855	0.008	0.855	0.861	0.875
	ZIDM	0.234	0.236	0.236	0.001	0.236	0.236	0.237
PPV	EFDM	0.185	0.203	0.211	0.011	0.210	0.219	0.250
	ZIDM	0.168	0.183	0.189	0.008	0.189	0.195	0.221
NPV	EFDM	0.790	0.812	0.818	0.008	0.818	0.824	0.839
	ZIDM	0.795	0.817	0.823	0.008	0.823	0.828	0.844
Accuracy	EFDM	0.693	0.720	0.727	0.010	0.728	0.735	0.755
	ZIDM	0.326	0.334	0.337	0.004	0.337	0.340	0.354

Figure S22: Data from ZIDM. Boxplots of the posterior predicted means of sensitivity, specificity, PPV, NPV, and accuracy by model computed on 300 simulated datasets.

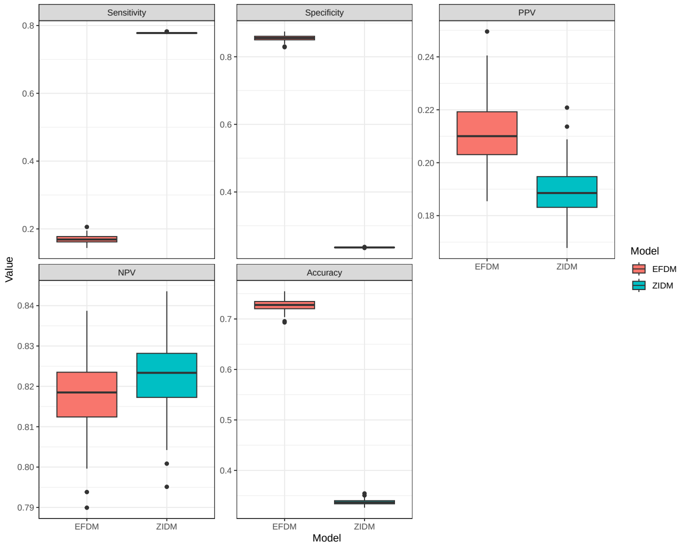

Figure S23: Data from ZIDM. Posterior mean of the average KL divergence for the ZIDM and the EFDMReg models for $\varepsilon = 0.5$ and $\varepsilon = 1$.

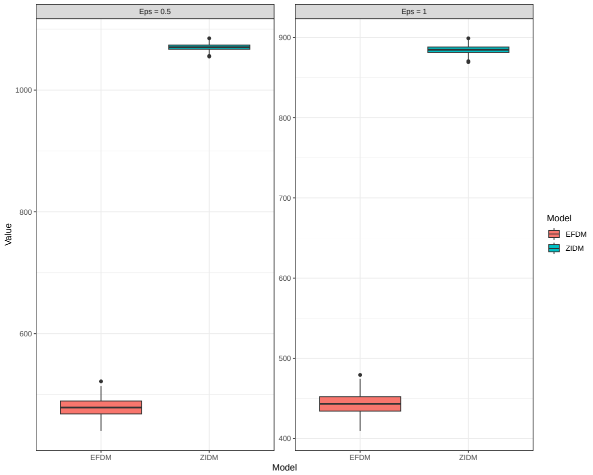

4 Computational aspects

4.1 Instructions for running the EFDMReg Model

This section provides a concise guide for fitting the EFDMReg model using the `rstan` package in `R`. All required Stan models, along with an example of `R` script, are available at the following repository: https://github.com/robertoascari/EFDMReg.

To begin, load the necessary `R` packages and set a few configuration options to optimize Stan's performance:

```
library(loo)
library(rstan)

rstan_options(auto_write = TRUE)
Sys.setenv(LOCAL_CPPFLAGS = '-march=native')
```

Next, specify the path where the Stan model is located and compile it using the `rstan::stan_model()` function:

```
wd_stanmodels <- "inst/stan/"

EFDM <- rstan::stan_model(file = paste0(wd_stanmodels, "EFDM_hyper_w.stan"))
```

We assume that the user has a dataset consisting of a response matrix `Y` containing microbiome compositions on `D` taxa, and a design matrix `X` of dimension `N` × `K`, which includes covariate information. The vector `n`, of length `N`, stores the number of bacterial reads for each statistical unit.

Before fitting the model, we define the MCMC settings such as the number of iterations, chains, and the length of the warmup period. These values are used by the `rstan::sampling()` function to set up the desired MCMC technique.

```
n.iter <- 8000
nchain <- 1
warmup <- 0.5 * n.iter
```

Then, we create a list that contains all the data inputs required by the Stan model, and fit the EFDMReg model using the `rstan::sampling()` function:

```
fit.EFDM <- rstan::sampling(
    object = EFDM,
    data = data.stan,
    iter = n.iter,
    warmup = warmup,
    chains = chain,
    cores = 1,
    thin = 1,
    pars = c("beta_raw", "aplus", "p", "w_norm", "log_lik"),
```

```
    refresh = n.iter / 100
)
```

After the model is fitted, posterior summaries for selected parameters can be obtained using the `summary()` function:

```
rstan::summary(fit.EFDM, pars = c("beta_raw", "aplus", "p", "w_norm"))$summary
```

Alternatively, posterior samples of specific parameters can be extracted and analyzed. For instance, to explore the posterior distribution of α^+ (coded as `aplus`), use the following code:

```
aplus_chain <- rstan::extract(fit.EFDM, pars = "aplus")[[1]]

mean(aplus_chain)
quantile(aplus_chain, probs = c(0.025, 0.975))
hist(aplus_chain, prob = TRUE, main = "Posterior distribution of aplus")
```

This simple workflow allows users to fit the EFDMReg model and perform basic posterior inference. Other details about Stan can be found at the Stan User's Guide and the `rstan` interface page.

4.2 Hyperparameter choices, HMC implementation, computational time, and sensitivity analysis (Section 5 of the paper)

Our general approach in choosing priors favors non- or weakly informative priors to induce the least impact on the posterior distributions.

In the analyses of Section 5 of the main paper, we specified the Gamma($k \cdot g, g$) prior for α^+ by setting $k = 1$ and the rate parameter $g = 0.001$, so that the prior distribution induces a large variability around the prior mean equal to $k = 1$.

The parameters $\tilde{w}_r$ were endowed with a Beta prior with both shape parameters equal to 30. For the variance of the normal prior for the regression coefficients, we selected $\sigma_0^2 = 0.001$ and $\sigma_1^2 = \tau^2 = 25$ as hyperparameters in priors (24) and (25) of the main paper. Finally, for the parameter θ_k in prior (26), we choose a Uniform distribution to consider a non-informative prior.

Moreover, we considered four chains composed of 50.000 iterations with the first half dedicated to the warm-up. The thinning period is set equal to two, and initial values are random. The assessment of the convergence of the chains was performed visually and via the Geweke diagnostic [1].

As for computational times, we ran the EFDMReg model by considering four chains with 50.000 iterations. The time required to fit a chain was 1.58 hours for warm-up and 2.29 hours for sampling when $D = 3$, 5.3 hours for warm-up and 6.28 hours for sampling when $D = 13$. Times are recorded on a PC running Windows 11 with Intel(R) Core(TM) i7-8700T 2.40GHz processor.

To investigate whether and to what extent different choices in the priors affect the posterior distributions of the EFDMReg model's parameters we performed a sensitivity study. More precisely, we report results related to the case with $D = 3$ due to the reasonable number of parameters involved. Nonetheless, analogous remarks hold for larger values of D.

For each parameter, we refer to the priors listed in Section 3.2 of the main paper and consider different values for the corresponding hyperparameters. In particular, we select a diffuse multivariate Normal distribution for $\boldsymbol{\beta}_r$, as described in the paper, and we choose three different values for the common prior variance (i.e., the value on the diagonal of Σ) to account for different amounts of vagueness, namely, 25, 100, and 400. Results are reported in Table S17, which shows the posterior means, medians, and standard deviations for all the elements in $\boldsymbol{\beta}_1$ and $\boldsymbol{\beta}_2$ for different values of σ^2. It is interesting to note that these summary statistics do not significantly change across the three considered scenarios, pointing out that the model can recognize and quantify the effect of each covariate despite the chosen level of vagueness (i.e., prior variance).

Table S17: Sensitivity Analysis. Posterior means, medians, and standard deviations of the regression coefficients for different choices of prior variance σ^2.

		$\sigma^2 = 25$			$\sigma^2 = 100$			$\sigma^2 = 400$		
		Mean	Median	SD	Mean	Median	SD	Mean	Median	SD
Bacteroides	Intercept	2.851	2.850	0.282	2.853	2.855	0.288	2.875	2.878	0.291
	Sex	0.001	0.001	0.276	-0.002	-0.004	0.279	-0.011	-0.005	0.280
	Phosphorous	-0.252	-0.248	0.433	-0.243	-0.244	0.436	-0.234	-0.233	0.436
	Vitamin B12	-0.148	-0.154	0.155	-0.153	-0.157	0.155	-0.157	-0.162	0.154
	Sodium	0.201	0.200	0.176	0.204	0.201	0.181	0.205	0.204	0.181
	Manganese	0.336	0.331	0.219	0.333	0.328	0.220	0.335	0.327	0.224
	Iodine	0.015	0.011	0.147	0.016	0.012	0.145	0.014	0.012	0.145
	Riboflavin B2 w/o vit. pills	-0.279	-0.278	0.204	-0.275	-0.274	0.207	-0.281	-0.282	0.213
	Pyridoxine B6 w/o vit. pills	-0.052	-0.052	0.176	-0.054	-0.056	0.172	-0.057	-0.056	0.175
	Maltose	-0.093	-0.092	0.183	-0.095	-0.096	0.184	-0.088	-0.085	0.182
	Proline	0.277	0.276	0.295	0.268	0.265	0.294	0.265	0.258	0.298
	Choline, Phosphatidylcholine	-0.311	-0.310	0.355	-0.311	-0.308	0.359	-0.317	-0.309	0.362
	Total Choline, no betaine	0.361	0.359	0.459	0.361	0.365	0.465	0.366	0.354	0.466
Prevotella	Intercept	-0.028	-0.015	0.392	-0.056	-0.044	0.405	-0.047	-0.030	0.416
	Sex	-1.981	-1.969	0.504	-1.997	-1.977	0.514	-2.021	-2.003	0.512
	Phosphorous	1.928	1.929	0.700	1.946	1.955	0.705	2.000	2.005	0.706
	Vitamin B12	-2.730	-2.586	1.336	-2.898	-2.703	1.447	-2.941	-2.774	1.466
	Sodium	-0.033	-0.034	0.279	-0.033	-0.041	0.288	-0.035	-0.039	0.279
	Manganese	-0.478	-0.474	0.374	-0.478	-0.472	0.374	-0.500	-0.504	0.380
	Iodine	0.105	0.108	0.224	0.107	0.108	0.223	0.108	0.113	0.221
	Riboflavin B2 w/o vit. pills	-0.806	-0.810	0.353	-0.802	-0.801	0.354	-0.826	-0.833	0.357
	Pyridoxine B6 w/o vit. pills	1.288	1.294	0.296	1.293	1.294	0.294	1.315	1.318	0.298
	Maltose	0.727	0.727	0.301	0.727	0.730	0.305	0.747	0.756	0.299
	Proline	-1.398	-1.395	0.466	-1.419	-1.414	0.466	-1.440	-1.428	0.467
	Choline, Phosphatidylcholin	-0.142	-0.137	0.530	-0.137	-0.131	0.540	-0.132	-0.140	0.538
	Total Choline, no betaine	-1.211	-1.214	0.675	-1.225	-1.222	0.692	-1.240	-1.250	0.686

Regarding the parameter α^+, which is involved in the component-specific precision, we consider a Gamma$(k \cdot g, g)$ distribution, with a rate hyperparameter $g = 0.001$ to induce a large variance around the prior mean k ($k \in \{1, 10, 20\}$). Since the dimension of α^+ does not depend on the number D of taxa considered in the analysis, we expanded the sensitivity analysis by also including the case with $D = 13$ (as in Section 5.2 of the main paper). Results are summarized in Table S18 and Figure S24, which show the posterior means, medians, and standard deviations, as well as the (simulated) posterior distribution of α^+, considering the three different values of k. The posterior distribution of α^+ seems to be robust with respect to the different values selected for the prior mean k.

Table S18: Sensitivity Analysis. Posterior means, medians and standard deviations of the parameter α^+ for different choices of the prior expectation k.

D	k	Mean	Median	SD
3	1	2.704	2.601	0.750
	10	2.664	2.574	0.729
	20	2.717	2.614	0.756
13	1	17.380	17.365	1.098
	10	17.376	17.372	1.088
	20	17.363	17.335	1.105

For the parameter $\mathbf{p}$, to preserve a weakly informative framework, we considered symmetric prior distributions on the simplex. A reasonable family of such distributions is represented by the Dirichlet family with fixed mean vector $(1/D, \ldots, 1/D)^\intercal$ and a varying (prior) precision a. Note that, within this setting, a hyperparameter $a = 1$ leads to a uniform distribution on the simplex. Table S19 shows the posterior means, medians and standard deviations of $\mathbf{p}$. Considering an unbounded prior distribution (i.e., $a = 0.1$), allows us to obtain comparable posterior means and medians for the mixing weights, with an (ignorable) increase in the standard deviation. Contrarily, increasing the prior precision (i.e., $a = 2$) has an impact on the posterior means, which are more shifted towards the center of the simplex. Nonetheless, noting that the posterior medians experience a lower impact, we may conclude that increasing the hyperparameter a has the consequence of producing an asymmetric posterior for the mixing weights. Thus, in such cases, one should prefer the posterior medians as estimates.

Finally, we select a prior distribution for each element $\tilde{w}_r$ in $\tilde{\mathbf{w}}$. Recalling that $\tilde{w}_r \in (0, 1)$, a reasonable choice is to consider a Beta(δ_1, δ_2), with $\delta_1 = \delta_2 = \delta$ to allow for a symmetric prior with mean set in the middle of the parameter space. In the sensitivity analysis, we consider different values of δ (thus modifying the prior variability), namely 5, 10, and 30. Results are reported in Table S20 and show a limited impact of the choice of the hyperparameter δ on the posterior.

Figure S24: Sensitivity Analysis. Posterior distribution of the parameter α^+ for the COMBO application with $D = 3$ (top panel) and $D = 13$ (bottom panel).

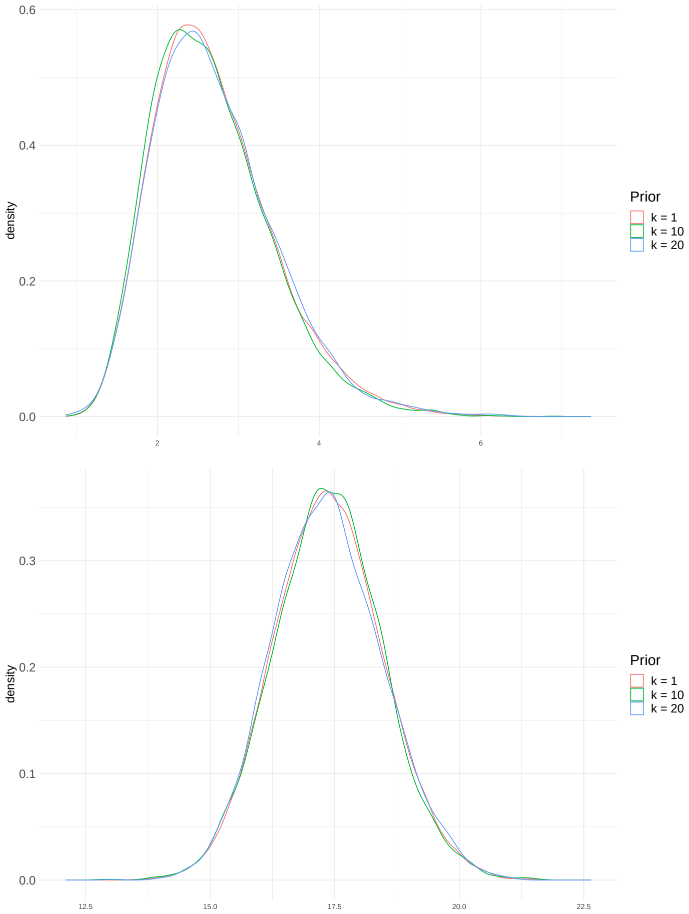

Table S19: Sensitivity Analysis. Posterior means, medians and standard deviations of the parameter $\mathbf{p}$ for different choices of the prior precision parameter a.

	p_1			p_2			p_3		
a	Mean	Median	SD	Mean	Median	SD	Mean	Median	SD
0.1	0.854	0.875	0.091	0.145	0.124	0.091	0.001	0	0.007
1	0.824	0.827	0.035	0.170	0.167	0.035	0.006	0.005	0.005
2	0.656	0.738	0.241	0.149	0.128	0.085	0.195	0.009	0.255

Table S20: Sensitivity Analysis. Posterior means, medians, and standard deviations of the parameter $\tilde{\mathbf{w}}$ for different choices of the prior precision parameter δ.

	$\tilde{w}_1$			$\tilde{w}_2$			$\tilde{w}_3$		
δ	Mean	Median	SD	Mean	Median	SD	Mean	Median	SD
5	0.489	0.491	0.135	0.797	0.802	0.049	0.323	0.305	0.132
10	0.501	0.503	0.102	0.754	0.762	0.059	0.392	0.383	0.107
30	0.514	0.514	0.062	0.612	0.614	0.067	0.474	0.473	0.067

5 COMBO application (Section 5 of the paper)

5.1 Correlation estimation (Section 5.1 of the paper)

5.1.1 Correlation behavior for increasing bacterial reads

The correlations between bacterial counts depend on the reads n_i's, which vary across samples. Therefore, to estimate these correlations we used the convergence result provided in Section 2 of the paper. Specifically, as $n_i \to \infty$, $\mathbf{Y}_i/n_i \xrightarrow{a.s.} \mathbf{\Pi}_i$ where $\mathbf{\Pi}_i$ represents the proportion vector. This is well-suited given the observed range of n_i, which spans from 500 to 13,760 with a mean value of 6359. For a validation of the convergence and its implications for correlation estimation, we provide a numerical investigation. Specifically, we report some plots concerning the EFDM estimated correlation among pairs of bacteria as a function of the total number of bacterial reads n (the abbreviations used for each taxon are reported in Table S21). Figure S25 clearly validates the expected behavior of correlations as n_i increases. Therefore, the EFDM correlation matrix was estimated by averaging over MCMC replications using a representative value of n_i (mean, median, or maximum of the observed values resulting in consistent outcomes).

Table S21: COMBO dataset. Counts and sample proportions of zero reads for each taxon.

Taxa	Abbreviation	Zero Counts	Zero Prop.
Bacteroides	Bct	0	0.0000
Prevotella	Prv	60	0.6250
Ruminococcus	Rmn	25	0.2604
Parasutterella	Prs	24	0.2500
Oscillibacter	Osc	2	0.0208
Subdoligranulum	Sbd	11	0.1146
Faecalibacterium	Fcl	4	0.0417
Parabacteroides	Prb	4	0.0417
Alistipes	Als	6	0.0625
Odoribacter	Odr	21	0.2188
Barnesiella	Brn	39	0.4062
Phascolarctobacterium	Phs	45	0.4688
Coprococcus	Cpr	9	0.0938

5.1.2 EFDM fitted correlation matrix

In this section, we report the taxa correlation matrix estimated under the EFDM model. In particular, Table S22 reports the posterior means and standard deviations of each estimated correlation. When interpreting these results, it is crucial to recognize that, due to the summation constraint on the counts, a more appropriate benchmark for the absence of association is a negative value rather than

Figure S25: COMBO application. Correlation coefficients under the EFDM model as a function of the number of bacterial reads. Lower panels refer to obese (left) and lean (right) individuals.

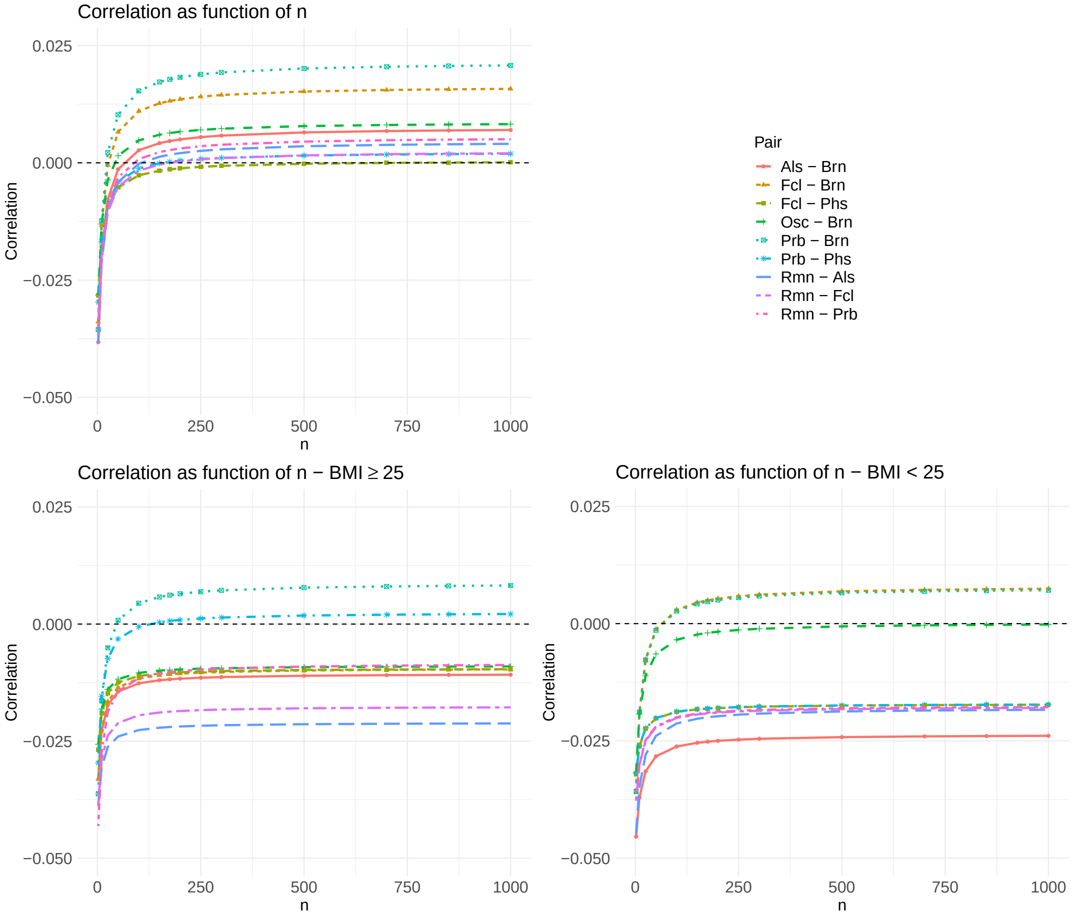

zero. In fact, the sum of the covariances of each taxon with all other taxa is negative, implying that, for example, not all taxa can be positively associated. For instance, consider the Dirichlet-Multinomial (DM) model. This model represents a prototypical framework for the absence of associations, as it is based on the multinomial distribution, which assumes independent components, and the Dirichlet distribution, which, among distributions on the simplex, uniquely accounts for all forms of compositional independence (i.e., independence that is compatible with the simplex constraint). Here the correlations are always negative and given by:

$$-\sqrt{\frac{\mu_r}{1-\mu_r}\frac{\mu_{r'}}{1-\mu_{r'}}}.$$

The robustness of the identified correlations has been assessed through bootstrap. The values of the 90% bootstrap confidence intervals (CI) reported in Table S23 are computed on 300 replications, and point out that all EFDM-based estimates are contained in the intervals. Moreover, all correlations identified as non-null at 90% level by the EFDM model (see Table S22) show a bootstrap CI that does not contain the zero value.

Tables S24 and S25 report the estimated correlation matrices under the EFDM model for obese (BMI ≥ 25) and lean (BMI < 25) subjects respectively.

Table S22: COMBO application. Posterior mean and standard deviation (in parenthesis) for each correlation coefficient estimated under the EFDMReg model.

	Bct	Prv	Rmn	Prs	Osc	Sbd	Fcl	Prb	Als	Odr	Brn	Phs	Cpr
Bct	1.0000	-0.2804	-0.1923	-0.1835	-0.2535	-0.2668	-0.2988	-0.3105	-0.3271	-0.2035	-0.1973	-0.1554	-0.2125
	(0.000)	*(0.038)*	*(0.039)*	*(0.044)*	*(0.042)*	*(0.049)*	*(0.042)*	*(0.043)*	*(0.046)*	*(0.043)*	*(0.038)*	*(0.036)*	*(0.044)*
Prv	-0.2804	1.0000	-0.0437	-0.0323	-0.0518	-0.0457	-0.0636	-0.0677	-0.0722	-0.0423	-0.0496	-0.0354	-0.0398
	(0.038)	*(0.000)*	*(0.011)*	*(0.006)*	*(0.009)*	*(0.008)*	*(0.011)*	*(0.012)*	*(0.014)*	*(0.011)*	*(0.009)*	*(0.009)*	*(0.007)*
Rmn	-0.1923	-0.0437	1.0000	-0.0108	-0.0015	-0.0167	0.0024	0.0055	0.0045	-0.0049	-0.0245	-0.0148	-0.0075
	(0.039)	*(0.011)*	*(0.000)*	*(0.017)*	*(0.025)*	*(0.020)*	*(0.029)*	*(0.032)*	*(0.033)*	*(0.021)*	*(0.023)*	*(0.015)*	*(0.020)*
Prs	-0.1835	-0.0323	-0.0108	1.0000	-0.0189	-0.0234	-0.0214	-0.0216	-0.0224	-0.0148	-0.0090	-0.0099	-0.0173
	(0.044)	*(0.006)*	*(0.017)*	*(0.000)*	*(0.014)*	*(0.012)*	*(0.017)*	*(0.018)*	*(0.021)*	*(0.014)*	*(0.020)*	*(0.013)*	*(0.011)*
Osc	-0.2535	-0.0518	-0.0015	-0.0189	1.0000	-0.0293	-0.0208	-0.0202	-0.0181	-0.0122	0.0086	-0.0028	-0.0201
	(0.042)	*(0.009)*	*(0.025)*	*(0.014)*	*(0.000)*	*(0.017)*	*(0.020)*	*(0.021)*	*(0.026)*	*(0.022)*	*(0.022)*	*(0.019)*	*(0.015)*
Sbd	-0.2668	-0.0457	-0.0167	-0.0234	-0.0293	1.0000	-0.0334	-0.0340	-0.0352	-0.0228	-0.0141	-0.0151	-0.0261
	(0.049)	*(0.008)*	*(0.020)*	*(0.012)*	*(0.017)*	*(0.000)*	*(0.020)*	*(0.021)*	*(0.024)*	*(0.017)*	*(0.023)*	*(0.016)*	*(0.014)*
Fcl	-0.2988	-0.0636	0.0024	-0.0214	-0.0208	-0.0334	1.0000	-0.0200	-0.0168	-0.0118	0.0163	0.0004	-0.0221
	(0.042)	*(0.011)*	*(0.029)*	*(0.017)*	*(0.020)*	*(0.020)*	*(0.000)*	*(0.024)*	*(0.029)*	*(0.026)*	*(0.023)*	*(0.023)*	*(0.018)*
Prb	-0.3105	-0.0677	0.0055	-0.0216	-0.0202	-0.0340	-0.0200	1.0000	-0.0146	-0.0105	0.0214	0.0023	-0.0222
	(0.043)	*(0.012)*	*(0.032)*	*(0.018)*	*(0.021)*	*(0.021)*	*(0.024)*	*(0.000)*	*(0.031)*	*(0.028)*	*(0.024)*	*(0.024)*	*(0.019)*
Als	-0.3271	-0.0722	0.0045	-0.0224	-0.0181	-0.0352	-0.0168	-0.0146	1.0000	-0.0108	0.0075	-0.0026	-0.0217
	(0.046)	*(0.014)*	*(0.033)*	*(0.021)*	*(0.026)*	*(0.024)*	*(0.029)*	*(0.031)*	*(0.000)*	*(0.029)*	*(0.038)*	*(0.027)*	*(0.023)*
Odr	-0.2035	-0.0423	-0.0049	-0.0148	-0.0122	-0.0228	-0.0118	-0.0105	-0.0108	1.0000	-0.0117	-0.0108	-0.0142
	(0.043)	*(0.011)*	*(0.021)*	*(0.014)*	*(0.022)*	*(0.017)*	*(0.026)*	*(0.028)*	*(0.029)*	*(0.000)*	*(0.026)*	*(0.016)*	*(0.017)*
Brn	-0.1973	-0.0496	-0.0245	-0.0090	0.0086	-0.0141	0.0163	0.0214	0.0075	-0.0117	1.0000	-0.0254	-0.0021
	(0.038)	*(0.009)*	*(0.023)*	*(0.020)*	*(0.022)*	*(0.023)*	*(0.023)*	*(0.024)*	*(0.038)*	*(0.026)*	*(0.000)*	*(0.018)*	*(0.021)*
Phs	-0.1554	-0.0354	-0.0148	-0.0099	-0.0028	-0.0151	0.0004	0.0023	-0.0026	-0.0108	-0.0254	1.0000	-0.0074
	(0.036)	*(0.009)*	*(0.015)*	*(0.013)*	*(0.019)*	*(0.016)*	*(0.023)*	*(0.024)*	*(0.027)*	*(0.016)*	*(0.018)*	*(0.000)*	*(0.015)*
Cpr	-0.2125	-0.0398	-0.0075	-0.0173	-0.0201	-0.0261	-0.0221	-0.0222	-0.0217	-0.0142	-0.0021	-0.0074	1.0000
	(0.044)	*(0.007)*	*(0.020)*	*(0.011)*	*(0.015)*	*(0.014)*	*(0.018)*	*(0.019)*	*(0.023)*	*(0.017)*	*(0.021)*	*(0.015)*	*(0.000)*

Table S23: COMBO application. EFDM Correlation matrix. Bootstrap 90% CI and original estimate (i.e., the EFDM's correlation coefficient estimated on the original sample).

	Bct	Prv	Rmn	Prs	Osc	Sbd	Fcl
Bct	(1 ; 1)<br>1	(-0.342 ; -0.221)<br>-0.2804	(-0.225 ; -0.158)<br>-0.1923	(-0.201 ; -0.162)<br>-0.1835	(-0.28 ; -0.227)<br>-0.2535	(-0.327 ; -0.221)<br>-0.2668	(-0.33 ; -0.262)<br>-0.2988
Prv	(-0.342 ; -0.221)<br>-0.2804	(1 ; 1)<br>1	(-0.057 ; -0.027)<br>-0.0437	(-0.043 ; -0.023)<br>-0.0323	(-0.069 ; -0.035)<br>-0.0518	(-0.061 ; -0.031)<br>-0.0457	(-0.083 ; -0.043)<br>-0.0636
Rmn	(-0.225 ; -0.158)<br>-0.1923	(-0.057 ; -0.027)<br>-0.0437	(1 ; 1)<br>1	(-0.019 ; -0.001)<br>-0.0108	(-0.025 ; 0.019)<br>-0.0015	(-0.035 ; 0.004)<br>-0.0167	(-0.028 ; 0.028)<br>0.0024
Prs	(-0.201 ; -0.162)<br>-0.1835	(-0.043 ; -0.023)<br>-0.0323	(-0.019 ; -0.001)<br>-0.0108	(1 ; 1)<br>1	(-0.026 ; -0.006)<br>-0.0189	(-0.032 ; -0.012)<br>-0.0234	(-0.029 ; -0.005)<br>-0.0214
Osc	(-0.28 ; -0.227)<br>-0.2535	(-0.069 ; -0.035)<br>-0.0518	(-0.025 ; 0.019)<br>-0.0015	(-0.026 ; -0.006)<br>-0.0189	(1 ; 1)<br>1	(-0.044 ; -0.012)<br>-0.0293	(-0.034 ; -0.004)<br>-0.0208
Sbd	(-0.327 ; -0.221)<br>-0.2668	(-0.061 ; -0.031)<br>-0.0457	(-0.035 ; 0.004)<br>-0.0167	(-0.032 ; -0.012)<br>-0.0234	(-0.044 ; -0.012)<br>-0.0293	(1 ; 1)<br>1	(-0.054 ; -0.013)<br>-0.0334
Fcl	(-0.33 ; -0.262)<br>-0.2988	(-0.083 ; -0.043)<br>-0.0636	(-0.028 ; 0.028)<br>0.0024	(-0.029 ; -0.005)<br>-0.0214	(-0.034 ; -0.004)<br>-0.0208	(-0.054 ; -0.013)<br>-0.0334	(1 ; 1)<br>1
Prb	(-0.35 ; -0.271)<br>-0.3105	(-0.09 ; -0.047)<br>-0.0677	(-0.029 ; 0.036)<br>0.0055	(-0.031 ; -0.004)<br>-0.0216	(-0.034 ; -0.002)<br>-0.0202	(-0.055 ; -0.012)<br>-0.034	(-0.036 ; 0.002)<br>-0.02
Als	(-0.375 ; -0.283)<br>-0.3271	(-0.104 ; -0.049)<br>-0.0722	(-0.03 ; 0.033)<br>0.0045	(-0.034 ; -0.005)<br>-0.0224	(-0.035 ; 0.012)<br>-0.0181	(-0.063 ; 0.001)<br>-0.0352	(-0.039 ; 0.026)<br>-0.0168
Odr	(-0.226 ; -0.181)<br>-0.2035	(-0.057 ; -0.027)<br>-0.0423	(-0.021 ; 0.007)<br>-0.0049	(-0.021 ; -0.006)<br>-0.0148	(-0.027 ; 0.009)<br>-0.0122	(-0.036 ; -0.004)<br>-0.0228	(-0.031 ; 0.015)<br>-0.0118
Brn	(-0.262 ; -0.135)<br>-0.1973	(-0.065 ; -0.034)<br>-0.0496	(-0.046 ; 0.003)<br>-0.0245	(-0.021 ; 0.003)<br>-0.009	(-0.006 ; 0.029)<br>0.0086	(-0.04 ; 0.014)<br>-0.0141	(-0.006 ; 0.045)<br>0.0163
Phs	(-0.186 ; -0.11)<br>-0.1554	(-0.047 ; -0.023)<br>-0.0354	(-0.027 ; -0.005)<br>-0.0148	(-0.016 ; -0.003)<br>-0.0099	(-0.016 ; 0.01)<br>-0.0028	(-0.029 ; -0.001)<br>-0.0151	(-0.018 ; 0.017)<br>0.0004
Cpr	(-0.23 ; -0.192)<br>-0.2125	(-0.052 ; -0.027)<br>-0.0398	(-0.022 ; 0.006)<br>-0.0075	(-0.022 ; -0.009)<br>-0.0173	(-0.029 ; -0.01)<br>-0.0201	(-0.037 ; -0.013)<br>-0.0261	(-0.032 ; -0.01)<br>-0.0221

X.	Prb	Als	Odr	Brn	Phs	Cpr
Bct	(-0.35 ; -0.271)<br>-0.3105	(-0.375 ; -0.283)<br>-0.3271	(-0.226 ; -0.181)<br>-0.2035	(-0.262 ; -0.135)<br>-0.1973	(-0.186 ; -0.11)<br>-0.1554	(-0.23 ; -0.192)<br>-0.2125
Prv	(-0.09 ; -0.047)<br>-0.0677	(-0.104 ; -0.049)<br>-0.0722	(-0.057 ; -0.027)<br>-0.0423	(-0.065 ; -0.034)<br>-0.0496	(-0.047 ; -0.023)<br>-0.0354	(-0.052 ; -0.027)<br>-0.0398
Rmn	(-0.029 ; 0.036)<br>0.0055	(-0.03 ; 0.033)<br>0.0045	(-0.021 ; 0.007)<br>-0.0049	(-0.046 ; 0.003)<br>-0.0245	(-0.027 ; -0.005)<br>-0.0148	(-0.022 ; 0.006)<br>-0.0075
Prs	(-0.031 ; -0.004)<br>-0.0216	(-0.034 ; -0.005)<br>-0.0224	(-0.021 ; -0.006)<br>-0.0148	(-0.021 ; 0.003)<br>-0.009	(-0.016 ; -0.003)<br>-0.0099	(-0.022 ; -0.009)<br>-0.0173
Osc	(-0.034 ; -0.002)<br>-0.0202	(-0.035 ; 0.012)<br>-0.0181	(-0.027 ; 0.009)<br>-0.0122	(-0.006 ; 0.029)<br>0.0086	(-0.016 ; 0.01)<br>-0.0028	(-0.029 ; -0.01)<br>-0.0201
Sbd	(-0.055 ; -0.012)<br>-0.034	(-0.063 ; 0.001)<br>-0.0352	(-0.036 ; -0.004)<br>-0.0228	(-0.04 ; 0.014)<br>-0.0141	(-0.029 ; -0.001)<br>-0.0151	(-0.037 ; -0.013)<br>-0.0261
Fcl	(-0.036 ; 0.002)<br>-0.02	(-0.039 ; 0.026)<br>-0.0168	(-0.031 ; 0.015)<br>-0.0118	(-0.006 ; 0.045)<br>0.0163	(-0.018 ; 0.017)<br>0.0004	(-0.032 ; -0.01)<br>-0.0221
Prb	(1 ; 1)<br>1	(-0.034 ; 0.03)<br>-0.0146	(-0.031 ; 0.018)<br>-0.0105	(-0.007 ; 0.054)<br>0.0214	(-0.021 ; 0.021)<br>0.0023	(-0.033 ; -0.009)<br>-0.0222
Als	(-0.034 ; 0.03)<br>-0.0146	(1 ; 1)<br>1	(-0.031 ; 0.018)<br>-0.0108	(-0.079 ; 0.053)<br>0.0075	(-0.039 ; 0.017)<br>-0.0026	(-0.034 ; -0.004)<br>-0.0217
Odr	(-0.031 ; 0.018)<br>-0.0105	(-0.031 ; 0.018)<br>-0.0108	(1 ; 1)<br>1	(-0.031 ; 0.013)<br>-0.0117	(-0.018 ; 0.0001)<br>-0.0108	(-0.024 ; -0.001)<br>-0.0142
Brn	(-0.007 ; 0.054)<br>0.0214	(-0.079 ; 0.053)<br>0.0075	(-0.031 ; 0.013)<br>-0.0117	(1 ; 1)<br>1	(-0.045 ; -0.004)<br>-0.0254	(-0.013 ; 0.013)<br>-0.0021
Phs	(-0.021 ; 0.021)<br>0.0023	(-0.039 ; 0.017)<br>-0.0026	(-0.018 ; 0.0001)<br>-0.0108	(-0.045 ; -0.004)<br>-0.0254	(1 ; 1)<br>1	(-0.016 ; 0.002)<br>-0.0074
Cpr	(-0.033 ; -0.009)<br>-0.0222	(-0.034 ; -0.004)<br>-0.0217	(-0.024 ; -0.001)<br>-0.0142	(-0.013 ; 0.013)<br>-0.0021	(-0.016 ; 0.002)<br>-0.0074	(1 ; 1)<br>1

Table S24: COMBO application. Estimated correlation matrix among $D = 13$ bacterial taxa for subjects with BMI ≥ 25 under the EFDMReg model.

	Bct	Prv	Rmn	Prs	Osc	Sbd	Fcl	Prb	Als	Odr	Brn	Phs	Cpr
Bct	1.0000	-0.2295	-0.1666	-0.1715	-0.2274	-0.2716	-0.2736	-0.3010	-0.2897	-0.2022	-0.1348	-0.1098	-0.2084
Prv	-0.2295	1.0000	-0.0366	-0.0319	-0.0426	-0.0439	-0.0571	-0.0644	-0.0552	-0.0372	-0.0349	-0.0276	-0.0374
Rmn	-0.1666	-0.0366	1.0000	-0.0197	-0.0170	-0.0315	-0.0176	-0.0084	-0.0211	-0.0190	-0.0201	-0.0165	-0.0192
Prs	-0.1715	-0.0319	-0.0197	1.0000	-0.0233	-0.0326	-0.0271	-0.0252	-0.0299	-0.0224	-0.0173	-0.0144	-0.0228
Osc	-0.2274	-0.0426	-0.0170	-0.0233	1.0000	-0.0420	-0.0363	-0.0401	-0.0412	-0.0289	-0.0089	-0.0091	-0.0301
Sbd	-0.2716	-0.0439	-0.0315	-0.0326	-0.0420	1.0000	-0.0516	-0.0544	-0.0537	-0.0380	-0.0263	-0.0219	-0.0387
Fcl	-0.2736	-0.0571	-0.0176	-0.0271	-0.0363	-0.0516	1.0000	-0.0391	-0.0466	-0.0333	-0.0095	-0.0095	-0.0346
Prb	-0.3010	-0.0644	-0.0084	-0.0252	-0.0401	-0.0544	-0.0391	1.0000	-0.0502	-0.0347	0.0087	0.0024	-0.0370
Als	-0.2897	-0.0552	-0.0211	-0.0299	-0.0412	-0.0537	-0.0466	-0.0502	1.0000	-0.0369	-0.0106	-0.0116	-0.0383
Odr	-0.2022	-0.0372	-0.0190	-0.0224	-0.0289	-0.0380	-0.0333	-0.0347	-0.0369	1.0000	-0.0137	-0.0123	-0.0273
Brn	-0.1348	-0.0349	-0.0201	-0.0173	-0.0089	-0.0263	-0.0095	0.0087	-0.0106	-0.0137	1.0000	-0.0174	-0.0134
Phs	-0.1098	-0.0276	-0.0165	-0.0144	-0.0091	-0.0219	-0.0095	0.0024	-0.0116	-0.0123	-0.0174	1.0000	-0.0120
Cpr	-0.2084	-0.0374	-0.0192	-0.0228	-0.0301	-0.0387	-0.0346	-0.0370	-0.0383	-0.0273	-0.0134	-0.0120	1.0000

Table S25: COMBO application. Estimated correlation matrix among $D = 13$ bacterial taxa for subjects with BMI < 25 under the EFDMReg model.

	Bct	Prv	Rmn	Prs	Osc	Sbd	Fcl	Prb	Als	Odr	Brn	Phs	Cpr
Bct	1.0000	-0.2295	-0.1854	-0.1840	-0.2640	-0.2320	-0.2937	-0.2921	-0.3228	-0.1923	-0.1589	-0.1585	-0.2153
Prv	-0.2295	1.0000	-0.0353	-0.0291	-0.0473	-0.0393	-0.0548	-0.0544	-0.0725	-0.0378	-0.0448	-0.0298	-0.0345
Rmn	-0.1854	-0.0353	1.0000	-0.0207	-0.0184	-0.0195	-0.0177	-0.0176	-0.0180	-0.0188	-0.0276	-0.0206	-0.0208
Prs	-0.1840	-0.0291	-0.0207	1.0000	-0.0259	-0.0239	-0.0278	-0.0277	-0.0329	-0.0213	-0.0203	-0.0185	-0.0231
Osc	-0.2640	-0.0473	-0.0184	-0.0259	1.0000	-0.0298	-0.0319	-0.0318	-0.0226	-0.0183	0.0002	-0.0176	-0.0298
Sbd	-0.2320	-0.0393	-0.0195	-0.0239	-0.0298	1.0000	-0.0315	-0.0313	-0.0279	-0.0195	-0.0082	-0.0180	-0.0274
Fcl	-0.2937	-0.0548	-0.0177	-0.0278	-0.0319	-0.0315	1.0000	-0.0321	-0.0178	-0.0167	0.0079	-0.0171	-0.0321
Prb	-0.2921	-0.0544	-0.0176	-0.0277	-0.0318	-0.0313	-0.0321	1.0000	-0.0182	-0.0169	0.0076	-0.0171	-0.0320
Als	-0.3228	-0.0725	-0.0180	-0.0329	-0.0226	-0.0279	-0.0178	-0.0182	1.0000	-0.0150	-0.0237	-0.0255	-0.0331
Odr	-0.1923	-0.0378	-0.0188	-0.0213	-0.0183	-0.0195	-0.0167	-0.0169	-0.0150	1.0000	-0.0267	-0.0212	-0.0212
Brn	-0.1589	-0.0448	-0.0276	-0.0203	0.0002	-0.0082	0.0079	0.0076	-0.0237	-0.0267	1.0000	-0.0281	-0.0141
Phs	-0.1585	-0.0298	-0.0206	-0.0185	-0.0176	-0.0180	-0.0171	-0.0171	-0.0255	-0.0212	-0.0281	1.0000	-0.0187
Cpr	-0.2153	-0.0345	-0.0208	-0.0231	-0.0298	-0.0274	-0.0321	-0.0320	-0.0331	-0.0212	-0.0141	-0.0187	1.0000

5.1.3 Intra- and interclass correlation coefficients with $D = 13$

In this Section, we report the estimates of the intraclass correlation coefficients (Table S26) and of the interclass correlation coefficients (Tables S27 and S28) computed under the DM and the EFDM models.

Table S26: COMBO application. Intraclass correlation coefficients from the DMReg (bold) and EFDMReg models.

**DM**	Bct	Prv	Rmn	Prs	Osc	Sbd
**0.10**	0.17	0.45	0.11	0.20	0.11	0.16
Fcl	Prb	Als	Odr	Brn	Phs	Cpr
0.10	0.10	0.16	0.13	0.10	0.10	0.14

Table S27: COMBO application. Interclass correlation coefficients from the DMReg model.

	Bct	Prv	Rmn	Prs	Osc	Sbd	Fcl	Prb	Als	Odr	Brn	Phs	Cpr
Bct		-0.0109	-0.0173	-0.0161	-0.0252	-0.0225	-0.0298	-0.0313	-0.0319	-0.0190	-0.0155	-0.0134	-0.0200
Prv	-0.0109		-0.0016	-0.0015	-0.0024	-0.0021	-0.0028	-0.0030	-0.0030	-0.0018	-0.0015	-0.0013	-0.0019
Rmn	-0.0173	-0.0016		-0.0024	-0.0038	-0.0034	-0.0045	-0.0047	-0.0048	-0.0029	-0.0023	-0.0020	-0.0030
Prs	-0.0161	-0.0015	-0.0024		-0.0035	-0.0031	-0.0042	-0.0044	-0.0045	-0.0027	-0.0022	-0.0019	-0.0028
Osc	-0.0252	-0.0024	-0.0038	-0.0035		-0.0049	-0.0065	-0.0069	-0.0070	-0.0042	-0.0034	-0.0029	-0.0044
Sbd	-0.0225	-0.0021	-0.0034	-0.0031	-0.0049		-0.0058	-0.0061	-0.0062	-0.0037	-0.0030	-0.0026	-0.0039
Fcl	-0.0298	-0.0028	-0.0045	-0.0042	-0.0065	-0.0058		-0.0081	-0.0083	-0.0049	-0.0040	-0.0035	-0.0052
Prb	-0.0313	-0.0030	-0.0047	-0.0044	-0.0069	-0.0061	-0.0081		-0.0087	-0.0052	-0.0042	-0.0037	-0.0054
Als	-0.0319	-0.0030	-0.0048	-0.0045	-0.0070	-0.0062	-0.0083	-0.0087		-0.0053	-0.0043	-0.0037	-0.0055
Odr	-0.0190	-0.0018	-0.0029	-0.0027	-0.0042	-0.0037	-0.0049	-0.0052	-0.0053		-0.0026	-0.0022	-0.0033
Brn	-0.0155	-0.0015	-0.0023	-0.0022	-0.0034	-0.0030	-0.0040	-0.0042	-0.0043	-0.0026		-0.0018	-0.0027
Phs	-0.0134	-0.0013	-0.0020	-0.0019	-0.0029	-0.0026	-0.0035	-0.0037	-0.0037	-0.0022	-0.0018		-0.0023
Cpr	-0.0200	-0.0019	-0.0030	-0.0028	-0.0044	-0.0039	-0.0052	-0.0054	-0.0055	-0.0033	-0.0027	-0.0023	

Table S28: COMBO application. Interclass correlation coefficients from the EFDMReg model.

	Bct	Prv	Rmn	Prs	Osc	Sbd	Fcl	Prb	Als	Odr	Brn	Phs	Cpr
Bct		-0.0735	-0.0218	-0.0347	-0.0334	-0.0426	-0.0379	-0.0385	-0.0622	-0.0274	-0.0204	-0.0168	-0.0313
Prv	-0.0735		-0.0098	-0.0088	-0.0110	-0.0118	-0.0130	-0.0136	-0.0198	-0.0108	-0.0102	-0.0079	-0.0093
Rmn	-0.0218	-0.0098		-0.0026	0.0005	-0.0021	0.0012	0.0017	-0.0074	-0.0036	-0.0035	-0.0023	-0.0007
Prs	-0.0347	-0.0088	-0.0026		-0.0038	-0.0050	-0.0043	-0.0044	-0.0074	-0.0033	-0.0025	-0.0020	-0.0036
Osc	-0.0334	-0.0110	0.0005	-0.0038		-0.0043	-0.0028	-0.0027	-0.0039	-0.0003	0.0013	0.0006	-0.0029
Sbd	-0.0426	-0.0118	-0.0021	-0.0050	-0.0043		-0.0048	-0.0048	-0.0080	-0.0030	-0.0017	-0.0016	-0.0042
Fcl	-0.0379	-0.0130	0.0012	-0.0043	-0.0028	-0.0048		-0.0027	-0.0037	0.0003	0.0022	0.0012	-0.0032
Prb	-0.0385	-0.0136	0.0017	-0.0044	-0.0027	-0.0048	-0.0027		-0.0032	0.0007	0.0028	0.0016	-0.0031
Als	-0.0622	-0.0198	-0.0074	-0.0074	-0.0039	-0.0080	-0.0037	-0.0032		-0.0088	-0.0082	-0.0058	-0.0049
Odr	-0.0274	-0.0108	-0.0036	-0.0033	-0.0003	-0.0030	0.0003	0.0007	-0.0088		-0.0042	-0.0028	-0.0014
Brn	-0.0204	-0.0102	-0.0035	-0.0025	0.0013	-0.0017	0.0022	0.0028	-0.0082	-0.0042		-0.0028	-0.0003
Phs	-0.0168	-0.0079	-0.0023	-0.0020	0.0006	-0.0016	0.0012	0.0016	-0.0058	-0.0028	-0.0028		-0.0004
Cpr	-0.0313	-0.0093	-0.0007	-0.0036	-0.0029	-0.0042	-0.0032	-0.0031	-0.0049	-0.0014	-0.0003	-0.0004	

5.2 Estimation of regression models (Section 5.2 of the paper)

The variable selection (VS) procedure outlined in Section 3.3 of the main paper was applied to identify the key covariates, among the 120 available in the dataset, that influence the response vector. A covariate was deemed significant if the posterior mean of the probability θ_k (as defined in formula (26) of the main paper) exceeded a threshold of 0.1. This procedure resulted in the selection of 12 significant covariates. It is worth noting that, despite the substantial reduction in the number of covariates, the increase in the WAIC value for the EFDMReg model was relatively modest, rising from 12,269.7 to 12,484.4.

5.2.1 Estimation of EFDMReg, FDMReg, and DMReg models

This section contains the results concerning the EFDMReg, flexible Dirichlet-multinomial regression (FDMReg), and DMReg models fitted considering the covariates selected by the VS approach on the EFDMReg model for all the individuals (Tables S29, S30, and S31). Furthermore, results regarding the EFDMReg model fitted on the subgroup with BMI ≥ 25 (Table S32) and the subgroup with BMI < 25 (Table S33) are also reported.

Table S29: COMBO application. Posterior means and 90% CSs for the regression coefficients of the EFDMReg model.

	Bct	Prv	Rmn	Prs	Osc	Sbd
Intercept	3.299	-0.484	-0.196	-0.415	0.455	0.279
	(2.948, 3.619)	(-1.075, 0.09)	(-0.765, 0.36)	(-0.948, 0.087)	(0.039, 0.837)	(-0.186, 0.758)
Sex	0.081	-1.341	0.103	0.158	0.156	0.135
	(-0.263, 0.421)	(-1.906, -0.849)	(-0.442, 0.643)	(-0.403, 0.726)	(-0.272, 0.591)	(-0.313, 0.587)
Phosphorous	0.292	1.117	0.512	-0.517	0.338	0.123
	(-0.247, 0.821)	(0.234, 2.49)	(-0.328, 1.361)	(-1.354, 0.333)	(-0.349, 1.019)	(-0.584, 0.821)
Vitamin B12	0.183	-3.879	0.319	0.259	0.185	0.234
	(-0.086, 0.532)	(-5.78, -1.659)	(-0.037, 0.719)	(-0.135, 0.684)	(-0.147, 0.568)	(-0.088, 0.62)
Sodium	0.131	-0.231	-0.099	0.131	-0.117	-0.094
	(-0.08, 0.345)	(-0.544, 0.085)	(-0.456, 0.263)	(-0.202, 0.468)	(-0.392, 0.16)	(-0.362, 0.173)
Manganese	0.049	-0.109	-0.283	0.083	-0.036	0.009
	(-0.239, 0.349)	(-0.689, 0.338)	(-0.732, 0.159)	(-0.351, 0.51)	(-0.388, 0.324)	(-0.342, 0.368)
Iodine	-0.007	0.168	-0.025	0.103	0.053	-0.02
	(-0.196, 0.191)	(-0.057, 0.399)	(-0.332, 0.271)	(-0.199, 0.398)	(-0.189, 0.295)	(-0.269, 0.232)
Riboflavin B2 w/o vit. pills	-0.244	-1.013	0.129	-0.02	0.054	-0.117
	(-0.498, 0.014)	(-1.389, -0.598)	(-0.261, 0.524)	(-0.431, 0.393)	(-0.274, 0.386)	(-0.464, 0.236)
Pyridoxine B6 w/o vit. pills	-0.178	0.896	-0.209	-0.056	-0.209	-0.233
	(-0.386, 0.046)	(0.588, 1.246)	(-0.52, 0.101)	(-0.376, 0.256)	(-0.495, 0.082)	(-0.527, 0.061)
Maltose	-0.119	0.573	0.022	0.017	0.155	0.043
	(-0.344, 0.112)	(0.202, 0.954)	(-0.348, 0.406)	(-0.331, 0.368)	(-0.137, 0.448)	(-0.255, 0.336)
Proline	0.196	-0.554	-0.134	0.291	0.029	0.122
	(-0.169, 0.573)	(-1.391, 0.061)	(-0.711, 0.442)	(-0.278, 0.862)	(-0.444, 0.504)	(-0.352, 0.606)
Choline, Phosphatidylcholine	0.516	0.065	0.801	-0.266	0.223	0.255
	(0.071, 0.963)	(-0.62, 0.838)	(0.108, 1.502)	(-0.964, 0.43)	(-0.346, 0.8)	(-0.33, 0.846)
Total Choline, no betaine	-0.484	-1.481	-0.784	0.706	-0.387	-0.324
	(-1.063, 0.101)	(-2.476, -0.632)	(-1.689, 0.108)	(-0.207, 1.635)	(-1.134, 0.341)	(-1.092, 0.432)

	Fcl	Prb	Als	Odr	Brn	Phs
Intercept	1.043	1.181	1.284	0.04	0.15	-1.144
	(0.643, 1.413)	(0.774, 1.549)	(0.89, 1.652)	(-0.461, 0.529)	(-0.364, 0.629)	(-1.807, -0.533)
Sex	-0.175	-0.132	-0.144	-0.343	-0.499	0.5
	(-0.6, 0.241)	(-0.546, 0.278)	(-0.556, 0.261)	(-0.854, 0.152)	(-1.107, 0.104)	(-0.174, 1.183)
Phosphorous	0.609	0.575	0.06	-0.118	0.006	0.193
	(-0.051, 1.264)	(-0.066, 1.211)	(-0.589, 0.697)	(-0.886, 0.637)	(-0.918, 0.94)	(-0.718, 1.067)
Vitamin B12	0.041	-0.21	-0.038	-0.742	-0.322	-0.03
	(-0.282, 0.42)	(-0.609, 0.222)	(-0.451, 0.402)	(-2.017, 0.142)	(-1.118, 0.307)	(-0.506, 0.438)
Sodium	0.134	0.481	-0.039	-0.001	0.332	0.222
	(-0.123, 0.39)	(0.226, 0.736)	(-0.288, 0.212)	(-0.312, 0.308)	(-0.005, 0.663)	(-0.174, 0.611)
Manganese	-0.059	0.006	0.032	0.061	0.168	-0.057
	(-0.409, 0.305)	(-0.344, 0.354)	(-0.293, 0.366)	(-0.325, 0.447)	(-0.305, 0.636)	(-0.565, 0.456)
Iodine	-0.105	0.249	0.069	-0.1	0.125	-0.988
	(-0.346, 0.143)	(0.034, 0.471)	(-0.151, 0.294)	(-0.379, 0.175)	(-0.236, 0.479)	(-1.748, -0.384)
Riboflavin B2 w/o vit. pills	-0.239	-0.234	0.141	0.296	-0.164	-0.133
	(-0.569, 0.092)	(-0.537, 0.073)	(-0.163, 0.447)	(-0.074, 0.669)	(-0.622, 0.292)	(-0.593, 0.319)
Pyridoxine B6 w/o vit. pills	0.026	-0.249	-0.213	-0.238	0.182	-0.357
	(-0.243, 0.302)	(-0.541, 0.037)	(-0.455, 0.038)	(-0.542, 0.062)	(-0.158, 0.52)	(-0.742, 0.012)
Maltose	-0.021	-0.062	0.088	-0.139	0.199	-0.207
	(-0.295, 0.26)	(-0.324, 0.202)	(-0.171, 0.352)	(-0.458, 0.184)	(-0.182, 0.582)	(-0.608, 0.196)
Proline	-0.423	-0.128	0.248	0.196	0.011	-0.009
	(-0.878, 0.036)	(-0.566, 0.313)	(-0.175, 0.676)	(-0.31, 0.71)	(-0.624, 0.64)	(-0.649, 0.635)
Choline, Phosphatidylcholine	0.222	0.598	0.425	0.465	0.049	-0.332
	(-0.338, 0.773)	(0.047, 1.148)	(-0.113, 0.963)	(-0.181, 1.113)	(-0.702, 0.791)	(-1.103, 0.439)
Total Choline, no betaine	-0.268	-0.653	-0.428	-0.568	0.107	0.365
	(-0.984, 0.462)	(-1.347, 0.056)	(-1.139, 0.276)	(-1.404, 0.263)	(-0.823, 1.049)	(-0.636, 1.378)

Table S30: COMBO application. Posterior means and 90% CSs for the regression coefficients of the FDMReg model.

	Bct	Prv	Rmn	Prs	Osc	Sbd
Intercept	3.271	-0.35	-0.219	-0.325	0.478	0.287
	(2.96, 3.574)	(-0.953, 0.195)	(-0.778, 0.428)	(-0.914, 0.25)	(0.092, 0.851)	(-0.158, 0.781)
Sex	0.095	-1.685	0.106	0.094	0.139	0.127
	(-0.246, 0.438)	(-2.257, -1.13)	(-0.429, 0.634)	(-0.518, 0.695)	(-0.292, 0.583)	(-0.326, 0.576)
Phosphorous	0.314	2.06	0.497	-0.499	0.33	0.135
	(-0.21, 0.843)	(0.873, 3.28)	(-0.341, 1.334)	(-1.384, 0.368)	(-0.347, 1.019)	(-0.566, 0.839)
Vitamin B12	0.174	-2.746	0.316	0.252	0.179	0.226
	(-0.094, 0.524)	(-4.953, -0.864)	(-0.045, 0.715)	(-0.142, 0.681)	(-0.15, 0.566)	(-0.1, 0.606)
Sodium	0.119	-0.262	-0.09	0.13	-0.104	-0.093
	(-0.089, 0.335)	(-0.616, 0.098)	(-0.432, 0.26)	(-0.204, 0.462)	(-0.372, 0.165)	(-0.358, 0.176)
Manganese	0.038	-0.413	-0.275	0.086	-0.036	0.001
	(-0.258, 0.341)	(-0.979, 0.113)	(-0.723, 0.157)	(-0.347, 0.531)	(-0.402, 0.33)	(-0.358, 0.361)
Iodine	-0.015	0.081	-0.013	0.092	0.053	-0.015
	(-0.199, 0.179)	(-0.18, 0.35)	(-0.316, 0.269)	(-0.217, 0.384)	(-0.184, 0.294)	(-0.258, 0.225)
Riboflavin B2 w/o vit. pills	-0.242	-0.803	0.125	-0.026	0.044	-0.121
	(-0.487, 0.008)	(-1.274, -0.339)	(-0.269, 0.524)	(-0.431, 0.393)	(-0.276, 0.364)	(-0.464, 0.223)
Pyridoxine B6 w/o vit. pills	-0.174	0.959	-0.197	-0.036	-0.201	-0.222
	(-0.38, 0.058)	(0.555, 1.41)	(-0.507, 0.124)	(-0.36, 0.286)	(-0.486, 0.092)	(-0.511, 0.071)
Maltose	-0.116	0.604	0.006	0.017	0.135	0.054
	(-0.338, 0.109)	(0.157, 1.046)	(-0.358, 0.368)	(-0.347, 0.374)	(-0.148, 0.423)	(-0.24, 0.34)
Proline	0.193	-1.241	-0.117	0.278	0.037	0.116
	(-0.171, 0.556)	(-1.979, -0.454)	(-0.686, 0.44)	(-0.29, 0.85)	(-0.437, 0.504)	(-0.348, 0.594)
Choline, Phosphatidylcholine	0.526	0.441	0.812	-0.238	0.237	0.254
	(0.078, 0.98)	(-0.365, 1.24)	(0.112, 1.514)	(-0.97, 0.469)	(-0.334, 0.815)	(-0.34, 0.846)
Total Choline, no betaine	-0.514	-1.979	-0.804	0.651	-0.406	-0.327
	(-1.107, 0.066)	(-3.013, -1)	(-1.715, 0.116)	(-0.262, 1.614)	(-1.151, 0.329)	(-1.092, 0.439)

	Fcl	Prb	Als	Odr	Brn	Phs
Intercept	1.045	1.205	1.274	0.039	0.162	-0.977
	(0.678, 1.404)	(0.839, 1.561)	(0.909, 1.631)	(-0.435, 0.512)	(-0.309, 0.622)	(-1.665, -0.33)
Sex	-0.174	-0.16	-0.142	-0.344	-0.513	0.446
	(-0.591, 0.248)	(-0.572, 0.248)	(-0.554, 0.261)	(-0.841, 0.156)	(-1.126, 0.086)	(-0.271, 1.171)
Phosphorous	0.579	0.553	0.022	-0.112	-0.116	0.211
	(-0.083, 1.242)	(-0.072, 1.188)	(-0.615, 0.672)	(-0.87, 0.651)	(-1.023, 0.791)	(-0.733, 1.139)
Vitamin B12	0.038	-0.224	-0.019	-0.699	-0.333	-0.042
	(-0.277, 0.414)	(-0.632, 0.213)	(-0.47, 0.426)	(-1.907, 0.125)	(-1.143, 0.293)	(-0.512, 0.43)
Sodium	0.137	0.482	-0.027	0.005	0.32	0.217
	(-0.116, 0.397)	(0.225, 0.742)	(-0.274, 0.217)	(-0.295, 0.307)	(-0.018, 0.655)	(-0.194, 0.615)
Manganese	-0.048	0.006	0.013	0.06	0.212	-0.087
	(-0.41, 0.313)	(-0.343, 0.357)	(-0.323, 0.35)	(-0.331, 0.448)	(-0.269, 0.68)	(-0.625, 0.455)
Iodine	-0.101	0.251	0.07	-0.094	0.121	-0.998
	(-0.339, 0.135)	(0.039, 0.469)	(-0.15, 0.294)	(-0.354, 0.17)	(-0.229, 0.461)	(-1.739, -0.372)
Riboflavin B2 w/o vit. pills	-0.24	-0.232	0.158	0.282	-0.2	-0.122
	(-0.568, 0.082)	(-0.534, 0.072)	(-0.143, 0.46)	(-0.076, 0.646)	(-0.651, 0.248)	(-0.603, 0.339)
Pyridoxine B6 w/o vit. pills	0.022	-0.241	-0.22	-0.23	0.209	-0.346
	(-0.247, 0.305)	(-0.532, 0.051)	(-0.461, 0.039)	(-0.537, 0.078)	(-0.128, 0.547)	(-0.751, 0.034)
Maltose	-0.031	-0.061	0.078	-0.136	0.23	-0.224
	(-0.31, 0.247)	(-0.326, 0.199)	(-0.18, 0.342)	(-0.454, 0.177)	(-0.152, 0.608)	(-0.638, 0.192)
Proline	-0.394	-0.117	0.235	0.196	0.103	-0.039
	(-0.849, 0.061)	(-0.547, 0.313)	(-0.189, 0.659)	(-0.305, 0.689)	(-0.508, 0.712)	(-0.72, 0.649)
Choline, Phosphatidylcholine	0.235	0.593	0.394	0.454	-0.037	-0.337
	(-0.331, 0.804)	(0.047, 1.148)	(-0.143, 0.937)	(-0.181, 1.096)	(-0.787, 0.706)	(-1.139, 0.451)
Total Choline, no betaine	-0.285	-0.649	-0.381	-0.564	0.196	0.372
	(-1.017, 0.455)	(-1.344, 0.04)	(-1.089, 0.318)	(-1.402, 0.258)	(-0.734, 1.137)	(-0.646, 1.405)

Table S31: COMBO application. Posterior means and 90% CSs for the regression coefficients of the DMReg model.

	Bct	Prv	Rmn	Prs	Osc	Sbd
Intercept	3.058	-0.557	-0.364	-0.585	0.442	0.212
	(2.779, 3.352)	(-1.114, -0.034)	(-0.771, 0.045)	(-0.996, -0.176)	(0.086, 0.801)	(-0.166, 0.588)
Sex	0.127	-3.047	0.122	0.218	0.147	0.088
	(-0.249, 0.49)	(-3.748, -2.373)	(-0.417, 0.668)	(-0.329, 0.765)	(-0.325, 0.624)	(-0.425, 0.596)
Phosphorous	0.187	2.507	0.432	-0.381	0.28	0.035
	(-0.398, 0.766)	(1.58, 3.456)	(-0.435, 1.31)	(-1.231, 0.482)	(-0.473, 1.034)	(-0.749, 0.835)
Vitamin B12	0.207	-3.271	0.347	0.248	0.202	0.276
	(-0.087, 0.579)	(-5.286, -1.437)	(-0.035, 0.776)	(-0.153, 0.682)	(-0.159, 0.614)	(-0.089, 0.685)
Sodium	0.193	-0.027	-0.089	0.112	-0.087	-0.093
	(-0.034, 0.418)	(-0.39, 0.338)	(-0.439, 0.265)	(-0.222, 0.441)	(-0.384, 0.213)	(-0.395, 0.206)
Manganese	0.105	-0.631	-0.254	0.028	-0.013	0.012
	(-0.209, 0.423)	(-1.139, -0.125)	(-0.712, 0.19)	(-0.406, 0.456)	(-0.411, 0.376)	(-0.385, 0.405)
Iodine	0.032	-0.118	0.022	0.109	0.061	-0.023
	(-0.172, 0.245)	(-0.51, 0.258)	(-0.276, 0.323)	(-0.191, 0.399)	(-0.199, 0.326)	(-0.309, 0.258)
Riboflavin B2 w/o vit. pills	-0.256	-0.811	0.107	-0.041	0.03	-0.072
	(-0.532, 0.025)	(-1.378, -0.245)	(-0.298, 0.513)	(-0.445, 0.362)	(-0.329, 0.392)	(-0.462, 0.317)
Pyridoxine B6 w/o vit. pills	-0.176	1.631	-0.154	-0.04	-0.179	-0.212
	(-0.414, 0.08)	(1.16, 2.069)	(-0.488, 0.18)	(-0.378, 0.29)	(-0.507, 0.146)	(-0.55, 0.124)
Maltose	-0.153	0.816	-0.008	0.042	0.115	0.045
	(-0.399, 0.094)	(0.316, 1.311)	(-0.387, 0.364)	(-0.312, 0.4)	(-0.208, 0.435)	(-0.282, 0.374)
Proline	0.277	-2.087	-0.056	0.225	0.069	0.144
	(-0.135, 0.687)	(-2.772, -1.411)	(-0.655, 0.527)	(-0.364, 0.801)	(-0.464, 0.591)	(-0.391, 0.682)
Choline, Phosphatidylcholine	0.473	0.74	0.765	-0.296	0.213	0.216
	(-0.013, 0.954)	(-0.029, 1.521)	(0.06, 1.471)	(-0.989, 0.391)	(-0.406, 0.824)	(-0.428, 0.867)
Total Choline, no betaine	-0.398	-2.359	-0.772	0.645	-0.37	-0.291
	(-1.017, 0.231)	(-3.253, -1.469)	(-1.689, 0.14)	(-0.256, 1.555)	(-1.175, 0.439)	(-1.149, 0.539)

	Fcl	Prb	Als	Odr	Brn	Phs
Intercept	0.909	1.05	1.109	-0.03	-0.305	-1.379
	(0.559, 1.264)	(0.712, 1.393)	(0.768, 1.457)	(-0.454, 0.377)	(-0.751, 0.127)	(-1.933, -0.845)
Sex	-0.107	-0.099	-0.107	-0.322	-0.461	0.593
	(-0.575, 0.362)	(-0.555, 0.353)	(-0.565, 0.346)	(-0.848, 0.212)	(-1.055, 0.127)	(-0.026, 1.234)
Phosphorous	0.49	0.596	-0.042	-0.132	0.061	0.146
	(-0.257, 1.227)	(-0.106, 1.298)	(-0.764, 0.678)	(-0.962, 0.694)	(-0.914, 1.021)	(-0.744, 1.039)
Vitamin B12	0.092	-0.107	0.034	-0.821	-0.286	0.007
	(-0.264, 0.499)	(-0.539, 0.336)	(-0.414, 0.499)	(-2.213, 0.139)	(-1.049, 0.323)	(-0.445, 0.478)
Sodium	0.136	0.42	-0.047	-0.011	0.266	0.166
	(-0.152, 0.415)	(0.139, 0.699)	(-0.323, 0.231)	(-0.336, 0.31)	(-0.063, 0.591)	(-0.212, 0.538)
Manganese	-0.023	0.003	0.022	0.06	0.111	-0.06
	(-0.416, 0.376)	(-0.383, 0.384)	(-0.353, 0.394)	(-0.357, 0.472)	(-0.347, 0.575)	(-0.556, 0.431)
Iodine	-0.08	0.239	0.063	-0.091	0.076	-0.921
	(-0.352, 0.187)	(-0.005, 0.486)	(-0.189, 0.321)	(-0.388, 0.199)	(-0.272, 0.407)	(-1.624, -0.35)
Riboflavin B2 w/o vit. pills	-0.221	-0.264	0.162	0.276	-0.264	-0.106
	(-0.588, 0.146)	(-0.603, 0.082)	(-0.182, 0.505)	(-0.111, 0.664)	(-0.723, 0.195)	(-0.541, 0.327)
Pyridoxine B6 w/o vit. pills	0.02	-0.212	-0.194	-0.204	0.215	-0.309
	(-0.29, 0.338)	(-0.538, 0.112)	(-0.475, 0.097)	(-0.545, 0.133)	(-0.146, 0.573)	(-0.693, 0.058)
Maltose	-0.017	-0.044	0.084	-0.134	0.226	-0.151
	(-0.322, 0.29)	(-0.337, 0.253)	(-0.21, 0.385)	(-0.475, 0.209)	(-0.144, 0.589)	(-0.544, 0.23)
Proline	-0.306	-0.086	0.257	0.218	0.071	0.013
	(-0.821, 0.208)	(-0.578, 0.403)	(-0.229, 0.742)	(-0.329, 0.758)	(-0.572, 0.724)	(-0.623, 0.648)
Choline, Phosphatidylcholine	0.215	0.601	0.346	0.416	-0.059	-0.313
	(-0.401, 0.835)	(0.004, 1.209)	(-0.249, 0.939)	(-0.271, 1.091)	(-0.777, 0.66)	(-1.054, 0.43)
Total Choline, no betaine	-0.248	-0.706	-0.328	-0.547	0.141	0.328
	(-1.052, 0.567)	(-1.482, 0.058)	(-1.114, 0.447)	(-1.431, 0.352)	(-0.793, 1.079)	(-0.627, 1.32)

Table S32: COMBO application. Posterior means and 90% CSs for the regression coefficients of the EFDMReg model for the subgroup with BMI ≥ 25.

	Bct	Prv	Rmn	Prs	Osc	Sbd
Intercept	3.395	-1.439	-0.597	-0.486	0.631	0.7
	(2.759, 4.052)	(-2.792, -0.197)	(-1.518, 0.305)	(-1.343, 0.351)	(-0.167, 1.434)	(-0.065, 1.472)
Sex	0.199	-3.377	-0.235	0.156	-0.153	-0.346
	(-0.461, 0.872)	(-5.044, -1.857)	(-1.235, 0.817)	(-0.844, 1.184)	(-1.022, 0.718)	(-1.185, 0.485)
Phosphorous	0.616	4.086	-0.776	-2.272	0.628	0.338
	(-0.682, 1.899)	(1.582, 6.696)	(-2.887, 1.494)	(-4.265, -0.295)	(-1.056, 2.275)	(-1.227, 1.87)
Vitamin B12	1.311	-2.226	2.322	1.652	1.365	1.476
	(0.013, 3.138)	(-6.573, 1.069)	(0.838, 4.225)	(0.197, 3.587)	(-0.002, 3.223)	(0.132, 3.324)
Sodium	0.443	0.472	-0.773	0.248	-0.025	0.219
	(-0.171, 1.066)	(-0.791, 1.744)	(-1.792, 0.186)	(-0.747, 1.209)	(-0.818, 0.765)	(-0.516, 0.949)
Manganese	-0.108	0.239	-0.338	0.736	-0.074	0.136
	(-0.74, 0.554)	(-1.123, 1.596)	(-1.609, 0.867)	(-0.313, 1.838)	(-0.842, 0.7)	(-0.618, 0.914)
Iodine	0.229	-1.382	-0.284	-0.578	0.154	-0.298
	(-0.234, 0.722)	(-2.333, -0.462)	(-1.047, 0.457)	(-1.587, 0.336)	(-0.465, 0.756)	(-0.94, 0.305)
Riboflavin B2 w/o vit. pills	-0.678	0.832	0.52	0.259	-0.302	0.163
	(-1.452, 0.083)	(-0.676, 2.226)	(-0.731, 1.711)	(-0.915, 1.462)	(-1.253, 0.653)	(-0.74, 1.068)
Pyridoxine B6 w/o vit. pills	0.129	0.549	-2.143	0.752	-0.519	-0.405
	(-0.564, 0.827)	(-0.787, 1.941)	(-3.74, -0.582)	(-0.339, 1.834)	(-1.509, 0.454)	(-1.326, 0.508)
Maltose	0.049	1.127	0.456	0.39	0.343	-0.5
	(-0.427, 0.559)	(-0.046, 2.291)	(-0.438, 1.317)	(-0.378, 1.159)	(-0.327, 1.008)	(-1.205, 0.203)
Proline	-0.202	-4.868	0.993	0.784	0.091	-0.193
	(-1.114, 0.71)	(-7.016, -2.821)	(-0.435, 2.338)	(-0.605, 2.168)	(-1.031, 1.208)	(-1.256, 0.863)
Choline, Phosphatidylcholine	0.234	4.077	0.597	-0.853	-0.416	0.113
	(-0.696, 1.15)	(1.982, 6.36)	(-0.891, 2.169)	(-2.358, 0.673)	(-1.584, 0.775)	(-1.083, 1.351)
Total Choline, no betaine	0.151	-4.999	-0.265	2.262	0.464	-0.756
	(-0.957, 1.31)	(-7.651, -2.584)	(-2.137, 1.489)	(0.381, 4.159)	(-1.01, 1.926)	(-2.229, 0.654)

	Fcl	Prb	Als	Odr	Brn	Phs
Intercept	1.647	1.345	1.22	-0.011	-0.11	-3.952
	(0.915, 2.375)	(0.667, 2.05)	(0.425, 1.989)	(-0.99, 0.916)	(-1.12, 0.822)	(-6.339, -1.955)
Sex	-1.125	0.302	-0.304	-0.492	-1.409	0.913
	(-1.949, -0.263)	(-0.426, 1.028)	(-1.051, 0.445)	(-1.452, 0.458)	(-2.509, -0.356)	(-0.404, 2.234)
Phosphorous	2.475	-0.052	-0.118	-1.023	-2.624	1.131
	(0.812, 4.143)	(-1.409, 1.284)	(-1.568, 1.287)	(-2.86, 0.766)	(-4.668, -0.627)	(-1.841, 4.219)
Vitamin B12	1.399	1.551	-1.327	-2.23	-1.27	-3.071
	(0.058, 3.249)	(0.236, 3.394)	(-4.585, 1.422)	(-6.602, 1.215)	(-5.543, 2.102)	(-8.682, 1.269)
Sodium	0.894	0.532	-0.479	-0.881	-1.274	1.061
	(0.216, 1.569)	(-0.131, 1.209)	(-1.18, 0.219)	(-1.935, 0.142)	(-2.163, -0.411)	(-0.168, 2.299)
Manganese	-0.996	0.38	0.581	1.017	1.311	-0.384
	(-1.792, -0.218)	(-0.315, 1.12)	(-0.118, 1.305)	(0.163, 1.908)	(0.293, 2.362)	(-2.302, 1.404)
Iodine	0.311	0.084	0.331	0.175	0.45	-4.492
	(-0.216, 0.852)	(-0.436, 0.608)	(-0.175, 0.849)	(-0.467, 0.802)	(-0.511, 1.316)	(-9.297, -1.122)
Riboflavin B2 w/o vit. pills	-0.788	-0.156	-0.014	-0.326	-0.91	1.084
	(-1.65, 0.052)	(-0.96, 0.652)	(-0.805, 0.789)	(-1.327, 0.685)	(-1.974, 0.136)	(-0.347, 2.513)
Pyridoxine B6 w/o vit. pills	-0.148	-0.593	-1.438	-1.24	-1.003	-0.412
	(-0.972, 0.697)	(-1.413, 0.22)	(-2.308, -0.571)	(-2.306, -0.18)	(-2.466, 0.451)	(-1.964, 1.028)
Maltose	-0.23	-0.159	0.477	0.583	1.304	-1.408
	(-0.844, 0.406)	(-0.791, 0.483)	(-0.097, 1.068)	(-0.182, 1.337)	(0.469, 2.134)	(-2.629, -0.222)
Proline	-1.298	0.043	1.22	1.794	3.706	-0.73
	(-2.331, -0.261)	(-0.906, 0.999)	(0.276, 2.177)	(0.505, 3.128)	(2.417, 5.016)	(-2.646, 1.119)
Choline, Phosphatidylcholine	-0.368	0.158	0.35	0.049	-0.334	0.889
	(-1.464, 0.695)	(-0.85, 1.161)	(-0.655, 1.363)	(-1.304, 1.425)	(-1.707, 1.033)	(-1.053, 2.908)
Total Choline, no betaine	-0.12	0.07	-0.008	0.026	0.837	-0.825
	(-1.46, 1.345)	(-1.14, 1.29)	(-1.315, 1.307)	(-1.703, 1.732)	(-0.895, 2.567)	(-3.278, 1.543)

Table S33: COMBO application. Posterior means and 90% CSs for the regression coefficients of the EFDMReg model for the subgroup with BMI < 25.

	Bct	Prv	Rmn	Prs	Osc	Sbd
Intercept	3.11	-0.611	-0.339	-0.647	0.489	-0.026
	(2.561, 3.617)	(-1.403, 0.128)	(-1.102, 0.393)	(-1.418, 0.089)	(-0.13, 1.084)	(-0.704, 0.622)
Sex	0.212	-1.493	0.324	0.452	0.232	0.457
	(-0.271, 0.689)	(-2.217, -0.819)	(-0.451, 1.105)	(-0.334, 1.261)	(-0.372, 0.83)	(-0.191, 1.108)
Phosphorous	0.262	0.277	0.516	0.043	0.541	0.313
	(-0.465, 0.987)	(-0.673, 1.215)	(-0.642, 1.685)	(-1.083, 1.155)	(-0.366, 1.441)	(-0.671, 1.306)
Vitamin B12	-0.053	-3.459	0.005	0.089	-0.017	0.001
	(-0.346, 0.32)	(-5.665, -1.481)	(-0.414, 0.422)	(-0.392, 0.521)	(-0.363, 0.363)	(-0.351, 0.385)
Sodium	0.196	-0.032	-0.073	0.06	-0.142	-0.207
	(-0.07, 0.469)	(-0.431, 0.427)	(-0.516, 0.373)	(-0.329, 0.447)	(-0.456, 0.172)	(-0.536, 0.124)
Manganese	-0.117	-0.027	-0.283	-0.323	-0.349	-0.303
	(-0.518, 0.306)	(-0.598, 0.538)	(-0.895, 0.334)	(-0.944, 0.291)	(-0.854, 0.163)	(-0.819, 0.222)
Iodine	0.006	0.228	0.009	0.134	0.06	0.037
	(-0.223, 0.258)	(-0.03, 0.514)	(-0.353, 0.365)	(-0.235, 0.5)	(-0.222, 0.356)	(-0.275, 0.354)
Riboflavin B2 w/o vit. pills	-0.123	-0.872	0.093	0.047	0.112	-0.234
	(-0.423, 0.181)	(-1.405, -0.23)	(-0.38, 0.559)	(-0.44, 0.526)	(-0.252, 0.478)	(-0.648, 0.179)
Pyridoxine B6 w/o vit. pills	-0.223	0.925	-0.116	-0.138	-0.168	-0.128
	(-0.479, 0.053)	(0.425, 1.565)	(-0.462, 0.255)	(-0.534, 0.257)	(-0.492, 0.17)	(-0.478, 0.227)
Maltose	-0.207	0.402	0.04	-0.218	0.076	0.208
	(-0.517, 0.087)	(-0.021, 0.81)	(-0.46, 0.541)	(-0.706, 0.267)	(-0.315, 0.459)	(-0.195, 0.612)
Proline	0.269	-0.269	-0.18	0.1	-0.135	0.07
	(-0.198, 0.745)	(-0.909, 0.348)	(-0.914, 0.564)	(-0.667, 0.876)	(-0.739, 0.46)	(-0.553, 0.696)
Choline, Phosphatidylcholine	0.34	-0.507	0.594	0.18	0.416	0.26
	(-0.27, 0.957)	(-1.407, 0.321)	(-0.367, 1.558)	(-0.8, 1.181)	(-0.321, 1.174)	(-0.549, 1.076)
Total Choline, no betaine	-0.39	-0.801	-0.569	-0.096	-0.653	-0.202
	(-1.156, 0.371)	(-1.819, 0.327)	(-1.804, 0.677)	(-1.359, 1.15)	(-1.603, 0.265)	(-1.247, 0.832)

	Fcl	Prb	Als	Odr	Brn	Phs
Intercept	1.001	0.84	1.423	-0.034	-0.11	-0.813
	(0.403, 1.562)	(0.195, 1.443)	(0.845, 1.962)	(-0.811, 0.711)	(-0.869, 0.637)	(-1.7, 0.01)
Sex	-0.127	-0.109	-0.18	-0.236	-0.241	0.275
	(-0.712, 0.451)	(-0.675, 0.456)	(-0.734, 0.367)	(-0.992, 0.513)	(-1.067, 0.571)	(-0.613, 1.2)
Phosphorous	0.246	0.91	0.361	0.285	1.721	0.63
	(-0.609, 1.083)	(0.082, 1.742)	(-0.483, 1.2)	(-0.733, 1.294)	(0.36, 3.036)	(-0.437, 1.676)
Vitamin B12	-0.147	-1.716	0.157	-1.269	-0.798	-0.206
	(-0.525, 0.235)	(-3.522, -0.437)	(-0.131, 0.508)	(-3.073, -0.105)	(-1.773, -0.109)	(-0.63, 0.222)
Sodium	0.024	0.4	-0.055	0.026	0.227	0.007
	(-0.269, 0.318)	(0.098, 0.713)	(-0.338, 0.231)	(-0.334, 0.386)	(-0.161, 0.612)	(-0.455, 0.467)
Manganese	0.106	-0.322	-0.315	-0.492	-0.756	-0.363
	(-0.363, 0.582)	(-0.801, 0.173)	(-0.794, 0.164)	(-1.044, 0.065)	(-1.41, -0.092)	(-0.997, 0.274)
Iodine	-0.095	0.326	0.062	-0.175	0.105	-0.813
	(-0.375, 0.195)	(0.072, 0.598)	(-0.198, 0.336)	(-0.554, 0.183)	(-0.406, 0.568)	(-1.537, -0.223)
Riboflavin B2 w/o vit. pills	-0.197	-0.225	0.155	0.364	-0.367	-0.209
	(-0.564, 0.17)	(-0.579, 0.128)	(-0.184, 0.498)	(-0.061, 0.799)	(-0.945, 0.198)	(-0.734, 0.303)
Pyridoxine B6 w/o vit. pills	-0.032	-0.14	-0.14	-0.11	0.407	-0.336
	(-0.337, 0.291)	(-0.499, 0.218)	(-0.421, 0.166)	(-0.464, 0.248)	(-0.04, 0.851)	(-0.821, 0.121)
Maltose	-0.103	-0.054	0.09	-0.12	0.466	-0.047
	(-0.46, 0.246)	(-0.402, 0.29)	(-0.26, 0.433)	(-0.565, 0.312)	(-0.045, 0.957)	(-0.56, 0.485)
Proline	-0.041	-0.213	-0.064	-0.234	-1.108	-0.616
	(-0.604, 0.52)	(-0.756, 0.335)	(-0.594, 0.477)	(-0.895, 0.413)	(-1.963, -0.229)	(-1.39, 0.157)
Choline, Phosphatidylcholine	0.395	0.906	0.609	0.626	0.002	-0.208
	(-0.327, 1.139)	(0.186, 1.649)	(-0.073, 1.315)	(-0.213, 1.483)	(-0.984, 0.97)	(-1.178, 0.753)
Total Choline, no betaine	-0.402	-0.977	-0.65	-0.646	-0.25	0.284
	(-1.333, 0.503)	(-1.878, -0.089)	(-1.536, 0.227)	(-1.738, 0.426)	(-1.434, 0.96)	(-0.946, 1.524)

5.2.2 Posterior predictive checks

To assess the predictive performance of the EFDMReg model, we performed posterior predictive checks. Specifically, we compared the posterior predictive distribution of the relative abundance of a given taxon in a specific subject to the corresponding observed value.

Having the design matrix $\mathbf{X}$ and samples from the posterior distribution of the model parameters, namely $\boldsymbol{\beta}_r^{(b)}, {\alpha^+}^{(b)}, \mathbf{p}^{(b)}$, and $\tilde{\mathbf{w}}^{(b)}$ for $b = 1, \ldots, B$ and $r = 1, \ldots, D-1$, we can generate draws from the posterior predictive distribution.

More precisely, we generate B replicates $\mathbf{Y}_i^{(b)}$ from the model

$$\mathbf{Y}_i^{(b)} \sim EFDM\left(n_i, \boldsymbol{\mu}_i^{(b)}, {\alpha^+}^{(b)}, \mathbf{p}^{(b)}, \tilde{\mathbf{w}}^{(b)}\right), \qquad b = 1, \ldots, B,$$

where the predictive mean vector $\boldsymbol{\mu}_i^{(b)}$ has components

$$\mu_{i,r}^{(b)} = \begin{cases} \dfrac{\exp\left(\mathbf{x}_i^\intercal \boldsymbol{\beta}_r^{(b)}\right)}{1 + \sum_{l=1}^{D-1} \exp\left(\mathbf{x}_i^\intercal \boldsymbol{\beta}_l^{(b)}\right)}, & r \neq D, \\ \dfrac{1}{1 + \sum_{l=1}^{D-1} \exp\left(\mathbf{x}_i^\intercal \boldsymbol{\beta}_l^{(b)}\right)}, & r = D. \end{cases}$$

We then compare the posterior predictive distribution of the relative counts $\mathbf{Y}_i/n_i$ with the observed proportions $\mathbf{y}_i/n_i$, typically through graphical summaries. Dividing by n_i allows us to focus on the composition, disregarding the variability due to the total number of bacterial reads.

If the model is well-specified, the observed value $\mathbf{y}_i/n_i$ should lie near the center of the posterior predictive distribution.

Figures S26, S27, and S28 show posterior predictive distributions under the DMreg and the EFDMreg models for three representative cases:

- *Bacteroides* (the most abundant taxon) in subject 1;
- *Prevotella* in subject 2;
- *Oscillobacter* (a taxon with relatively low abundance) in subject 2.

It is easy to see that the EFDMReg model typically yields posterior predictive distributions that are closer to the observed values (red-dashed lines). This improved accuracy, however, comes at the cost of heavier right tails, particularly compared to the DMReg model. Nonetheless, such heavier tails can be beneficial, as they allow the distribution to better capture extreme observed values (see Figure S27, where the observed value is poorly fitted by both models, but the EFDMReg model at least assigns it a non-negligible posterior predictive density).

The heavier tail of the posterior predictive distribution under the EFDMReg model arises from its mixture structure: for each observation (i.e., for a given

$\boldsymbol{\mu}_i$), the EFDM generates data from a mixture with D DM components, each having a different barycenter. An even better model fit could potentially be achieved by allowing the mixture weights to depend on covariates, for example, through a multinomial link model. This extension, not available for the DMReg model, could reduce the variability of the posterior predictive distributions and improve their alignment with the observed data.

Figure S26: COMBO application. Posterior predictive distribution of the proportion of *Bacteroides* for subject 1. The red-dashed line indicates the observed proportion, namely $Y_{1,1}/n_1$.

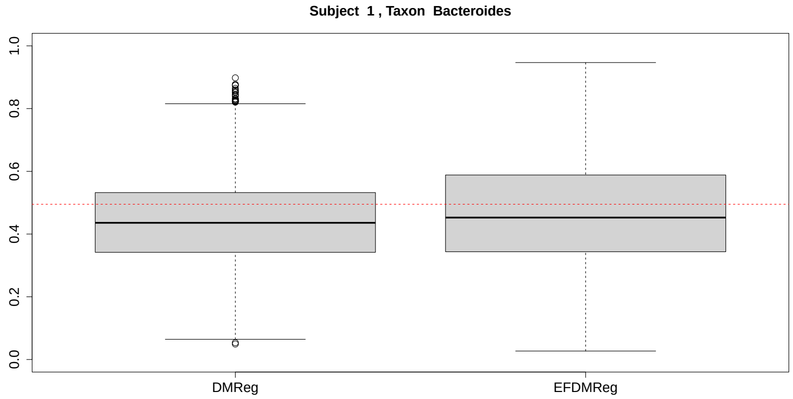

Figure S27: COMBO application. Posterior predictive distribution of the proportion of *Prevotella* for subject 2. The red-dashed line indicates the observed proportion, namely $Y_{2,2}/n_2$.

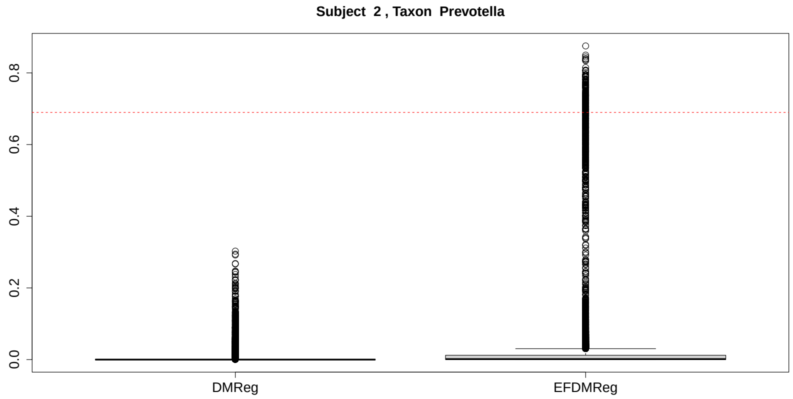

Figure S28: COMBO application. Posterior predictive distribution of the proportion of *Oscillobacter* for subject 2. The red-dashed line indicates the observed proportion, namely $Y_{2,5}/n_2$.

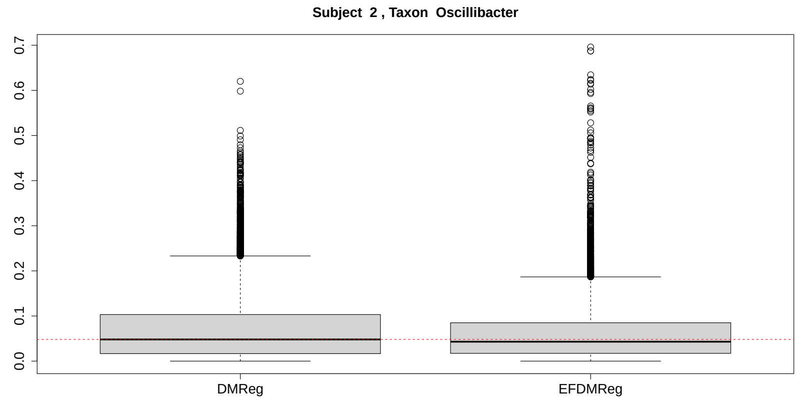

5.2.3 Estimation of ZIDM model

In this section, we report results on the fit of the ZIDM regression model by [2] to the COMBO dataset. This dataset is characterized by a proportion of zeros that varies between 0 (*Bacteroides* taxon) and 0.625 (*Prevotella* taxon), with an average proportion equal to 0.2 (see Table S21).

The comparison of the predictive ability of ZIDM and EFDMReg models is based on the measures described in Section 3 of this SM. In particular, values of the average KL divergence of the predicted from the observed responses, sensitivity, specificity, PPV, NPV, and accuracy are reported in Table S34.

Table S34: COMBO application. Comparison between EFDMReg and ZIDM regression models in terms of predictive ability ($D = 13$).

	EFDMReg	ZIDM
$d_{KL}; \varepsilon = 0.5$	82.549	328.016
$d_{KL}; \varepsilon = 1$	79.584	306.006
Sensitivity	0.440	0.908
Specificity	0.869	0.574
PPV	0.457	0.349
NPV	0.861	0.961
Accuracy	0.783	0.641

As far as zero handling is concerned, the ZIDM regression model shows larger sensitivity and NPV, while the EFDMReg deserves larger specificity and PPV. The accuracy measure points to the EFDMReg as the best model. In terms of overall predictive performance, the EFDMReg model greatly outperforms the ZIDM, as witnessed by the KL divergence.

The performance of the ZIDM model on the COMBO dataset can be further studied from the perspective of significant associations between taxa and covariates. Table S35 reports the probabilities of inclusion of the covariates that have been selected with a threshold equal to 0.5. This value for the threshold has been chosen to guarantee a number of covariates similar to the number of covariates selected by the spike and slab procedure applied to the EFDMReg model (see Table S29).

Table S35: COMBO application. Covariates selected by the ZIDM regression model for $D = 13$. A covariate is selected if its probability of inclusion is greater than 0.5 for at least one taxon. In **bold** the probabilities larger than the threshold 0.5.

$D = 13$	Bct	Prv	Rmn	Prs	Osc	Sbd	Fcl	Prb	Als	Odr	Brn	Phs	Cpr
(Intercept)	1.00	1.00	1.00	1.00	1.00	1.00	1.00	1.00	1.00	1.00	1.00	1.00	1.00
Age	0.49	**0.67**	0.26	0.13	0.22	0.16	0.27	0.21	0.31	0.19	0.36	0.23	0.10
Vegetable fat	0.33	**0.75**	0.11	0.13	0.23	0.08	0.12	0.12	0.20	0.09	0.18	0.17	0.17
Carbohydrates	0.15	**0.89**	0.23	0.10	0.21	0.07	0.10	0.08	0.13	0.08	0.14	0.11	0.08
Retinol	0.07	**0.86**	0.10	0.22	0.11	0.10	0.14	0.07	0.06	0.11	0.28	0.17	0.12
Polyunsaturated fat	0.14	**0.73**	0.26	0.09	0.06	0.08	0.06	0.08	0.23	0.08	0.20	0.14	0.07
Methionine	0.11	**0.68**	0.13	0.12	0.10	0.10	0.10	0.10	0.16	0.10	0.14	0.16	0.09
Sodium	0.17	**0.63**	0.12	0.08	0.14	0.06	0.06	0.09	0.11	0.06	0.12	0.10	0.05
Omega 3,no alpha-linolenic acid	**0.80**	0.30	0.22	0.14	0.11	0.08	0.11	0.10	0.34	0.13	0.19	0.15	0.10
Caprylic fatty acid	0.21	**0.75**	0.10	0.11	0.06	0.09	0.17	0.06	0.11	0.12	0.13	0.12	0.10
Total Sugars	**0.63**	0.46	0.13	0.07	0.07	0.06	0.21	0.07	0.18	0.06	0.08	0.09	0.08
Phenylalanine, Aspartame	0.44	**0.63**	0.09	0.11	0.09	0.12	0.07	0.12	0.08	0.09	0.17	0.12	0.13
Catechin, flavan-3-ol	0.36	**0.71**	0.09	0.06	0.13	0.06	0.11	0.05	0.09	0.05	0.16	0.12	0.08
Proanthocyanidin, monomers	0.04	**0.62**	0.16	0.31	0.11	0.14	0.06	0.06	0.18	0.10	0.21	0.15	0.09

From Table S35, one immediately notes that the only taxa showing a significant association with the selected covariates are *Bacteroides* (2 hits) and *Prevotella* (11 hits), all the other taxa thus not being influenced/explained by the selected covariates. Moreover, the estimated regression coefficients (Table S36), show discrepancies with well-established associations between *Prevotella* and carbohydrate diet on the one side and between *Bacteroides* and protein/fat/choline diet on the other (e.g. see [2]). Indeed, if we interpret the sign of the estimate as representative of the sign of the association, methionine and phenylalanine are the two amino acids selected by the model, but they show association with *Prevotella* (negative and, respectively, positive), and no significant association with *Bacteroides*. An analogous remark holds for two sources of fat (namely polyunsaturated fat and caprylic fatty acid) that show a positive association with *Prevotella* and no association with *Bacteroides*. It is also interesting to note that catechin and proanthocyanidin show a significant association with *Prevotella*, positive for the first and negative for the second, while being both flavanols present in similar fruits and contributing to the same beneficial effects, such as antioxidant and anticarcinogenic properties. These inconsistencies may be traced back to the difficulties in the interpretation of the ZIDM regression coefficients and/or in structural zero handling (see Section 3).

Table S36: COMBO application. Estimates of regression coefficients associated with covariates selected by the ZIDM regression model for $D = 13$. In **bold** the estimates of significant coefficients.

$D = 13$	Bct	Prv	Rmn	Prs	Osc	Sbd	Fcl	Prb	Als	Odr	Brn	Phs	Cpr
(Intercept)	2.33	0.69	-0.67	-0.82	-0.25	-0.52	0.08	0.27	0.38	-0.66	-0.09	-0.73	-0.80
Age	-0.06	**-1.05**	-0.18	0.01	-0.02	-0.04	-0.15	-0.07	-0.06	-0.07	-0.29	-0.05	0.01
Vegetable fat	0.12	**-1.64**	-0.02	-0.01	-0.07	0.00	0.01	0.01	0.04	0.01	0.05	0.01	-0.04
Carbohydrates	0.04	**2.12**	-0.15	-0.03	-0.09	-0.01	-0.00	0.01	-0.04	-0.00	-0.01	-0.01	-0.01
Retinol	-0.00	**1.35**	-0.00	0.09	0.02	0.02	0.01	0.00	0.01	0.02	0.03	0.02	-0.00
Polyunsaturated fat	0.03	**0.98**	0.16	0.01	-0.01	-0.00	0.00	-0.00	0.09	0.01	0.02	0.04	-0.00
Methionine	-0.01	**-0.68**	-0.03	0.01	-0.00	-0.02	-0.00	-0.02	0.06	0.01	0.05	0.01	-0.01
Sodium	-0.00	**0.62**	0.01	0.00	0.02	0.00	-0.00	-0.01	0.02	0.01	-0.02	-0.01	0.00
Omega 3,no alpha-linolenic acid	**0.31**	-0.06	-0.07	-0.04	-0.02	0.00	-0.02	0.00	-0.17	-0.01	0.09	-0.03	-0.00
Caprylic fatty acid	-0.01	**0.96**	0.01	-0.01	0.01	0.01	-0.01	-0.00	0.00	0.02	0.01	-0.01	-0.01
Total Sugars	**-0.20**	0.28	-0.03	-0.00	0.01	-0.00	-0.06	-0.00	-0.04	-0.01	-0.00	0.00	-0.01
Phenylalanine, Aspartame	0.23	**0.98**	0.01	0.01	-0.01	-0.03	-0.00	0.02	0.01	-0.01	0.07	0.01	-0.03
Catechin, flavan-3-ol	-0.07	**0.79**	0.01	-0.01	0.03	0.01	0.02	-0.00	0.00	-0.00	-0.01	-0.03	0.01
Proanthocyanidin, monomers	-0.00	**-0.69**	0.05	-0.13	-0.02	-0.03	0.01	0.00	0.04	-0.02	-0.01	0.04	0.01

6 Birds Application

In this section, we consider data from the "North American Breeding Bird Survey", which contains bird count data collected along various routes across the United States and Canada.

Each observation includes the date on which the birds were recorded. Furthermore, for each bird species, phylogenetic information is available at four taxonomic levels: species, genus, family, and order.

For the purposes of this application, we focused on the subset of observations collected in 2021—the year of the most recent survey—and restricted the data to routes located within Canada. To facilitate the analysis of broad ecological patterns, we also aggregated bird species into taxonomic orders. This choice reflects the fact that species within the same order often share key ecological traits, such as foraging strategies, habitat preferences, and responses to environmental gradients. Aggregating at the order level helps to reduce noise associated with species-specific variability and rare observations while preserving biologically meaningful structure in the data. Moreover, this level of aggregation enables the identification of general patterns across ecologically coherent groups, which might be obscured when data are analyzed at finer taxonomic resolutions.

The resulting dataset includes $N = 452$ Canadian survey routes and $D = 10$ bird orders, (namely *Charadriiformes*, *Suliformes*, *Pelecaniformes*, *Anseriformes*, *Gruiformes*, *Galliformes*, *Columbiformes*, *Accipitriformes*, *Piciformes*, and *Passeriformes*), each of which has at least 500 individuals recorded across all routes.

Figure S29 presents boxplots of the abundances of the 10 considered orders, arranged in descending order of their mean values. The average (median) number of observed birds in each route is 606.325 (561), with a standard deviation (IQR) equal to 306.525 (359.75). The maximum number of observed birds is 2642.

We fitted the DM, FDM, and EFDM models to the birds' dataset. Based on the WAIC values, the EFDM model is the best-fitting model (WAIC = 19386.6), confirming that its additional parameters and mixture structure provide a clear improvement over simpler models. Moreover, the performance gap between the DM and FDM models is negligible (WAIC equal to 19929.0 for FDM and 19928.2 for DM), with both models showing considerably worse WAIC values compared to EFDM.

Table S37 reports posterior means and CSs for the parameters of the three models. By inspecting this table, it is possible to note that the EFDM detects four main clusters (i.e., four nonempty mixture components), namely the first ($p_1 = 0.1666$), the fourth ($p_4 = 0.2831$), the fifth ($p_5 = 0.1676$), and the seventh ($p_7 = 0.3417$). These clusters refer to Canadian routes having the largest proportion of *Pelecaniformes* `https://it.wikipedia.org/wiki/Pelecaniformes`, *Galliformes* `https://it.wikipedia.org/wiki/Galliformes`, *Columbiformes* `https://it.wikipedia.org/wiki/Columbidae`, and *Piciformes* `https://it.wikipedia.org/wiki/Piciformes`, respectively.

A comparison of the FDM and EFDM models demonstrates that the superior

Figure S29: Birds application. Boxplots of the relative abundances of the $D = 10$ orders.

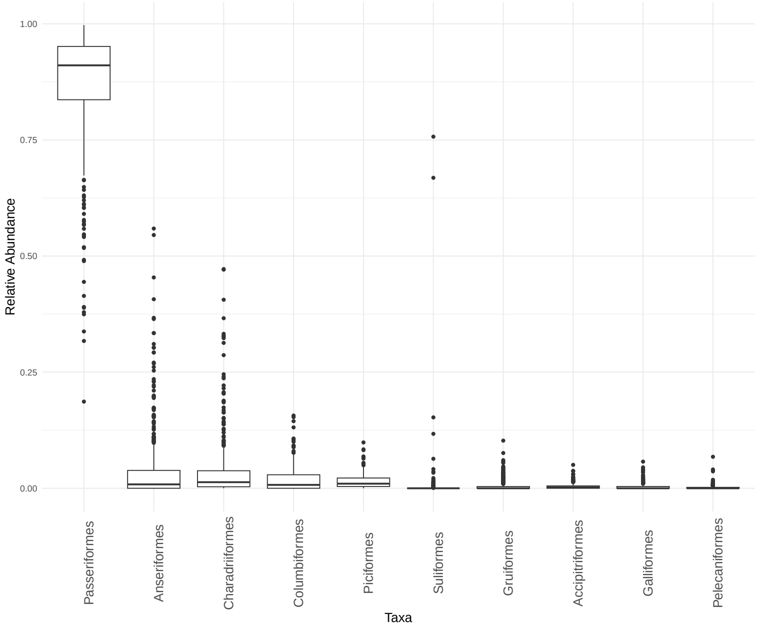

fit of the latter is attributable to its more flexible mixture structure. In the FDM model, the distances between component-specific barycentres are governed by a single global parameter ($\tilde{w} = 0.162$). This relatively low value results in clusters that are closely positioned, potentially limiting the model's expressiveness. In contrast, the EFDM model permits these distances to vary individually through a vector of weights $\tilde{\mathbf{w}}$, thereby enhancing flexibility and yielding a substantially improved fit to the data, as evidenced by the WAIC.

We also computed the correlation matrix (Table S38) among bird order counts under the EFDM model, assuming a constant number of birds observed per route equal to 600 (i.e., the average count in the dataset). Interestingly, the EFDM model identifies 26 positive correlations among the $D(D-1)/2-D = 45$ distinct pairwise correlations. One of these correlations is statistically significant in the conventional sense (i.e., the zero value is not contained in the 90% CS). However, when adopting the benchmark proposed in Section 5.1.2, the number of significant correlations increases to 17, among which 14 are positive.

Table S37: Birds application. Posterior mean and 90% CS for each model's parameter: DM (left three columns), FDM (central three columns), and EFDM (right three columns).

	Post. Mean	5%	95%	Post. Mean	5%	95%	Post. Mean	5%	95%
β_{01}	-3.344	-3.430	-3.259	-3.345	-3.431	-3.260	-3.245	-3.384	-3.102
β_{02}	-6.179	-6.431	-5.939	-6.187	-6.436	-5.949	-5.276	-6.079	-4.494
β_{03}	-4.893	-5.032	-4.758	-4.891	-5.031	-4.754	-5.262	-5.481	-4.877
β_{04}	-3.531	-3.625	-3.438	-3.530	-3.622	-3.437	-3.184	-3.337	-3.033
β_{05}	-4.788	-4.925	-4.651	-4.786	-4.926	-4.650	-5.164	-5.341	-4.943
β_{06}	-4.645	-4.772	-4.518	-4.644	-4.775	-4.517	-5.021	-5.209	-4.705
β_{07}	-3.819	-3.920	-3.719	-3.818	-3.919	-3.719	-3.915	-4.043	-3.788
β_{08}	-4.214	-4.323	-4.108	-4.213	-4.316	-4.107	-4.593	-4.751	-4.345
β_{09}	-3.516	-3.605	-3.427	-3.515	-3.604	-3.428	-3.819	-3.931	-3.679
μ_1	0.030	0.028	0.033	0.030	0.028	0.033	0.034	0.029	0.039
μ_2	0.002	0.001	0.002	0.002	0.001	0.002	0.005	0.002	0.010
μ_3	0.006	0.006	0.007	0.007	0.006	0.007	0.005	0.004	0.007
μ_4	0.025	0.023	0.028	0.025	0.023	0.028	0.036	0.031	0.041
μ_5	0.007	0.006	0.008	0.007	0.006	0.008	0.005	0.004	0.006
μ_6	0.008	0.007	0.009	0.008	0.007	0.009	0.006	0.005	0.008
μ_7	0.019	0.017	0.021	0.019	0.017	0.021	0.017	0.015	0.020
μ_8	0.013	0.011	0.014	0.013	0.012	0.014	0.009	0.007	0.011
μ_9	0.026	0.024	0.028	0.026	0.024	0.028	0.019	0.017	0.022
μ_{10}	0.863	0.856	0.869	0.863	0.856	0.869	0.865	0.855	0.874
α^+	17.619	16.527	18.747	17.599	16.483	18.730	24.559	21.887	27.201
p_1	—	—	—	0.092	0.005	0.262	0.064	0.042	0.088
p_2	—	—	—	0.168	0.013	0.388	0.006	0.001	0.012
p_3	—	—	—	0.087	0.005	0.259	0.012	0.000	0.057
p_4	—	—	—	0.094	0.006	0.270	0.115	0.083	0.150
p_5	—	—	—	0.094	0.005	0.282	0.027	0.000	0.079
p_6	—	—	—	0.094	0.005	0.276	0.013	0.000	0.058
p_7	—	—	—	0.092	0.006	0.264	0.195	0.108	0.282
p_8	—	—	—	0.085	0.004	0.256	0.010	0.000	0.051
p_9	—	—	—	0.096	0.005	0.275	0.005	0.000	0.025
p_{10}	—	—	—	0.097	0.006	0.271	0.553	0.465	0.639
$\tilde{w}_1$	—	—	—	0.162	0.008	0.524	0.385	0.300	0.467
$\tilde{w}_2$	—	—	—	—	—	—	0.776	0.670	0.895
$\tilde{w}_3$	—	—	—	—	—	—	0.311	0.006	0.912
$\tilde{w}_4$	—	—	—	—	—	—	0.581	0.510	0.644
$\tilde{w}_5$	—	—	—	—	—	—	0.186	0.016	0.731
$\tilde{w}_6$	—	—	—	—	—	—	0.299	0.006	0.900
$\tilde{w}_7$	—	—	—	—	—	—	0.380	0.244	0.506
$\tilde{w}_8$	—	—	—	—	—	—	0.371	0.006	0.927
$\tilde{w}_9$	—	—	—	—	—	—	0.405	0.005	0.941
$\tilde{w}_{10}$	—	—	—	—	—	—	0.576	0.530	0.619
WAIC	—	19928.2	—	—	19929.0	—	—	19386.6	—

Table S38: Birds application. Posterior mean and standard deviation (in parentheses) for each correlation coefficient estimated under the EFDM model. Significant correlations according to the benchmark in Section 5.1.2 are marked with an asterisk.

	Charadriif.	Sulif.	Pelecanif.	Anserif.	Gruif.	Gallif.	Columbif.	Accipitrif.	Picif.	Passerif.
Charadriiformes	1.000	-0.026	0.016	-0.021	0.023*	0.021*	0.018*	0.025*	0.045*	-0.493
	(0.000)	*(0.010)*	*(0.016)*	*(0.016)*	*(0.012)*	*(0.017)*	*(0.018)*	*(0.020)*	*(0.023)*	*(0.049)*
Suliformes	-0.026	1.000	-0.011	-0.028	-0.013*	-0.013	-0.027	-0.015	-0.024	-0.358*
	(0.010)	*(0.000)*	*(0.005)*	*(0.011)*	*(0.006)*	*(0.006)*	*(0.011)*	*(0.007)*	*(0.010)*	*(0.096)*
Pelecaniformes	0.016	-0.011	1.000	0.019	0.011	0.007	0.032	0.008	0.014	-0.177
	(0.016)	*(0.005)*	*(0.000)*	*(0.019)*	*(0.011)*	*(0.011)*	*(0.022)*	*(0.013)*	*(0.017)*	*(0.063)*
Anseriformes	-0.021	-0.028	0.019	1.000	0.023*	0.025*	-0.009	0.031*	0.054*	-0.551
	(0.016)	*(0.011)*	*(0.019)*	*(0.000)*	*(0.015)*	*(0.019)*	*(0.024)*	*(0.023)*	*(0.027)*	*(0.052)*
Gruiformes	0.023*	-0.013*	0.011	0.023*	1.000	0.013	0.042*	0.016	0.026*	-0.162
	(0.012)	*(0.006)*	*(0.011)*	*(0.015)*	*(0.000)*	*(0.012)*	*(0.016)*	*(0.014)*	*(0.018)*	*(0.041)*
Galliformes	0.021*	-0.013	0.007	0.025*	0.013	1.000	0.040*	0.010	0.018	-0.192
	(0.017)	*(0.006)*	*(0.011)*	*(0.019)*	*(0.012)*	*(0.000)*	*(0.023)*	*(0.014)*	*(0.019)*	*(0.059)*
Columbiformes	0.018*	-0.027	0.032	-0.009	0.042*	0.040*	1.000	0.049*	0.082*	-0.266*
	(0.018)	*(0.011)*	*(0.022)*	*(0.024)*	*(0.016)*	*(0.023)*	*(0.000)*	*(0.027)*	*(0.031)*	*(0.034)*
Accipitriformes	0.025*	-0.015	0.008	0.031*	0.016	0.010	0.049*	1.000	0.020	-0.237
	(0.020)	*(0.007)*	*(0.013)*	*(0.023)*	*(0.014)*	*(0.014)*	*(0.027)*	*(0.000)*	*(0.021)*	*(0.061)*
Piciformes	0.045*	-0.024	0.014	0.054*	0.026*	0.018	0.082*	0.020	1.000	-0.326
	(0.023)	*(0.010)*	*(0.017)*	*(0.027)*	*(0.018)*	*(0.019)*	*(0.031)*	*(0.021)*	*(0.000)*	*(0.056)*
Passeriformes	-0.493	-0.358*	-0.177	-0.551	-0.162	-0.192	-0.266*	-0.237	-0.326	1.000
	(0.049)	*(0.096)*	*(0.063)*	*(0.052)*	*(0.041)*	*(0.059)*	*(0.034)*	*(0.061)*	*(0.056)*	*(0.000)*

References

[1] J. Geweke. Evaluating the accuracy of sampling-based approaches to the calculation of posterior moments. *Bayesian Statistics 4*, 8(6):169–193, 2012.

[2] M.D. Koslovsky. A Bayesian zero-inflated dirichlet-multinomial regression model for multivariate compositional count data. *Biometrics*, 79:3239–3251, 2023.

[3] J. E. Mosimann. On the Compound Multinomial Distribution, the Multivariate β- Distribution, and Correlations Among Proportions. *Biometrika*, 49(1/2):65–82, 1962.

[4] A. Ongaro, S. Migliorati, and R. Ascari. A new mixture model on the simplex. *Statistics and Computing*, 30:749–770, 2020.

[5] J.D. Silverman, K. Roche, S. Mukherjee, and L.A. David. Naught all zeros in sequence count data are the same. *Computational and Structural Biotechnology Journal*, 18, 2020.
